# Dysfunctional gut microbiota and relative co-abundance network in infantile eczema

**DOI:** 10.1186/s13099-016-0118-0

**Published:** 2016-07-22

**Authors:** Heping Wang, Yinhu Li, Xin Feng, Yufeng Li, Wenjian Wang, Chuangzhao Qiu, Jianqiang Xu, Zhenyu Yang, Zhichuan Li, Qian Zhou, Kaihu Yao, Hongmei Wang, Yuzheng Li, Dongfang Li, Wenkui Dai, Yuejie Zheng

**Affiliations:** Shenzhen Children’s Hospital, No. 7019 Yitian Road, Shenzhen, 518026 China; WeHealthGene, No. 118 Wuhe Road, Shenzhen, 518129 China; Dongguan Maternal and Child Healthcare Hospital, No. 99 Zhenxing Road, Dongguan, 523000 China; Beijing Children’s Hospital, No. 56 Nanlishi Road, Beijing, 100045 China

**Keywords:** Infantile eczema, Gut microbiota, Co-abundance, Network

## Abstract

**Background:**

Infantile eczema is an immunological disease that is characterized by itchy and dry skin. Recent studies have suggested that gut microbiota (GM) plays a role in the development and progression of eczema. To further evaluate this potential link, we collected feces from 19 infants with eczema and 14 infants without eczema and analyzed the molecular discrepancies between the two groups using 16S rDNA analysis.

**Results:**

Bacteroidaceae and Deinococcaceae were significantly enriched in eczema infants, and Bacteroidaceae was potentially involved in autoimmune diseases by promoting the Th17 (T helper cell 17) secretion of IL-17 (interleukin-17). In the infants without eczema, the co-abundance network featured three core nodes: Clostridiaceae, Veillonellaceae, and Lactobacillaceae, all of which were lacking in the infants with eczema. Furthermore, our data suggested that Enterobacteriaceae was the core of the co-abundance network for the diseased subjects.

**Conclusions:**

GM is closely connected to the human immune system, and the dysbiotic GM network plays a role in eczema. This study furthered our understanding of the dynamic GM network and its correlation to the occurrence of eczema.

**Electronic supplementary material:**

The online version of this article (doi:10.1186/s13099-016-0118-0) contains supplementary material, which is available to authorized users.

## Background

Infantile eczema is a common inflammatory skin disease with a relative morbidity of 13.5 % in China [1]. The pathogenesis of infantile eczema is not well understood, and many reports have indicated that various factors such as an aberrant immune system and skin barrier dysfunction may be responsible [2]. Environmental factors, including allergens, temperature and humidity variations, solarization, and microorganisms, may also contribute to the incidence and progression of infantile eczema [2].

Recent studies have suggested that the gut microbiota (GM) may correlate with infantile eczema [3–8]. A report by Thomas et al. found that infants with eczema harbored lower GM diversity compared to their healthy counterparts [3], and Gaik et al. [4] and Hong et al. [5] further documented discrepant microbial enrichment in healthy and eczema infants, wherein *Bifidobacterium* and *Lactobacillus* were abundant in healthy infants [4, 5] while infants with eczema harbored more pathogens, including *Enterococcus*, *Klebsiella*, and *Shigella* [4, 5]. Several reports have also shown that gut bacteria interact with each other and form a dynamic network that may have substantial effects on human health [8–12]. Besides type II diabetes, rheumatoid arthritis, and inflammation [9, 10, 12], the GM network may also affect diet nutrient and immune systems in infants [11, 13]. Clarissa et al. [12] found that the decrease of *Clostridiales* was accompanied by the promotion of *Bacteroidales*, and that the up-regulated *Bacteroidales*-encoded flagellin could activate human immune responses [13]. To the best of our knowledge, an in-depth analysis on the microbial interaction in infantile eczema and corresponding impact on infant health has not been conducted thus far.

In this investigative study, we enrolled 19 infants with eczema and 14 control infants without eczema and compared their GM composition. Furthermore, we analyzed the differential microbial interaction and relative contribution to infant health. The work presented herein may further the understanding of dysbiotic GM and its involvement in eczema progression.

## Methods

### Clinical diagnosis and sample collection

All infants’ parents provided written informed consent, and this study was approved by the Ethical Committee of Shenzhen Children’s Hospital under approval number 2016(004). Eczema was defined as a pruritic, chronic, or chronically relapsing infectious dermatitis with typical features and distribution [14]. All infants with eczema had to satisfy the following inclusion criteria: (1) the patients had to have a clear clinical manifestation of eczema; (2) the patients were not to have other acute and chronic diseases; (3) the skin lesions of patients were not to be infected by bacteria, viruses, or fungi; (4) the patients were required to abstain from ingesting antibiotics, immune modulating inhibitors, or probiotic drinks in the 2 weeks prior to the start of the study; and (5) the patients should not have had diarrhea in the 2 weeks prior to the start of the study. Control subjects (non-eczema infants) were selected from the subjects who passed physical examinations at Shenzhen Children’s Hospital and met the following criteria: (1) all of the infants were not to have allergic diseases or family history of allergic disease; (2) all subjects were required to abstain from ingesting antibiotics, immune modulating inhibitors, or probiotic drinks in the 2 weeks prior to the start of the study; and (3) selected infants should not have diarrhea in the 2 weeks prior to the start of the study.

### DNA extraction, library construction, and sequencing

Microbial DNA was extracted from stool samples using the E.Z.N.A.^®^ Soil DNA Kit (Omega Bio-tek, Norcross, GA, USA) according to the manufacturer’s protocols. Using the PCR kit (TransGenAP221-02), 16S rDNA V4-V5 regions of samples were amplified by primers 515F and 907R. The band widths of PCR products were then verified by 2 % agarose gel electrophoresis, and the quality of PCR products was detected by QuantiFluor™-ST (Promega Corporation). After library construction, the qualified libraries were used for MiSeq sequencing following the Paired-end 300 strategy.

### Taxonomical classification and microbial diversity detection

Produced reads, which contained more than 10 bases with low quality (<Q20) or 15 bp adapter contamination, were removed. Tags were produced by overlapped filtered paired reads and clustered to get operational taxonomic units (OTUs) by USEARCH (v7.0.1090) [15]. OTUs with chimera were removed by UCHIIME (v4.2.40) [16]. Representative OTUs were defined by mapping tags and produced by usearch_global (Additional file 1) [17]. Representative OTUs were aligned with Greengenes database (v201305) [18], and the corresponding taxonomic position was defined by RDP classifier (v2.2) [17] following 0.8 confidence value. The Shannon and Simpson values were calculated by MOTHUR (v1.31.2) [19], which exhibited the richness and evenness of microbial composition.

### Statistical analysis

Χ^2^ test was applied to evaluate the effect, if any, of gender, feeding method, birthing method, and age on GM discrepancy. LEfSe [LDA (Linear discriminant analysis) Effect Size] analysis was used to identify microbial residents enriched in eczema or non-eczema infants, following default standards: the *p* value of the Kruskal–Wallis test and the Wilcoxon test was less than 0.05, and the LDA score was set as 2 [20]. The co-abundance microbial network for eczema subjects and non-eczema counterparts was inferred based on the Pearson index. Pearson’s correlation with coefficient >0.45 or <−0.35 was selected for co-abundance network construction, using Cytoscape software (v2.2.0) [21].

## Results

### Sample collection and data output

The stool samples were collected from randomized 19 eczema and 14 non-eczema infants, all of whom were younger than 6 months old. After 16S rDNA sequencing, the tag number of the stool samples averaged 51,158, with a range of 28,071–66,546. In addition, the overall OTUs number for 33 samples was 269, with 30–93 for non-eczema infants and 33–95 for eczema infants. Evaluations of potential confounders, including gender, feeding method, birthing method, and age, were conducted and all had a minimal impact on the inter-group and the in-group discrepancy (Table 1).

**Table 1:** Characteristics of infants with and without eczema

	Eczema (*n* = 19)	Non-eczema (*n* = 14)	*p* value*
Gender			0.171
Male	15	7	
Female	4	7	
Feeding method			0.305
Breastfeeding	9	10	
Formula feeding	10	4	
Age (months)			0.541
1–3	9	9	
3–6	10	5	
Delivery mode			0.923
Caesarean section	11	7	
Vaginal delivery	8	7	

* *χ* ^2^ test

### Eczema and non-eczema infants showed no significant difference on gut microbial diversity

**Fig. 1:**
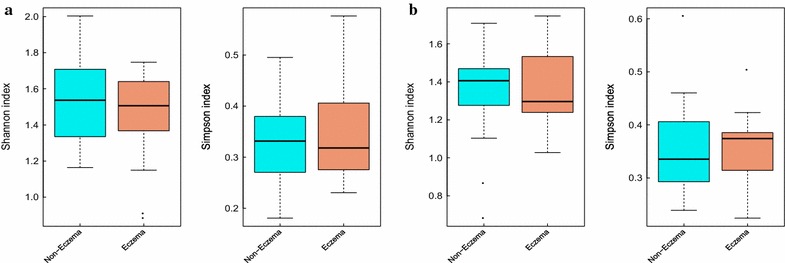
Gut microbial diversity in eczema and non-eczema infants. **a** Distribution of the Shannon and Simpson indices in infants with and without eczema with OTUs results. **b** Gut microbial diversity at the species level. The *cyan* and *red* represent non-eczema and eczema infants, respectively. The gut microbial diversity was slightly low in eczema infants, but there was no significant difference between eczema and non-eczema infants

The Shannon and Simpson indices were applied to evaluate microbial diversity. Based on the OTUs distribution (Additional file 2), the average value of Shannon was 1.528 ± 0.240 (mean ± SD) and 1.440 ± 0.274 in eczema and non-eczema infants, respectively (*p* = 0.482). The Simpson index also reflected little discrepancy: averaging 0.322 ± 0.080 for eczema infants and 0.339 ± 0.096 for non-eczema infants (*p* = 0.871) (Fig. 1). The Shannon and Simpson indices, which were calculated at the genus level, also implicated no apparent differential microbial diversity between eczema and non-eczema infants (0.986 *p* value for the Shannon test and 0.733 *p* value for the Simpson test) (Fig. 1).

### Enriched bacterial residents in eczema and non-eczema infants

**Fig. 2:**
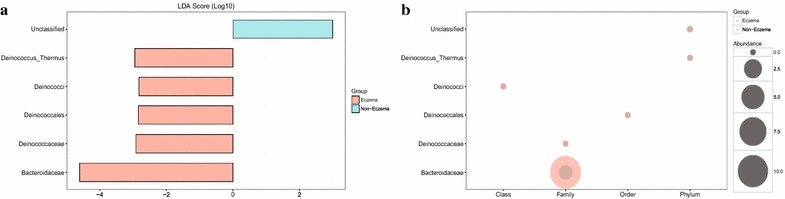
Detection and distribution of biomarkers in eczema and non-eczema infants. **a** Biomarkers discovered by LEfSe analysis and their corresponding LDA score are listed. *Cyan* and *red* represent non-eczema and eczema infants, respectively. **b** Relative abundance of biomarkers in eczema and non-eczema infants. The *cyan* and *red* represent non-eczema and eczema infants, respectively, and the circles with the larger diameter represent the higher abundance. In **b**, it appears that Bacteroidaceae was the primary biomarker responsible for the gut microbial difference between eczema and non-eczema infants

Through LEfSe analysis, a total of 6 differentially enriched bacterial colonizers (5 in the eczema infants and 1 in the non-eczema infants) were identified (Fig. 2). Phylum Deinococcus_Thermus (0.003 ± 0.007 %, LDA score = 2.947), class Deinococci (0.003 ± 0.007 %, LDA score = 2.828), and order Deinococcales (0.002 ± 0.006 %, LDA score = 2.842) were enriched in infants with eczema (Fig. 2). Abundance of family Bacteroidaceae (10.355 ± 18.734 %, LDA score = 4.606) and Deinococcaceae (0.002 ± 0.006 %, LDA score = 2.919) were significantly higher for infants with eczema as compared to the non-eczema samples (Fig. 2). Apparent enrichment of specific bacterial colonizer was not detected at the genus level, partly due to the high similarity of 16S rDNA V4-V5 region for some bacteria, including *Escherichia/Shigella* and *Citrobacter* and *Klebsiella* and *Enterobacter* (Additional file 3).

### Eczema and non-eczema groups harbor distinctive gut microbial co-abundance networks

**Fig. 3:**
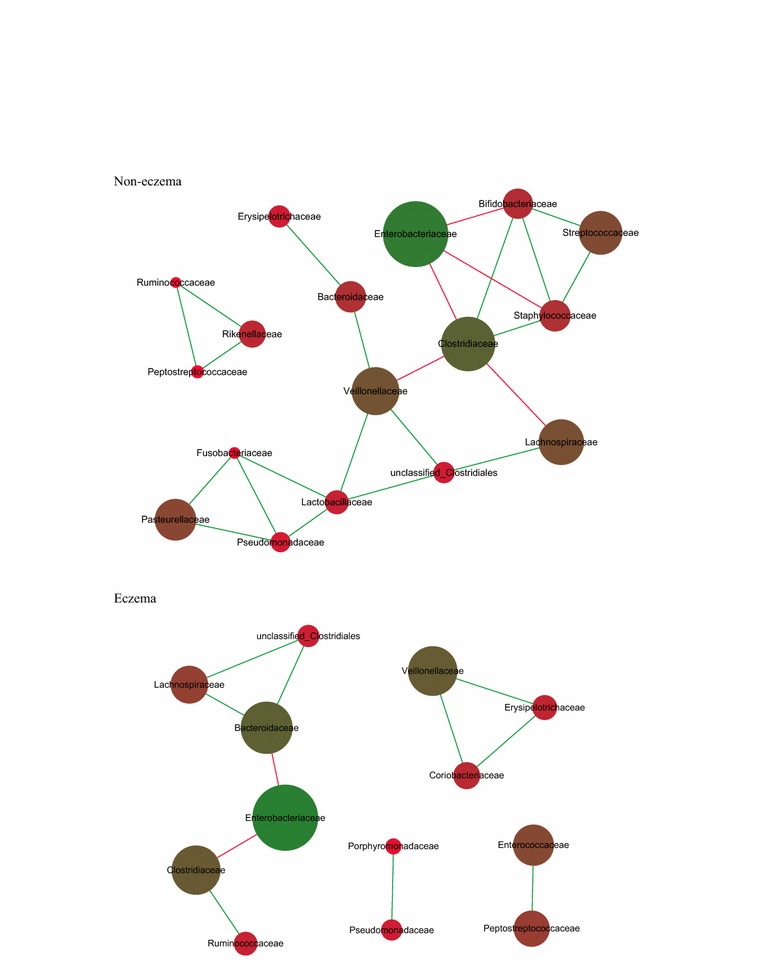
Gut microbial network in eczema and non-eczema infants younger than 6 months. The correlation analysis among gut bacteria was executed and the relationships whose *r* value was higher than 0.45 or lower than −0.35 were kept. The *green* and *red edges* represent the positive correlation and negative correlation, respectively. The diameter of the spots was proportional to the relative abundance. Most of the GM network in the non-eczema group was absent in the group with eczema, but the negative relationship between Enterobacteriaceae and Clostridiaceae remained. Meanwhile direct negative correlation between Bacteroidaceae and Unclassified Clostridiales was enhanced in the non-eczema group

For non-eczema infants, Clostridiaceae, Veillonellaceae, and Lactobacillaceae were the core nodes of the correlation network (Fig. 3). Enriched Enterobacteriaceae was negatively correlated with Clostridiaceae (*r* = −0.459), while Veillonellaceae was positively correlated with Bacteroidaceae (*r* = 0.494) and Unclassified Clostridiales (*r* = 0.485). The aforementioned networks seemed to be absent in eczema infants: Veillonellaceae was involved in a different correlation independently, and the direct correlation was found between Bacteroidaceae and Enterobacteriaceae (*r* = −0.570) and Bacteroidaceae and Unclassified Clostridiales (*r* = 0.800) (Fig. 3). The negative correlation between Clostridiaceae and Enterobacteriaceae had a tendency to be similar in both the eczema (*r* = 0 to 0.452) and the non-eczema infants (*r* = −0.459) (Fig. 3).

## Discussion

This study mainly focused on the GM discrepancy between eczema and non-eczema infants under 6 months of age in China. The results suggested a slightly lower microbial diversity in eczema infants, which differed from a prior report [3]. This was possibly attributed to the relative abundance discrepancy of GM between eczema and non-eczema infants, and immature GM showed no significant change.

At the genus level, it was difficult to distinguish *Escherchia/Shigella* from *Citrobacter* and *Klebsiella* from *Enterobacter* based on 16S rDNA V4-V5 region (Additional file 3). The confounded microbial composition analysis at the genus level was due to a high proportion of *Escherchia/Shigella*, *Citrobacter*, *Klebsiella*, and *Enterobacter*. At the family level, Bacteroidaceae and Deinococcaceae were more abundant in eczema infants. Prior reports demonstrated that increasing *Bacteroides*, which were classified into the family Bacteroidaceae, could promote the secretion of IL-6 and IL-23 in dendritic cells [13]. IL-6 and IL-23 are known to promote the differentiation of Th17 cells; the secretion of IL-17 by Th17 cells could trigger an inflammatory response and autoimmune diseases [13, 22]. Therefore, the association between increased Bacteroidaceae and eczema could be partly attributed to *Bacteroides*-related cytokines. The function of Deinococcaceae, which exhibited radio resistance and is normally found in soil [23], has seldom been reported in the human gut.

Another finding in this study was the distinctive GM co-abundance network between the two groups. For non-eczema subjects, Clostridiaceae, Veillonellaceae, and Lactobacillaceae correlated with other microbial colonizers. In eczema infants, the overall microbial linkage observed in non-eczema subjects was absent, and instead featured the core-node of Enterobacteriaceae, which included opportunistic pathogens such as *Klebsiella, Salmonella,* and *Shigella*. This may result in intense feedback from the host immune system. Combined with enriched Bacteroidaceae, which was previously documented to promote cytokines secretion [13, 22], eczema infants may have a hyperactive immune system.

## Conclusions

Although this study furthers the understanding of GM involvement in infantile eczema development, there are some issues that still need to be resolved: detection of microbial interaction and discrepancy at lower taxonomic levels; large cohort studies to confirm these findings; and experimental studies to confirm the identified contributors. In summary, this work lays the foundation for a theoretical method of GM involvement in eczema development.

## Additional files

10.1186/s13099-016-0118-0 The representative sequences of OTUs in 33 infants.
10.1186/s13099-016-0118-0 The distribution of OTUs in 33 infants.
10.1186/s13099-016-0118-0 Basic Local Alignment Search Tool (BLAST) results of selected OTUs. The sequence of OTU1 and OTU2 were put into BLAST on the NCBI website. In (A), the sequence of OTU2 had 1 mismatch with *Klebsiella quasipneumoniae* and *Enterobacter cloacae*. In (B), OTU1 had 1 mismatch with *Shigella sonnei* and 3 mismatches with *Cronobacter turicensis.* Due to the high similarity between their V4-V5 regions, genus *Klebsiella* and *Enterobacter* could not be distinguished, as was similar for *Shigella* and *Cronobacter.*

## Abbreviations

DC: Dendritic cells
GM: Gut microbiota
IL-17: Interleukin-17
IL-6: Interleukin-6
IL-23: Interleukin-23
LDA: Linear discriminant analysis
LEfSe: LDA Effect Size
OTUs: Operational taxonomic units
Th17: T helper cell 17
