# Supplementary material for: Dysfunctional gut microbiota and relative co-abundance network in infantile eczema
Source: Gut Pathog. 2016 Jul 22;8:36. doi: 10.1186/s13099-016-0118-0 (PMC4957860; doi:10.1186/s13099-016-0118-0)
Supplement: Supplementary file 1 — 10.1186/s13099-016-0118-0 The representative sequences of OTUs in 33 infants. [file 13099_2016_118_MOESM1_ESM.docx]

>OTU1

TAATACGGAGGGTGCAAGCGTTAATCGGAATTACTGGGCGTAAAGCGCACGCAGGCGGTTTGTTAAGTCAGATGTGAAATCCCCGGGCTCAACCTGGGAACTGCATCTGATACTGGCAAGCTTGAGTCTCGTAGAGGggggTAGAATTCCAGGTGTAGCGGTGAAATGCGTAGAGATCTGGAGGAATACCGGTGGCGAAGGCGGCCcccTGGACGAAGACTGACGCTCAGGTGCGAAAGCGTGGGGAGCAAACAGGATTAGATACCCTGGTAGTCCACGCCGTAAACGATGTCGACTTGGAGGTTGTGCCCTTGAGGCGTGGCTTCCGGAGCTAACGCGTTAAGTCGACCGCCTGGGGAGTACGGCCGCAAGGTTAAAACTTAAATGAATTGACGG

>OTU2

TAATACGGAGGGTGCAAGCGTTAATCGGAATTACTGGGCGTAAAGCGCACGCAGGCGGTCTGTCAAGTCGGATGTGAAATCCCCGGGCTCAACCTGGGAACTGCATTCGAAACTGGCAGGCTAGAGTCTTGTAGAGGggggTAGAATTCCAGGTGTAGCGGTGAAATGCGTAGAGATCTGGAGGAATACCGGTGGCGAAGGCGGCCcccTGGACAAAGACTGACGCTCAGGTGCGAAAGCGTGGGGAGCAAACAGGATTAGATACCCTGGTAGTCCACGCCGTAAACGATGTCGATTTGGAGGTTGTGCCCTTGAGGCGTGGCTTCCGGAGCTAACGCGTTAAATCGACCGCCTGGGGAGTACGGCCGCAAGGTTAAAACTTAAATGAATTGACGG

>OTU3

TAATACGTAGGTGGCAAGCGTTGTCCGGAATTATTGGGCGTAAAGCgcgcgcAGGCGGCCTTTTAAGTCTGATGTGAAAGCCcccGGCTCAACCGGGGAGGGCCATTGGAAACTGGAAGGCTTGAGTACAGAAGAGAAGAGTGGAATTCCACGTGTAGCGGTGAAATGCGTAGAGATGTGGAGGAACACCAGTGGCGAAGGCGACTCTTTGGTCTGTAACTGACGCTGAGGCGCGAAAGCGTGGGGAGCAAACAGGATTAGATACCCTGGTAGTCCACGCCGTAAACGATGAGTGCTAGGTGTTGGggggTTTCCGCCCCTCAGTGCTGAAGCTAACGCATTAAGCACTCCGCCTGGGGAGTACGGCCGCAAGGCTGAAACTTAAAGGAATTGACGG

>OTU4

TAATACGTAGGgggCAAGCGTTATCCGGATTTACTGGGTGTAAAGGGAGCGTAGACGGCGAAGCAAGTCTGAAGTGAAAACCCAGGGCTCAACCCTGGGACTGCTTTGGAAACTGTTTTGCTAGAGTGTCGGAGAGGTAAGTGGAATTCCTAGTGTAGCGGTGAAATGCGTAGATATTAGGAGGAACACCAGTGGCGAAGGCGGCTTACTGGACGATAACTGACGTTGAGGCTCGAAAGCGTGGGGAGCAAACAGGATTAGATACCCTGGTAGTCCACGCCGTAAACGATGAATGCTAGGTGTTGGggggCAAAGCCCTTCGGTGCCGTCGCAAACGCAGTAAGCATTCCACCTGGGGAGTACGTTCGCAAGAATGAAACTTAAAGGAATTGACGGC

>OTU5

TAATACGGAGGATGCGAGCGTTATCCGGATTTATTGGGTTTAAAGGGTGCGTAGGCGGCCTGTTAAGTCAGCGGTGAAATCTAGGAGCTTAACTCCTAAATTGCCATTGATACTGGCGGGCTTGAGTGTAGATGAGGTAGGCGGAATGCGTGGTGTAGCGGTGGAATGCATAGATATCACGCAGAACTCCGATTGCGAAGGCAGCTTACTAAGGTACAACTGACGCTGAAGCACGAAAGCGTGGGTATCAAACAGGATTAGATACCCTGGTAGTCCACGCAGTAAACGATGATAACTGGGCGTATGCGATATACAGTATGCTCCTAAGCGAAAGCGTTAAGTTATCCACCTGGGGAGTACGCCGGCAACGGTGAAACTTAAATGAATTGACGG

>OTU6

TAATACGTAGGCGGCAAGCGTTGTCCGGAATTATTGGGCGTAAAGGGAGCGCAGGCGGGAAACTAAGCGGATCTTAAAAGTGCGGGGCTCAACCCCGTGATGGGGTCCGAACTGGTTTTCTTGAGTGCAGGAGAGGAAAGCGGAATTCCCAGTGTAGCGGTGAAATGCGTAGATATTGGGAAGAACACCAGTGGCGAAGGCGGCTTTCTGGACTGTAACTGACGCTGAGGCTCGAAAGCTAGGGTAGCGAACGGGATTAGATACCCCGGTAGTCCTAGCCGTAAACGATGGATACTAGGTGTGGGAGGTATCGACCCCTTCCGTGCCGGAGTTAACGCAATAAGTATCCCGCCTGGGGAGTACGGCCGCAAGGTTGAAACTTAAATGAATTGACGG

>OTU7

TAATACGGAGGGTGCAAGCGTTAATCGGAATTACTGGGCGTAAAGCGTgtgtAGGCGGTTTGTTAAGTCTGTTGTGAAAGCCCCGGGCTCAACCTGGGAATGGCAATGGATACTGGCAAGCTAGAGTGTGATAGAGGATGGTGGAATTCCCGGTGTAGCGGTGAAATGCGTAGAGATCGGGAGGAACATCAGTGGCGAAGGCGGCCATCTGGATCAACACTGACGCTGAGACACGAAAGCGTGGGGAGCAAACAGGATTAGATACCCTGGTAGTCCACGCCCTAAACGATGCGAACTGGATGTTGGTCTCAACTCGGAGATCAGTGTCGAAGCTAACGCGTTAAGTTCGCCGCCTGGGGAGTACGGTCGCAAGACTGAAACTTAAAGGAATTGACGG

>OTU8

TCACACGATTAACCCAAGTCAATAGAAGCCGGCGTAAAGAGTGTTTTAGATCACCcccTCCCCAATAAAGCTAAAACTCACCTGAGTTGTAAaaaaCTCCAGTTGACACAAAATAGACTACGAAAGTGGCTTTAACATATCTGAACacacaATAGCTAAGACCCAAACTGGGATTAGATACCCCACTATGCTTAGCCCTAAACCTCAACAGTTAAATCAACAAAACTGCTCGCCAGAACACTACGAGCCACAGCTTAAAACTCAAATGAATTGACGG

>OTU9

TAATACGTAGGTGGCAAGCGTTGTCCGGATTTATTGGGCGTAAAGCGAGCGCAGGCGGAATGATAAGTCTGATGTGAAAGCCCACGGCTCAACCGTGGAACTGCATCGGAAACTGTCATTCTTGAGTGCAGAAGAGGAGAGTGGAACTCCATGTGTAGCGGTGGAATGCGTAGATatatGGAAGAACACCAGTGGCGAAGGCGGCTctctGGTCTGCAACTGACGCTGAGGCTCGAAAGCATGGGTAGCGAACAGGATTAGATACCCTGGTAGTCCATGCCGTAAACGATGAGCGCTAGGTGTTGGGGACTTTCCGGTCCTCAGTGCCGCAGCAAACGCATTAAGCGCTCCGCCTGGGGAGTACGACCGCAAGGTTGAAACTCAAATGAATTGACGG

>OTU10

TAATACGGAGGGTGCAAGCGTTAATCGGAATTACTGGGCGTAAAGCGCACGCAGGCGGTCTGTCAAGTCGGATGTGAAATCCCCGGGCTCAACCTGGGAACTGCATTCGAAACTGGCAGGCTAGAGTCTTGTAGAGGggggTAGAATTCCAGGTGTAGCGGTGAAATGCGTAGATATTAGGAGGAACACCAGTGGCGAAGGCGGCTTACTGGACGATAACTGACGTTGAGGCTCGAAAGCGTGGGGAGCAAACAGGATTAGATACCCTGGTAGTCCACGCCGTAAACGATGTCGATTTGGAGGTTGTGCCCTTGAGGCGTGGCTTCCGGAGCTAACGCGTTAAATCGACCGCCTGGGGAGTACGGCCGCAAGGTTAAAACTTAAAGGAATTGACGG

>OTU11

TAAAACGTAGGAGGCAAGCGTTATCCGGATTTACTGGGCGTAAAGGGCGTGCAGGTGGCTGAATAAGTGGTGCGTGAAAGCGCTCGGCTCAACCGGGCGAGGGCGTGCCAAACTGTTCAACTAGAAACAGGCAGAGGAAAGTGGAATTCGGGGTGTAGTGGTGAAATGCGTAGAGATCCCGAGGAACTCCTGTGGCGAAGGCGACTTTCTGGGCCTGCTTTGACACTCAGACGCGAAAGCATGGGGAGCGAACGGGATTAGAAACCCCGGTAGTCCATGCCGTAAACGATGTCCACTGGGCGTTTGCCTCGCAAGAGGTGAGTGCCGCAGCCAACGCGCTAAGTGGACCGCCTGGGGAGTACGACCGCAAGGTTAAAACTCAAAGGAATTGACGG

>OTU12

TAATACGGAGGATCCGAGCGTTATCCGGATTTATTGGGTTTAAAGGGAGCGTAGGCGGACTATTAAGTCAGCTGTGAAAGTTTGCGGCTCAACCGTAAAATTGCAGTTGATACTGGTCGTCTTGAGTGCAGTAGAGGTAGGCGGAATTCGTGGTGTAGCGGTGAAATGCTTAGATATCACGAAGAACTCCGATTGCGAAGGCAGCTTACTGGACTGTAACTGACGCTGATGCTCGAAAGTGTGGGTATCAAACAGGATTAGATACCCTGGTAGTCCAcacaGTAAACGATGAATACTCGCTGTTTGCGATATACAGCAAGCGGCCAAGCGAAAGCATTAAGTATTCCACCTGGGGAGTACGCCGGCAACGGTGAAACTTAAATGAATTGACGG

>OTU13

TAATACGTATGGAGCGAGCGTTGTCCGGAATTATTGGGCGTAAAGGGTGCGCAGGCGGCTTTACAAGTTGGATGTGAAATATTGTGGCTCAACCACAAACGTGCATCCAAAACTGCAAAGCTTGAGTTAAGGAGAGGTAAGTGGAATTCCTGGTGTAGCGGTGGAATGCGTAGATATCAGGAGGAATACCGGTGGCGAAGGCGACTTACTGGACTTAAACTGACGCTCAGGCACGAAAGCGTGGGGAGCAAACAGGATTAGATACCCTGGTAGTCCACGCCGTAAACGATGAGTGCTAGGTGTTGGgggTCAAACCTCGGTGCCGCAAGCAAACGCAATAAGCACTCCGCCTGGGGAGTACGTGCACAAGCATGAAACTTAAATGAATTGACGG

>OTU14

TAATACGGAGGGTGCAAGCGTTAATCGGAATTACTGGGCGTAAAGCGAGCGCAGGCGGTTTGATAAGTCTGAAGTTAAAGGCTGTGGCTCAACCATAGTTCGCTTTGGAAACTGTCAAACTTGAGTGCAGAAGGGGAGAGTGGAATTCCATGTGTAGCGGTGAAATGCGTAGATatatGGAGGAACACCGGTGGCGAAAGCGGCTctctGGTCTGTAACTGACGCTGAGGCTCGAAAGCGTGGGGAGCGAACAGGATTAGATACCCTGGTAGTCCACGCCGTAAACGATGAGTGCTAGGTGTTGGGTCCTTTCCGGGATTCAGTGCCGCAGCTAACGCATTAAGCACTCCGCCTGGGGAGTACGACCGCAAGGTTGAAACTCAAATGAATTGACGG

>OTU15

TAATACGTAGGTGGCAAGCGTTATCCGGAATTATTGGGCGTAAAGAGGGAGCAGGCGGCAGCAAGGGTCTGTGGTGAAAGCCTGAAGCTTAACTTCAGTAAGCCATAGAAACCAGGCAGCTAGAGTGCAGGAGAGGATCGTGGAATTCCATGTGTAGCGGTGAAATGCGTAGATatatGGAGGAACACCAGTGGCGAAGGCGACGATCTGGCCTGCAACTGACGCTCAGTCCCGAAAGCGTGGGGAGCAAATAGGATTAGATACCCTAGTAGTCCACGCCGTAAACGATGAGTACTAAGTGTTGGATGTCAAAGTTCAGTGCTGCAGTTAACGCAATAAGTACTCCGCCTGAGTAGTACGTTCGCAAGAATGAAACTTAAATGAATTGACGGT

>OTU16

TAACACGTAGGATTCAAACGTTATCCGGATTTACTGGGCGTAAAGCGCGTGCAGGCGGTTTGGTAAGTTGGATGTGAAAGCTCCGGGCTCAACCCGGAGAGGTCGTTCAATACTGCCAGACTAGAGGACGATAGAGGAAGGTGGAATTCCAGGTGTAGTGGTGAAATGCGTAGATATCTGGAGGAACACCAGTGGCGAAAGCGGCCTTCTGGGTCGGTCCTGACGCTAAGACGCGAAAGCTAGGGTAGCAAACGGGATTAGAAACCCCGGTAGTCCTAGCCGTAAACGATGTGAACTTGGTGTTGGTGGGGTAAAAGCCATCAGTGCCGTAGCTAACGCGATAAGTTCACCGCCTGGGGACTACGGTCGCAAGATTAAAACTCAAAGGAATTGACGG

>OTU17

TAATACGTAGGTCCCGAGCGTTGTCCGGATTTATTGGGCGTAAAGCGAGCGCAGGCGGTTAGAAAAGTCTGAAGTGAAAGGCAGTGGCTCAACCATTGTAGGCTTTGGAAACTGTTTAACTTGAGTGCAGAAGGGGAGAGTGGAATTCCATGTGTAGCGGTGAAATGCGTAGATatatGGAGGAACACCGGTGGCGAAAGCGGCTctctGGTCTGTAACTGACGCTGAGGCTCGAAAGCGTGGGGAGCGAACAGGATTAGATACCCTGGTAGTCCACGCCGTAAACGATGAGTGCTAGGTGTTAGGTCCTTTCCGGGACTTAGTGCCGCAGCTAACGCATTAAGCACTCCGCCTGGGGAGTACGACCGCAAGGTTGAAACTTAAATGAATTGACGG

>OTU18

TAATACGTAGGGAGCAAGCGTTATCCGGATTTACTGGGTGTAAAGGGCGTGTAGGCGGGCTTGTAAGTTGGAAGTGAAATCTCGGGGCTTAACCCCGAAACTGCTTTCAAAACTGCGAGTCTTGAGTGATGGAGAGGCAGGCGGAATTCCCAGTGTAGCGGTGAAATGCGTAGATATTGGGAGGAACACCAGTGGCGAAGGCGGCCTGCTGGACATTAACTGACGCTGAGGCGCGAAAGCGTGGGGAGCAAACAGGATTAGATACCCTGGTAGTCCACGCCGTAAACGATGGATACTAGGTGTGGGAGGTATTGACCCCTTCCGTGCCGGAGTTAACACAATAAGTATCCCACCTGGGGAGTACGGCCGCAAGGTTGAAACTTAAATGAATTGACGG

>OTU19

TAAGACGGggggggCAAGTGTTCTTCGGAATGACTAGGCGTAAAGGGCACGTAGGCGGTGAATCGGGTTGAAAGTGAAAGTCGCCAAaaaGTGGCGGAATGCTCTCGAAACCAATTCACTTGAGTGAGACAGAGGAGAGTGGAATTTCGTgtgtAGGGGTGAAATCCGTAGATCTACGAAGGAACGCCAAAAGCGAAGGCAGCTctctGGGTCCCTACCGACGCTGGGGTGCGAAAGCATGGGGAGCGAACAGGATTAGATACCCTGGTAGTCCATGCCGTAAACGATGAGTGTTCGCCCTTGGTCTACGCGGATCAGGGGCCCAGCTAACGCGTGAAACACTCCGCCTGGGGAGTACGGTCGCAAGACCGAAACTCAAAGGAATTGACGG

>OTU20

TAATACGGAGGGTGCGAGCGTTATCCGGAATCACTGGGCGTAAAGGGCGTGTAGGCGGGACGTTAAGTCTGGTTTTAAAGACCGCAGCTCAACTGCGGGAGTGGACTGGATACTGGCGTTCTAGACCTCTGGAGAGGTAACTGGAATTTCTGGTGTAGCGGTGGAATGCGTAGATACCAGAAGGAACACCAATGGCGAAGGCAGGTTACTGGACAGAAGGTGACGCTGAGGCGCGAAAGTGTGGGGAGCAAACCGGATTAGATACCCGGGTAGTCCACACCCTAAACGATGTACGTTGGTCTAGCGCAGGATGCTGTGTTGGACGAAGCTAACGCGATAAACGTACCGCCTGGGAAGTACGGCCGCAAGGTTGAAACTCAAAGGAATTGACGG

>OTU21

TAATACGGAGGATGCGAGCGTTATCCGGATTTATTGGGTTTAAAGGGAGCGCAGACGGGAGATTAAGTCAGTTGTGAAAGTTTGCGGCTCAACCGTAAAATTGCAGTTGATACTGGTTTCCTTGAGTGCAGTTGAGGCAGGCGGAATTCGTGGTGTAGCGGTGAAATGCTTAGATATCACGAAGAACCCCGATTGCGAAGGCAGCTTGCTAAACTGTAACTGACGTTCATGCTCGAAAGTGTGGGTATCAAACAGGATTAGATACCCTGGTAGTCCACACGGTAAACGATGGATACTCGCTGTTGGCGATATACTGTCAGCGGCCAAGCGAAAGCATTAAGTATCCCACCTGGGGAGTACGCCGGCAACGGTGAAACTTAAATGAATTGACGG

>OTU22

TAATACGTAGGGCGCAAGCGTTGTCCGGAATTATTGGGCGTAAAGAGCTCGTAGGTGGCTTGTCGCGTCTGCCGTGAAAACCCGAGGCTCAACCTCGGGCGTGCGGTGGGTACGGGCAGGCTGGAGTGTGGTAGGGGAGACTGGAACTCCTGGTGTAGCGGTGAAATGCGCAGATATCAGGAAGAACACCGATGGCGAAGGCAGGTCTCTGGGCCATTACTGACACTGAGGAGCGAAAGCATGGGTAGCGAACAGGATTAGATACCCTGGTAGTCCATGCCGTAAACGTTGGGCACTAGATGTGGgggACATTCCACGTTTTCCGCGTCGTAGCTAACGCATTAAGTGCCCCGCCTGGGGAGTACGGCCGCAAGGCTAAAACTCAAAGGAATTGACGG

>OTU23

TAATACGTAAGGTCCGAGCGTTGTCCGGAATCATTGGGCGTAAAGGGTACGTAGGCGGGTAAGCAAGTTAGAAGTGAAATCCTATAGCTCAACTATAGTAAGCTTTTAAAACTGCTCATCTTGAGGTATGGAAGGGAAAGTGGAATTCCTAGTGTAGCGGTGAAATGCGCAGATATTAGGAGGAATACCGGTGGCGAAGGCGACTTTCTGGCCATAACCTGACGCTGAGGTACGAAAGCGTGGGTAGCAAACAGGATTAGATACCCTGGTAGTCCACGCCGTAAACGATGAGTGTTAGGTGTCTGGAGTAAATCTGGGTGCCGCAGCTAACGCATTAAACACTCCGCCTGGGGAGTACGCACGCAAGTGTGAAACTCAAAGGAATTGACGG

>OTU24

TAATACGTAGGGAGCAAGCGTTATCCGGATTTACTGGGTGTAAAGGGCgcgcAGGCGGGCTGGCAAGTTGGGAGTGAAATGCCGGGGCTTAACCCCGGAACTGCTTTCAAAACTGCTGGTCTTGAGTGATGGAGAGGCAGGCGGAATTCCGTgtgtAGCGGTGAAATGCGTAGATATACGGAGGAACACCAGTGGCGAAGGCGGCCTGCTGGACATTAACTGACGCTGAGGCGCGAAAGCGTGGGGAGCAAACAGGATTAGATACCCTGGTAGTCCACGCCGTAAACGATGGATACTAGGTGTGGGAGGTATTGACCCCTTCCGTGCCGCAGTTAACACAATAAGTATCCCACCTGGGGAGTACGGCCGCAAGGTTGAAACTCAAATGAATTGACGG

>OTU25

TAATACGAAGGgggCTAGCGTTGCTCGGAATCACTGGGCGTAAAGGGCGCGTAGGCGGTCTTTTAAGTCGGgggTGAAAGCCTGTGGCTCAACCACAGAATTGCCTTCGATACTGGGAGACTTGAGTTCGGAAGAGGTTGGTGGAACTGCGAGTGTAGAGGTGAAATTCGTAGATATTCGCAAGAACACCGGTGGCGAAGGCGGCCAACTGGTCCGACACTGACGCTGAGGCGCGAAAGCGTGGGGAGCAAACAGGATTAGATACCCTGGTAGTCCACGCCGTAAACGATGAATGCCAGCCGTTGGggggCTTGCCTTTCAGTGGCGCAGCTAACGCTTTGAGCATTCCGCCTGGGGAGTACGGTCGCAAGATTAAAACTTAAATGAATTGACGG

>OTU26

TAATACGGAAGGTCCGGGCGTTATCCGGATTTATTGGGTTTAAAGGGAGCGTAGGCCGGAGATTAAGCGTGTTGTGAAATGTAGACGCTCAACGTCTGCACTGCAGCGCGAACTGGTTTCCTTGAGTACGCACAAAGTGGGCGGAATTCGTGGTGTAGCGGTGAAATGCTTAGATATCACGAAGAACTCCGATTGCGAAGGCAGCTCACTGGAGCGCAACTGACGCTGAAGCTCGAAAGTGCGGGTATCGAACAGGATTAGATACCCTGGTAGTCCGCACGGTAAACGATGGATGCCCGCTGTTGGTCTGAATAGGTCAGCGGCCAAGCGAAAGCATTAAGCATCCCACCTGGGGAGTACGCCGGCAACGGTGAAACTTAAATGAATTGACGG

>OTU27

TAATACGTAGGTGGCAAGCGTTATCCGGAATTATTGGGCGTAAAGCgcgcgTAGGCGGTTttttAAGTCTGATGTGAAAGCCCACGGCTCAACCGTGGAGGGTCATTGGAAACTGGAAAACTTGAGTGCAGAAGAGGAAAGTGGAATTCCATGTGTAGCGGTGAAATGCGCAGAGATATGGAGGAACACCAGTGGCGAAGGCGACTTTCTGGTCTGTAACTGACGCTGATGTGCGAAAGCGTGGGGATCAAACAGGATTAGATACCCTGGTAGTCCACGCCGTAAACGATGAGTGCTAAGTGTTAGGgggTTTCCGCCCCTTAGTGCTGCAGCTAACGCATTAAGCACTCCGCCTGGGGAGTACGACCGCAAGGTTGAAACTCAAAGGAATTGACGGG

>OTU28

TAATACCAGCACCCCGAGTGGTCGGGACGATTATTGGGCCTAAAGCATCCGTAGCCGGTTTTACAAGTCCTCCGTTAAATCCAACTGCTTAACAGATGGGCCGCGGAGGATACTATAAGACTAGGAGGCAGGAGAGGCAAGCGGTACTCAGTGGGTAGGGGTAAAATCCGTTGATCCATTGAAGACCACCAGTGGCGAAGGCGGCTTGCCAGAATGCGCTCGACGGTGAGGGATGAAAGCTGGgggAGCAAACCGGATTAGATACCCGGGTAGTCCCAGCTGTAAACGATGCAGACTCGGTGATGGACTAGCTTCATGCTAGTCCAGTGCCGCAGGGAAGCCGTTAAGTCTGCCGCCTGGGTAGTACGGTCGCAAGACTGAAACTTAAATGAATTGACGG

>OTU29

TGATACGTAGGGTGCGAGCGTTGTCCGGATTTATTGGGCGTAAAGGGCTCGTAGGTGGTTGATCGCGTCGGAAGTGTAATCTTGGGGCTTAACCCTGAGCGTGCTTTCGATACGGGTTGACTTGAGGAAGGTAGGGGAGAATGGAATTCCTGGTGGAGCGGTGGAATGCGCAGATATCAGGAGGAACACCAGTGGCGAAGGCGGTTCTCTGGGCCTTTCCTGACGCTGAGGAGCGAAAGCGTGGGGAGCGAACAGGCTTAGATACCCTGGTAGTCCACGCTGTAAACGGTGGGTACTAGGTGTGGGGTCCATTCCACGGGTTCCGTGCCGTAGCTAACGCTTTAAGTACCCCGCCTGGGGAGTACGGCCGCAAGGCTAAAACTTAAATGAATTGACGG

>OTU30

CGAAAGGTAACTGGGTGGTGATTGACGGCGGTAGCgcgcCGGAAAACGACATCGTCGCCATCCGCGAATTGAGTCCGACACGGCTGCTGATTGTCGACGCCACGGATATGGGGCTAAACCCCGGCGAGATCCGCATTATCGACCCGGATGACATCGCCGAGATGTTTATGATGACTACCCATAACATGCCGTTGAACTACCTTATCGACCAGTTGAAAGAAGACGTTGGTGAAGTGATTTTCCTCGGCATTCAGCCGGATATCGTCGGCTTTTACTACCCGATGACTCAGCCGGTTAAAGATGCGGTGGAAACGGTGTATCAGCGGCTGGAAGGCTGGGAAGGAAATGGCGGCTTCGCGCAGTTAGCGGTGGAAGAAGAGTAGTTCTTCGTTAAGGAATTGACGG

>OTU31

TAATACGTAGGGTGCGAGCGTTGTCCGGAATTATTGGGCGTAAAGAGCTTGTAGGCGGCTTGTCGCGTCTGCTGTGAAAGCCCGGGGCTTAACTCCGGGTCTGCAGTGGGTACGGGCAGGCTAGAGTGTGGTAGGGGAGACTGGAATTCCTGGTGTAGCGGTGGAATGCGCAGATATCAGGAGGAACACCGATGGCGAAGGCAGGTCTCTGGGCCATTACTGACGCTGAGAAGCGAAAGCGTGGGGAGCGAACAGGATTAGATACCCTGGTAGTCCACGCCGTAAACGTTGGGCGCTAGGTGTGGGTCCCATTCCACGGGGTCCGTGCCGCAGCTAACGCATTAAGCGCCCCGCCTGGGGAGTACGGCCGCAAGGCTAAAACTTAAATGAATTGACGG

>OTU32

TAATACGGAGGGTGCAAGCGTTATCCGGATTCACTGGGTTTAAAGGGTGCGTAGGTGGGTTGGTAAGTCAGTGGTGAAATCTCCGAGCTTAACTTGGAAACTGCCATTGATACTATCAGTCTTGAATATTGTGGAGGTTAGCGGAATATGTCATGTAGCGGTGAAATGCTTAGATATGACATAGAACACCAATTGCGAAGGCAGCTGGCTACACATatatTGACACTGAGGCACGAAAGCGTGGGGATCAAACAGGATTAGATACCCTGGTAGTCCACGCCCTAAACTATGGATACTCGACATACGCGATAAACTGtgtgtgtCTGAGCGAAAGCATTAAGTATCCCACCTGGGAAGTACGATCGCAAGATTGAAACTCAAATGAATTGACGG

>OTU33

TAATACGTAGGTGGCAAGCGTTGTCCGGAATTATTGGGCGTAAAGAGCATGTAGGCGGGCTTTTAAGTCCGACGTGAAAATGCGGGGCTTAACCCCGTATGGCGTTGGATACTGGAAGTCTTGAGTGCAGGAGAGGAAAGGGGAATTCCCAGTGTAGCGGTGAAATGCGTAGATATTGGGAGGAACACCAGTGGCGAAGGCGCCTTTCTGGACTGTGTCTGACGCTGAGATGCGAAAGCCAGGGTAGCAAACGGGATTAGATACCCCGGTAGTCCTGGCCGTAAACGATGGGTACTAGGTGTAGGAGGTATCGACCCCTTCTGTGCCGGAGTTAACGCAATAAGTACCCCGCCTGGGGACTACGATCGCAAGATTGAAACTTAAAGGAATTGACGG

>OTU34

TAATACGTAGGGAGCAAGCGTTGTCCGGATTTACTGGGTGTAAAGGGTGCGTAGGCGGTTTGGTAAGTCAGAAGTGAAATCCATGGGCTTAACCCATGAACTGCTTTTGAAACTATCGAACTTGAGTGAAGTAGAGGTAGGCGGAATTCCCGGTGTAGCGGTGAAATGCGTAGAGATCGGGAGGAACACCAGTGGCGAAGGCGGCCTACTGGGCTTTAACTGACGCTGAGGCACGAAAGCATGGGTAGCAAACAGGATTAGATACCCTGGTAGTCCATGCCGTAAACGATGATTACTAGGTGTGGggggTCTGACCcccTCCGTGCCGGAGTTAACACAATAAGTAATCCACCTGGGGAGTACGACCGCAAGGTTGAAACTCAAAGGAATTGACGG

>OTU35

TAATACGTAGGgggCTAGCGTTGTCCGGAATCACTGGGCGTAAAGGGTTCGCAGGCGGAAATGCAAGTCAGGTGTAAAAGGCAGTAGCTTAACTACTGTAAGCATTTGAAACTGCATATCTTGAGAAGAGTAGAGGTAAGTGGAATTtttAGTGTAGCGGTGAAATGCGTAGATATTAAaaaGAATACCGGTGGCGAAGGCGACTTACTGGGCTCATTCTGACGCTGAGGAACGAAAGCGTGGGTAGCAAACAGGATTAGATACCCTGGTAGTCCACGCTGTAAACGATGAGTGCTAGGTGTCGGCGTAAGTCGGTGCCGCAGTTAACACAATAAGCACTCCGCCTGGGGAGTACGTGCGCAAGCATGAAACTTAAATGAATTGACGG

>OTU36

TAATACGGAGGGTGCAAGCGTTATCCGGATTCACTGGGTTTAAAGGGTGCGTAGGTGGGTTGGTAAGTCAGTGGTGAAATCTCCGGGCTTAACTCGGAAACTGCCGTTGATACTATCAATCTTGAATATTGTGGAGGTAAGCGGAATATGTCATGTAGCGGTGAAATGCTTAGATATGACATAGAACACCGATAGCGAAGGCAGCTTGCTACACAATTATTGACACTGAGGCACGAAAGCGTGGGGATCAAACAGGATTAGATACCCTGGTAGTCCACGCCCTAAACGATGGATACTCGACATACGCGATACACAGTgtgtgtCTGAGCGAAAGCATTAAGTATCCCACCTGGGAAGTACGACCGCAAGGTTGAAACTCAAAGGAATTGACGG

>OTU37

TAATACGTAGGGTGCGAGCGTTAATCGGAATTACTGGGCGTAAAGCGTGCGCAGGCGGCTTTGCAAGACAGATGTGAAATCCCCGGGCTCAACCTGGGAACTGCATTTGTGACTGCAAGGCTAGAGTACGGCAGAGGgggATGGAATTCCGCGTGTAGCAGTGAAATGCGTAGATATGCGGAGGAACACCGATGGCGAAGGCAATCCCCTGGGCCTGTACTGACGCTCATGCACGAAAGCGTGGGGAGCAAACAGGATTAGATACCCTGGTAGTCCACGCCCTAAACGATGTCAACTGGTTGTTGGACGGCTTGCTGTTCAGTAACGAAGCTAACGCGTGAAGTTGACCGCCTGGGGAGTACGGCCGCAAGGTTGAAACTCAAAGGAATTGACGG

>OTU38

TAAAACGTAGGTGGCAAGCGTTGTCCGGAATTACTGGGTGTAAAGGGAGCGCAGGCGGATTGGTAAGTTGGAGGTGAAATTCATGGGCTCAACCCATGACCTGCCTTCAAAACTACCAGTCTTGAGTGGTGCAGAGGTAGGCGGAATTCCCGGTGTAGCGGTGGAATGCGTAGATATCGGGAGGAACACCAGTGGCGAAGGCGGCCTACTGGGCACTAACTGACGCTGAGGCTCGAAAGCATGGGTAGCAAACAGGATTAGATACCCTGGTAGTCCATGCCGTAAACGATGATTACTAGGTGTGGGGAGATTGACCCTCTCCGTGCCGCAGTTAACACAATAAGTAATCCACCTGGGGAGTACGACCGCAAGGTTGAAACTCAAAGGAATTGACGG

>OTU39

TAATACGTAGGgggCAAGCGTTATCCGGATTTACTGGGTGTAAAGGGAGCGTAGACGGCGGAGCAAGTCTGAAGTGAAAGCCCGGGGCTCAACCCCGGGACTGCTTTGGAAACTGTTCTGCTAGAGTGCTGGAGAGGTAAGTGGAATTCCTAGTGTAGCGGTGAAATGCGTAGATATTAGGAGGAACACCAGTGGCGAAGGCGGCTTACTGGACAGTAACTGACGTTGAGGCTCGAAAGCGTGGGGAGCAAACAGGATTAGATACCCTGGTAGTCCACGCCGTAAACGATGAATGCTAGGTGTTGGAGGGCAAAGCCCTTCGGTGCCGCCGCAAACGCAATAAGCATTCCACCTGGGGAGTACGTTCGCAAGAATGAAACTTAAATGAATTGACGG

>OTU40

TAATACAGAGGGTGCAAGCGTTAATCGGATTTACTGGGCGTAAAGCgcgcgTAGGTGGCCAATTAAGTCAAATGTGAAATCCCCGAGCTTAACTTGGGAATTGCATTCGATACTGGTTGGCTAGAGTATGGGAGAGGATGGTAGAATTCCAGGTGTAGCGGTGAAATGCGTAGAGATCTGGAGGAATACCGATGGCGAAGGCAGCCATCTGGCCTAATACTGACACTGAGGTGCGAAAGCATGGGGAGCAAACAGGATTAGATACCCTGGTAGTCCATGCCGTAAACGATGTCTACTAGCCGTTGGGGCCTTTGAGGCTTTAGTGGCGCAGCTAACGCGATAAGTAGACCGCCTGGGGAGTACGGTCGCAAGACTAAAACTTAAAGGAATTGACGG

>OTU41

TAATACAGAGGGTGCAAGCGTTAATCGGAATTACTGGGCGTAAAGCGAGCGTAGGTGGTCATTTAAGTCAGATGTGAAATCCCCGGGCTTAACCTGGGAACTGCATCTGATACTGGGTGACTAGAGTAGGTGAGAGGGAAGTAGAATTCCAGGTGTAGCGGTGAAATGCGTAGAGATCTGGAGGAATACCGATGGCGAAGGCAGCTTCCTGGCATCATACTGACACTGAGGTTCGAAAGCGTGGGTAGCAAACAGGATTAGATACCCTGGTAGTCCACGCCGTAAACGATGTCTACCAGTCGTTGGGTCTCTTGAAGACTTAGTGACGCAGTTAACGCGATAAGTAGACCGCCTGGGGAGTACGGCCGCAAGGTTAAAACTTAAATGAATTGACGG

>OTU42

TAATACGTAGGTGGCAAGCGTTGTCCGGATTTATTGGGCGTAAAGCGAGCGCAGGCGGTTTCTTAAGTCTGATGTGAAAGCCcccGGCTCAACCGGGGAGGGTCATTGGAAACTGGGAGACTTGAGTGCAGAAGAGGAGAGTGGAATTCCATGTGTAGCGGTGAAATGCGTAGATatatGGAGGAACACCAGTGGCGAAGGCGGCTctctGGTCTGTAACTGACGCTGAGGCTCGAAAGCGTGGGGAGCAAACAGGATTAGATACCCTGGTAGTCCACGCCGTAAACGATGAGTGCTAAGTGTTGGAGGGTTTCCGCCCTTCAGTGCTGCAGCTAACGCATTAAGCACTCCGCCTGGGGAGTACGACCGCAAGGTTGAAACTTAAATGAATTGACGGG

>OTU43

TAATACGTATGGAGCGAGCGTTGTCCGGAATTATTGGGCGTAAAGGGTACGCAGGCGGTTTAATAAGTCGAATGTTAAAGATCGGGGCTCAACCCCGTAAAGCATTGGAAACTGATAAACTTGAGTAGTGGAGAGGAAAGTGGAATTCCTAGTGTAGTGGTGAAATACGTAGATATTAGGAGGAATACCAGTAGCGAAGGCGACTTTCTGGACACAAACTGACGCTGAGGTACGAAAGCGTGGGGAGCAAACAGGATTAGATACCCTGGTAGTCCACGCCGTAAACGATGAATGCTAGGTGTTGGgggTCAAACCTCGGTGCCGAAGTTAACACATTAAGCATTCCGCCTGGGGAGTACGCACGCAAGTGTGAAACTTAAAGGAATTGACGG

>OTU44

TAATACGTATGGTGCAAGCGTTATCCGGATTTACTGGGTGTAAAGGGTGAGTAGGCGGTTATGCAAGTCATATGTGAAATTCTGGGGCTCAACCTCAGAGCTGCATAAGAAACTGTGTAACTAGAGTACAGGAGAGGTAAGCGGAATTCCTAGTGTAGCGGTGAAATGCGTAGATATTAGGAAGAACACCGGTGGCGAAGGCGGCTTACTGGACTGAAACTGACGCTGAGTCACGAAAGCGTGGGGAGCGAACAGGATTAGATACCCTGGTAGTCCACGCCGTAAACGATGAGTGCTAGGTGTTGGGAAGAGATTCTCGGTGCCGCAGCAAACGCAATAAGCACTCCACCTGGGGAGTACGACCGCAAGGTTGAAACTTAAATGAATTGACGGG

>OTU45

TAATACGTAGGTGGCGAGCGTTATCCGGAATGATTGGGCGTAAAGGGTGCGTAGGTGGCAGAACAAGTCTGGAGTAAAAGGTATGGGCTCAACCCGTACTGGCTCTGGAAACTGTTCAGCTAGAGAACAGAAGAGGACGGCGGAACTCCATGTGTAGCGGTAAAATGCGTAGATatatGGAAGAACACCGGTGGCGAAGGCGGCCGTCTGGTCTGTTGCTGACACTGAAGCACGAAAGCGTGGGGAGCAAATAGGATTAGATACCCTAGTAGTCCACGCCGTAAACGATGAGAACTAGGTGTTGGgggAATAACTCAGTGCCGCAGTTAACGCAATAAGTTCTCCGCCTGGGGAGTATGCACGCAAGTGTGAAACTTAAAGGAATTGACGG

>OTU46

TAATACGTAGGGTGCGAGCGTTGTCCGGATTTACTGGGCGTAAAGAGCTCGTAGGCGGTTTGTCACGTCGTCTGTGAAAACCCGAGGCTTAACCTCGGGCCTGCAGGCGATACGGGCAGACTTGAGTACTGTAGGGGAGACTGGAATTCCTGGTGTAGCGGTGGAATGCGCAGATATCAGGAGGAACACCGGTGGCGAAGGCGGGTCTCTGGGCAGTAACTGACGCTGAGGAGCGAAAGCGTGGGTAGCGAACAGGATTAGATACCCTGGTAGTCCACGCCGTAAACGGTGGGTACTAGGTGTGGGTTTCCTTCCACGGGATCCGTGCCGTAGCTAACGCATTAAGTACCCCGCCTGGGGAGTACGGCCGCAAGGCTAAAACTCAAATGAATTGACGG

>OTU47

TAATACAGAGGGTGCAAGCGTTGTTCGGAATTATTGGGCGTAAAGGGCGCGTAGGCGGTGCGGTAAGTCTGCGGTGAAATCTCCCGGCTCAACTGGGAGGCTGCCGTGGAAACTGCCGTGCTTGAGTGTGGGAGAGGTGAGTGGAATTCCCGGTGTAGCGGTGAAATGCGTAGATATCGGGAGGAACACCTGTGGCGAAAGCGGCTCACTGGACCACAACTGACGCTGAGGCGCGAAAGCTAGGGGAGCAAACAGGATTAGATACCCTGGTAGTCCTAGCCCTAAACGATGATTGCTTGGTGTAGCAGGTACCCAATCCTGCTGTGCCGCAGCTAACGCGTTAAGCAATCCGCCTGGGGAGTACGGTCGCAAGGCTGAAACTTAAAGGAATTGACGG

>OTU48

TAATACGGAGGGTGCAAGCGTTACTCGGAATCACTGGGCGTAAAGGACGCGTAGGCGGATTATCAAGTCTCTTGTGAAATCCTATGGCTTAACCATAGAACTGCTTGGGAAACTGATAATCTAGAGTGAGGGAGAGGCAGATGGAATTGGTGGTGTAGGGGTAAAATCCGTAGAGATCACCAGGAATACCCATTGCGAAGGCGATCTGCTGGAACTCAACTGACGCTAATGCGTGAAAGCGTGGGGAGCAAACAGGATTAGATACCCTGGTAGTCCACGCCCTAAACGATGTATACTAGTTGTTGCTAAGCTAGTCTTGGCAGTAATGCACCTAACGGATTAAGTATACCGCCTGGGGAGTACGGTCGCAAGATTAAAACTTAAAGGAATTGACGG

>OTU49

TAATACGGAGGGCGCGAGCGTTACCCGGATTCACTGGGCGTAAAGGGCGTGTAGGCGGCCTGGGGCGTCCCATGTGAAAGACCACGGCTCAACCGTGGgggAGCGTGGGATACGCTCAGGCTAGACGGTGGGAGAGGGTGGTGGAATTCCCGGAGTAGCGGTGAAATGCGCAGATACCGGGAGGAACGCCGATGGCGAAGGCAGCCACCTGGTCCACCCGTGACGCTGAGGCGCGAAAGCGTGGGGAGCAAACCGGATTAGATACCCGGGTAGTCCACGCCCTAAACGATGCgcgcTAGGTCTCTGGGTTTTCTGGgggCCGAAGCTAACGCGTTAAGCgcgcCGCCTGGGGAGTACGGCCGCAAGGCTGAAACTTAAAGGAATTGACGG

>OTU50

TAATACGTAGGTGGCAAGCGTTGTCCGGAATTATTGGGCGTAAAGGGCTCGCAGGCGGTTTCTTAAGTCTGATGTGAAAGCCcccGGCTCAACCGGGGAGGGTCATTGGAAACTGGGAAACTTGAGTGCAGAAGAGGAGAGTGGAATTCCACGTGTAGCGGTGAAATGCGTAGAGATGTGGAGGAACACCAGTGGCGAAGGCGACTctctGGTCTGTAACTGACGCTGAGGAGCGAAAGCGTGGGGAGCGAACAGGATTAGATACCCTGGTAGTCCACGCCGTAAACGATGAGTGCTAAGTGTTAGGgggTTTCCGCCCCTTAGTGCTGCAGCTAACGCATTAAGCACTCCGCCTGGGGAGTACGGTCGCAAGACTGAAACTTAAAGGAATTGACGG

>OTU51

TAATACGTAGGTGGCAAGCGTTATCCGGAATCATTGGGCGTAAAGGGTGCGTAGGTGGCGTACTAAGTCTGTAGTAAAAGGCAATGGCTCAACCATTGTAAGCTATGGAAACTGGTATGCTGGAGTGCAGAAGAGGGCGATGGAATTCCATGTGTAGCGGTAAAATGCGTAGATatatGGAGGAACACCAGTGGCGAAGGCGGTCGCCTGGTCTGTAACTGACACTGAGGCACGAAAGCGTGGGGAGCAAATAGGATTAGATACCCTAGTAGTCCACGCCGTAAACGATGAGAACTAAGTGTTGGAGGAATTCAGTGCTGCAGTTAACGCAATAAGTTCTCCGCCTGGGGAGTATGCACGCAAGTGTGAAACTCAAATGAATTGACGG

>OTU52

TAATACGGAGGATGCGAGCGTTATCCGGATTTATTGGGTTTAAAGGGTGCGTAGGTTGTTGTTTAAGTCAGCGGTGAAAGTTTGTCGCTTAACGATAAAATTGCCGTTGAAACTGGACGACTTGAGTgtgtTTGAGGTAGGCGGAATGCGTGGTGTAGCGGTGAAATGCATAGATATCACGCAGAACTCCGATTGCGAAGGCAGCTTACTAAACCATAACTGACACTGAAGCACGAAAGCGTGGGTATCAAACAGGATTAGATACCCTGGTAGTCCACGCAGTAAACGATGATTACTAGGAGTTTGCGATATAGTGTAAGCTCTACAGCGAAAGCGTTAAGTAATCCACCTGGGGAGTACGCCGGCAACGGTGAAACTTAAATGAATTGACGGG

>OTU53

TAATACGAAGGGTGCAAGCGTTACTCGGAATTACTGGGCGTAAAGCGTGCGTAGGTGGTCGTTTAAGTCCGTTGTGAAAGCCCTGGGCTCAACCTGGGAACTGCAGTGGATACTGGGCGACTAGAGTGTGGTAGAGGGTAGCGGAATTCCTGGTGTAGCAGTGAAATGCGTAGAGATCAGGAGGAACATCCATGGCGAAGGCAGCTACCTGGACCAACACTGACACTGAGGCACGAAAGCGTGGGGAGCAAACAGGATTAGATACCCTGGTAGTCCACGCCCTAAACGATGCGAACTGGATGTTGGGTGCAATTTGGCACGCAGTATCGAAGCTAACGCGTTAAGTTCGCCGCCTGGGGAGTACGGTCGCAAGACTGAAACTTAAATGAATTGACGG

>OTU54

TAATACGGggggTGCAAGCGTTGTTCGGATTTATTGGGCGTAAAGCGCGTGTAGGCGGTTttttAAGTCTGATGTGAAAGCCCCGGGCTCAGCCTGGAAGTGCATTGGATACTGGAAGACTTGAATACGGGAGAGGGCAGTGGAATTCCTGGTGTAGGAGTGAAATCCGTAGATATCAGGAGGAACACCGGTGGCGAAGGCGGCTGCCTGGACCGATATTGACGCTGAGACGCGAAAGCGTGGGTAGCAAACAGGATTAGATACCCTGGTAGTCCACGCCGTAAACGATGAGTACTAGGTGTTGCGGGTATTGACCCCTGCAGTGCCGCAGCTAACGCATTAAGTACTCCGCCTGGGAAGTACGGTCGCAAGACTAAAACTTAAATGAATTGACGG

>OTU55

TAATACGGAGGGTGCAAGCGTTATCCGGATTTATTGGGTTTAAAGGGTCCGTAGGCGGGCCCGTAAGTCAGTGGTGAAATCTCGCAGCTTAACTGCGAAACTGCCATTGATACTGCGGGTCTTGAGTGAATTTGAAGTGGCTGGAATAAGTAGTGTAGCGGTGAAATGCATAGATATTACTTAGAACACCGATTGCGAAGGCAGGTCACTAAGATTCAACTGACGCTGAGGGACGAAAGCGTGGGGAGCGAACAGGATTAGATACCCTGGTAGTCCACGCCGTAAACGATGCTAACTCGTTTTCGGGCCGCAAGGCTCGGAGACCAAGCGAAAGTGATAAGTTAGCCACCTGGGGAGTACGTCCGCAAGGATGAAACTCAAATGAATTGACGG

>OTU56

TAATACGGAGGATACGAGCGTTATCCGGATTTATTGGGTTTAAAGGGTGCGCAGGTGGTCCTGCAAGTCAGTGGTGAAAAGCTGAGGCTCAACCTCAGCCTTGCCGTTGAAACTGCAAGACTTGAGAGTACATGATGTGGGCGGAATGCGTAGTGTAGCGGTGAAATGCATAGATATTACGCAGAACTCCGATTGCGAAGGCAGCTCACAAAGGTATATCTGACACTGAGGCACGAAAGCGTGGGGAGCAAACAGGATTAGATACCCTGGTAGTCCACGCCGTAAACGATGATTACTCGAAGTATGCGATATGACAGTATGCTTCCAAGCGAAAGTGATAAGTAATCCACCTGGGGAGTACGCCGGCAACGGTGAAACTCAAATGAATTGACGG

>OTU57

TAATACGTAGGgggCAAGCGTTATCCGGAATTACTGGGTGTAAAGGGTGCGTAGGTGGCATGATAAGTCAGAAGTGAAAGCCCGGGGCTTAACCCCGGGACTGCTTTTGAAACTGTAATGCTAGAGTGCAGGAGAGGTAAGCGGAATTCCTAGTGTAGCGGTGAAATGCGTAGATATTAGGAGGAACACCAGTGGCGAAGGCGGCTTACTGGACTGTCACTGACACTGAGGCACGAAAGCGTGGGGAGCAAACAGGATTAGATACCCTGGTAGTCCACGCCGTAAACGATGAATACTAGGTGTCGGGGCCGTAGAGGCTTCGGTGCCGCAGCAAACGCAGTAAGTATTCCACCTGGGGAGTACGTTCGCAAGAATGAAACTTAAATGAATTGACGG

>OTU58

TAATACGTAGGTGGCAAGCGTTGTCCGGAATTATTGGGCGTAAAGCgcgcgcAGGCGGATCTGCCAGTCTGTCTTAAAAGTTCGGGGCTCAACCCCGTGATGGGATGGAAACTACAGATCTAGAGTATCGGAGGGGAAAGTGGAATTCCTAGTGTAGCGGTGAAATGCGTAGATATTAGGAGGAACACCAGTGGCGAAGGCGACTTTCTGGACGATCACTGACGCTGAGGCGCGAAAGCCAGGGGAGCGAACGGGATTAGATACCCCGGTAGTCCTGGCCGTAAACGATGGGTACTAGGTGTAGGAGGTATCGACCCCTTCTGTGCCGTAGTTAACGCAGTAAGTACCCCGCCTGGGGAGTACGGCCGCAAGGTTGAAACTTAAAGGAATTGACGGG

>OTU59

TAATACGGAGGgggCGAGCGTTGTTCGGAATCACTGGGCGTAAAGAGTACGTAGGCGGTTTTGCAAGTCAGAAGTGAAAGCCCAGGGCTTAACCTTGGAACTGCTTTTGAAACTGCAAAACTAGAGTCCCGGAGAGGTTGGCGGAATTCCGAGTGTAGAGGTGAAATTCGCAGATATTCGGAGGAACACCAGTGGCGTAAGCGGCCAACTGGACGGGAACTGACGCTGAGGTACGAAAGCGTGGGGAGCAAACAGGATTAGATACCCTGGTAGTCCACGCCATAAACGATGtgtgCTAGCTGTCGGGAGTTATGCTCTCGGTGGCGGACCTAACGGATTAAGCACACCGCCTGGGGAGTACGGTCGCAAGATTAAAACTTAAATGAATTGACGG

>OTU60

TAATACGTAGGGTGCGAGCGTTGTCCGGAATTACTGGGCGTAAAGAGCTCGTAGGTGGTTTGTCGCGTCGTCTGTGAAATTCCGGGGCTTAACTCCGGGCGTGCAGGCGATACGGGCATAACTTGAGTGCTGTAGGGGAGACTGGAATTCCTGGTGTAGCGGTGAAATGCGCAGATATCAGGAGGAACACCGATGGCGAAGGCAGGTCTCTGGGCAGTTACTGACGCTGAGGAGCGAAAGCATGGGTAGCGAACAGGATTAGATACCCTGGTAGTCCATGCCGTAAACGGTGGGCGCTAGGTGTAGGgggCTTCCACGTCTTCTGTGCCGTAGCTAACGCATTAAGCGCCCCGCCTGGGGAGTACGGCCGCAAGGCTAAAACTCAAAGGAATTGACGG

>OTU61

TAATACGTAGGGTGCGAGCGTTGTCCGGAATTATTGGGCGTAAAGGGCTCGTAGGCGGTTTGTCGCGTCGGGAGTGAAAACTCAGGGCTTAACTCTGAGCTTGCTTCCGATACGGGCAGACTAGAGGTATGCAGGGGAGAACGGAATTCCTGGTGTAGCGGTGAAATGCGCAGATATCAGGAGGAACACCGGTGGCGAAGGCGGTTCTCTGGGCATCACCTGACGCTGAGGAGCGAAAGTGTGGGGAGCGAACAGGATTAGATACCCTGGTAGTCCACACCGTAAACGTTGGGCGCTAGGTGTGGGGTCCATTCCACGGATTCCGTGCCGCAGCTAACGCATTAAGCGCCCCGCCTGGGGAGTACGGCCGCAAGGCTAAAACTCAAATGAATTGACGG

>OTU62

TAATACGAAGGGTGCAAGCGTTAATCGGAATTACTGGGCGTAAAGCgcgcgTAGGTGGTTCAGCAAGTTGGAGGTGAAATCCCCGGGCTCAACCTGGGAACTGCCTCCAAAACTACTGAGCTAGAGTACGGTAGAGGGTAGTGGAATTTCCTGTGTAGCGGTGAAATGCGTAGATATAGGAAGGAACACCAGTGGCGAAGGCGACTACCTGGACTGATACTGACACTGAGGTGCGAAAGCGTGGGGAGCAAACAGGATTAGATACCCTGGTAGTCCACGCCGTAAACGATGTCGACTAGCCGTTGGGATCCTTGAGATCTTAGTGGCGCAGCTAACGCATTAAGTCGACCGCCTGGGGAGTACGGCCGCAAGGTTAAAACTCAAAGGAATTGACGG

>OTU63

TAATACGTAGGgggCAAGCGTTATCCGGATTTACTGGGTGTAAAGGGAGCGTAGACGGCTTAGCAAGTCTGAAGTGAAAGCCCGGGGCTCAACCCCGGGACTGCTTTGGAAACTGTTAAGCTGGAGTGCTGGAGAGGTAAGCGGAATTCCTAGTGTAGCGGTGAAATGCGTAGATATTAGGAGGAACACCAGTGGCGAAGGCGGCTTACTGGACAGTAACTGACGTTGAGGCTCGAAAGCGTGGGGAGCAAACAGGATTAGATACCCTGGTAGTCCACGCCGTAAACGATGAATACTAGGTGTTGGTGGGCAAAGCCCATCGGTGCCGTCGCAAACGCAATAAGTATTCCACCTGGGGAGTACGTTCGCAAGAATGAAACTTAAAGGAATTGACGG

>OTU64

TAATACGTAGGTGGCAAGCGTTATCCGGATTTATTGGGCGTAAAGCGAGCGCAGGCGGTTTGATAAGTCTGATGTGAAAGCCTTTGGCTTAACCAAAGAAGTGCATCGGAAACTGTCAGACTTGAGTGCAGAAGAGGACAGTGGAACTCCATGTGTAGCGGTGGAATGCGTAGATatatGGAAGAACACCAGTGGCGAAGGCGGCTGTCTGGTCTGCAACTGACGCTGAGGCTCGAAAGCATGGGTAGCGAACAGGATTAGATACCCTGGTAGTCCATGCCGTAAACGATGAGTGCTAGGTGTTGGAGGGTTTCCGCCCTTCAGTGCCGCAGCTAACGCATTAAGCACTCCGCCTGGGGAGTACGACCGCAAGGTTGAAACTTAAATGAATTGACGG

>OTU65

TAATACGTAGGgggCTAGCGTTATCCGGAATTACTGGGCGTAAAGGGTGCGTAGGTGGTTTCTTAAGTCAGAGGTGAAAGGCTACGGCTCAACCGTAGTAAGCCTTTGAAACTGAGAAACTTGAGTGCAGGAGAGGAGAGTAGAATTCCTAGTGTAGCGGTGAAATGCGTAGATATTAGGAGGAATACCAGTTGCGAAGGCGGCTctctGGACTGTAACTGACACTGAGGCACGAAAGCGTGGGGAGCAAACAGGATTAGATACCCTGGTAGTCCACGCCGTAAACGATGAGTACTAGCTGTCGGAGGTTACCcccTTCGGTGGCGCAGCTAACGCATTAAGTACTCCGCCTGGGAAGTACGCTCGCAAGAGTGAAACTTAAATGAATTGACGG

>OTU66

TAATACGTAGGTCCCGAGCGTTGTCCGGATTTATTGGGCGTAAAGCGAGCGCAGGCGGTTTGATAAGTCTGAAGTTAAAGGCTGTGGCTCAACCATAGTTCGCTTTGGAAACTGTCAAACTTGAGTGCAGAAGGGGAGAGTGGAATTCCATGTGTAGCGGTGAAATGCGTAGATatatGGAGGAACACCGGTGGCGAAAGCGGCTctctGGTCTGTAACTGACGCTGAGGCTCGAAAGCGTGGGGAGCGAACAGGATTAGATACCCTGGTAGTCCACGCCGTAAACGATGAGTGCTAGGTGTTGGATCCTTTCCGGGATTCAGTGCCGCAGCTAACGCATTAAGCACTCCGCCTGGGGAGTACGACCGCAAGGTTGAAACTTAAATGAATTGACGGG

>OTU67

TAATACGGAGGATGCGAGCGTTATCCGGATTTATTGGGTTTAAAGGGAGCGCAGACGGGGAGTTAAGTCAGCTGTGAAAGTTTGCGGCTCAACCGTAAAATTGCAGTTGATACTGGCCCTCTTGAATGCAGTTGAGGTAGGCGGAATTCGTGGTGTAGCGGTGAAATGCTTAGATATCACGAAGAACTCCGATTGCGAAGGCAGCTTACTAAACTGTAACTGACGTTCATGCTCGAAAGTGTGGGTATCAAACAGGATTAGATACCCTGGTAGTCCACACGGTAAACGATGAATACTCGCTGTTGGCGATATACGGTCAGCGGCCAAGCGAAAGCATTAAGTATTCCACCTGGGGAGTACGCCGGCAACGGTGAAACTTAAAGGAATTGACGG

>OTU68

TAAGACGAACCGTGCGAACGTTGTTCGGAATCACTGGGCTTAAAGGGCGCGTAGGCGGGCTGTCCAGTCTGGGGTGAAATCCCGCGGCTCAACCGTGGAACTGCCTCAGATACTGACGGCCTCGAGGGAGATAGGGGCATGCGGAACTGTAGGTGGAGCGGTGAAATGCGTTGATATCTACAGGAACTCCGGTGGCGAAGGCGGCGTGCTGGATCTCTTCTGACGCTGAGGCGCGAAAGCTAGGGGAGCAAACGGGATTAGATACCCCGGTAGTCCTAGCCCTAAACGATGGGTACTAGATAGTAGGCTAGATATGGGCTTACTGTCGAAGCTAAAGTGCTAAGTACCCCGCCTGGGGAGTATGGTCGCAAGGCTGAAACTTAAATGAATTGACGG

>OTU69

TAACACCGGCAGCCCAAGTGGTGGCCATTtttATTGGGCCTAAAGCGTTCGTAGCCGGCCTGATAAGTCTCTGGTGAAATCCCGCAGCTTAACTGTGGGAATTGCTGGAGATACTATCAGGCTTGAGGTCGGGAGAGGTTAGAGGTACTCCCAGGGTAGGGGTGAAATCCTATAATCCTGGGAGGACCACCTGTGGCGAAGGCGTCTAACTGGAACGAACCTGACGGTGAGTAACGAAAGCCAGGGGCGCGAACCGGATTAGATACCCGGGTAGTCCTGGCCGTAAACGATGTGGACTTGGTGTTGGGATGGCCTCGAGCTGCCCCAGTGCCGAAGGGAAGCTGTTAAGTCCACCGCCTGGGAAGTACGGTCGCAAGACTGAAACTCAAATGAATTGACGG

>OTU70

TAATACGTAGGgggCAAGCGTTATCCGGATTTACTGGGTGTAAAGGGAGCGTAGACGGCATGGCAAGTCTGGTGTGAAAGGCAGGGGCTCAACCCCTGGACTGCATTGGAAACTGTCAGGCTGGAGTGCAGGAGAGGTAAGTGGAATTCCTAGTGTAGCGGTGAAATGCGTAGATATTAGGAGGAACACCAGTGGCGAAGGCGACTTACTGGACTGTAACTGACGTTGAGGCTCGAAAGCGTGGGGAGCAAACAGGATTAGATACCCTGGTAGTCCACGCCGTAAACGATGAATACTAGGTGTCAGGGAGCAAAGCTCTTTGGTGCCGCAGCTAACGCAATAAGTATTCCACCTGGGGAGTACGTTCGCAAGAATGAAACTCAAATGAATTGACGGG

>OTU71

TAATACGGAGGGAGCTAGCGTTGTTCGGAATTACTGGGCGTAAAGCGCACGTAGGCGGCTTTGTAAGTTAGAGGTGAAAGCCTGGAGCTCAACTCCAGAACTGCCTTTAAGACTGCATCGCTTGAATCCAGGAGAGGTGAGTGGAATTCCGAGTGTAGAGGTGAAATTCGTAGATATTCGGAAGAACACCAGTGGCGAAGGCGGCTCACTGGACTGGTATTGACGCTGAGGTGCGAAAGCGTGGGGAGCAAACAGGATTAGATACCCTGGTAGTCCACGCCGTAAACGATGATAACTAGCTGTCCGGGGACTTGGTCTTTGGGTGGCGCAGCTAACGCATTAAGTTATCCGCCTGGGGAGTACGGCCGCAAGGTTAAAACTTAAATGAATTGACGG

>OTU72

TAATACGTAGGTGGCGAGCGTTGTCCGGAATCATTGGGCGTAAAGGGAGCGCAGGCGGGCATGTAAGTCTTTCTTAAAAGTGCGGGGCTCAACCCCGTGATGGGAAAGAAACTATGTGTCTTGAGTACAGGAGAGGAAAGCGGAATTCCCAGTGTAGCGGTGAAATGCGTAGATATTGGGAGGAACACCAGTGGCGAAGGCGGCTTTCTGGACTGCAACTGACGCTGAGGCTCGAAAGCCAGGGGAGCGAACGGGATTAGATACCCCGGTAGTCCTGGCCGTAAACGATGGATACTAGGTGTGGGAGGTATCGACCCCTACCGTGCCGGAGTTAACGCAATAAGTATCCCGCCTGGGGAGTACGGCCGCAAGGCTGAAACTTAAATGAATTGACGG

>OTU73

TAATACGTAGGgggCAAGCGTTATCCGGATTTACTGGGTGTAAAGGGAGCGTAGACGGCATATAAAGTCTGAAGTGAAATCCCGCAGCTCAACTGCGGAGTTGCTTTGGAAACTTATAAGCTGGAGTGTCGGAGGGGTAAGCGGAATTCCCAGTGTAGCGGTGAAATGCGTAGATATTGGGAGGAACACCGGAGGCGAAGGCGGCTTACTGGAAGATAACTGACGTTGAGGCTCGAAGGCGTGGGTAGCAAACAGGATTAGATACCCTGGTAGTCCACGCAGTAAACGATGAATACTAGGTGTCGGGAGGGATGACCCTTTCGGTGCCGTCGCAAACGCAATAAGTATTCCACCTGGGGAGTACGTTCGCAAGAATGAAACTCAAATGAATTGACGG

>OTU74

TAATACGTAGGTCCCGAGCGTTGTCCGGATTTATTGGGCGTAAAGCGAGCGCAGGTGGTTTATTAAGTCTGGTGTAAAAGGCAGTGGCTCAACCATTGTATGCATTGGAAACTGGTAGACTTGAGTGCAGGAGAGGAGAGTGGAATTCCATGTGTAGCGGTGAAATGCGTAGATatatGGAGGAACACCGGTGGCGAAAGCGGCTctctGGCCTGTAACTGACACTGAGGCTCGAAAGCGTGGGGAGCAAACAGGATTAGATACCCTGGTAGTCCACGCCGTAAACGATGAGTGCTAGATGTAGGGAGCTATAAGTTCTCTGTATCGCAGCTAACGCAATAAGCACTCCGCCTGGGGAGTACGACCGCAAGGTTGAAACTCAAAGGAATTGACGG

>OTU75

TAATACGTAGGTGGCGAGCGTTATCCGGAATTATTGGGCGTAAAGAGGGAGCAGGCGGCAGCAGAGGTCTGTGGTGAAAGACTGAAGCTTAACTTCAGTAAGCCATAGAAACCGGGCAGCTAGAGTGCAGGAGAGGATCGTGGAATTCCATGTGTAGCGGTGAAATGCGTAGATatatGGAGGAACACCAGTGGCGAAGGCGACGATCTGGCCTGTAACTGACGCTCATTCCCGAAAGCGTGGGGAGCAAATAGGATTAGATACCCTAGTAGTCCACGCCGTAAACGATGAGTACTAAGTGTTGGGAGTCAAATCTCAGTGCTGCAGTTAACGCAATAAGTACTCCGCCTGAGTAGTACGTTCGCAAGAATGAAACTTAAAGGAATTGACGG

>OTU76

TAATACGGAGGGTGCGAGCGTTAATCGGAATAACTGGGCGTAAAGGGCACGCAGGCGGTGACTTAAGTGAGGTGTGAAAGCCCCGGGCTTAACCTGGGAATTGCATTTCATACTGGGTCGCTAGAGTACTTTAGGGAGGGGTAGAATTCCACGTGTAGCGGTGAAATGCGTAGAGATGTGGAGGAATACCGAAGGCGAAGGCAGCCCCTTGGGAATGTACTGACGCTCATGTGCGAAAGCGTGGGGAGCAAACAGGATTAGATACCCTGGTAGTCCACGCTGTAAACGATGTCGATTTGGgggTTGAACTTTAAGTTTGGCGCCCGTAGCTAACGTGATAAATCGACCGCCTGGGGAGTACGGCCGCAAGGTTAAAACTTAAATGAATTGACGGG

>OTU77

TAATACGGAGGGAGCTAGCGTTGTTCGGAATTACTGGGCGTAAAGCGCACGTAGGCGGCGATTCAAGTCAGAGGTGAAAGCCCGGGGCTCAACCCCGGAACTGCCTTTGAAACTAGATTGCTAGAATCCTGGAGAGGCGAGTGGAATTCCGAGTGTAGAGGTGAAATTCGTAGATATTCGGAAGAACACCAGTGGCGAAGGCGACTCGCTGGACAGGTATTGACGCTGAGGTGCGAAAGCGTGGGGAGCAAACAGGATTAGATACCCTGGTAGTCCACGCCGTAAACGATGATAACTAGCTGTCCGGGCACATGGTGCTTGGGTGGCGCAGCTAACGCATTAAGTTATCCGCCTGGGGAGTACGGTCGCAAGATTAAAACTTAAATGAATTGACGG

>OTU78

TAAGACAGAGGGTGCAAGCGTTGTTCGGAATTATTGGGCGTAAAGGGCGCGTAGGCGGCCCATCAAGTCAGATGTGAAAGCCCAGGGCTCAACCTTGGAAGTGCATCTGAAACTGATGAGCTAGAGTTCACGGAGAGGGTGGTGGAATTCCCAGTGTAGAGGTGAAATTCGTAGATATTGGGAGGAACACCGGTGGCGAAGGCGACCACCTGGACGGAAACTGACGCTGAGGCGCGAAAGCGTGGGTAGCAAACAGGATTAGATACCCTGGTAGTCCACGCCGTAAACGATGAGTATTAGGTATTGCGGGTATTGACCCCTGCAGTGCCGTCGCTAACGCATTAAATACTCCGCCTGGGAAGTACGGCCGCAAGGCTAAAACTCAAAGGAATTGACGG

>OTU79

CCATCATGTGAACGATTGGAACCGCAACTGTCCGAACGATCATTGTTAAGAACAAACCTGCAACCGCTAAAGGAACTGCGATGGCAATCGCTGAAGGAACACCTTTAACACCTTGGCCACCTAAAACTAAAATAATTGCTGAGGCAACAGATGCTAAAGCGGCATCTGGTGCAACGGCAGCCCCGATGTTCGCCCAACCTAACGCGATCATTTGTAAAGTACCACCTAAAACAATTCCGGCTTCTAAATTACCAGTAACTAATCCGATTAATGTACATGCTACTAACGGTTGGTGGAATTGGAACTCATCCAAAATCCCTTCCATACCAGCTAGAAAGGCAACGAGTAAAACTTAAATGAATTGACGG

>OTU80

TAATACGTAGGGAGCAAGCGTTGTCCGGATTTACTGGGTGTAAAGGGTGCGTAGGCGGCTAGGCAAGTCAGACGTGAAAACCATGGGCTCAACCTGTGGACTGCGTTTGAAACTGTTTAGCTTGAGTGAAGTAGAGGTAGGCGGAATTCCCGGTGTAGCGGTGAAATGCGTAGAGATCGGGAGGAACACCAGTGGCGAAGGCGGCCTACTGGGCTTTAACTGACGCTGAGGCACGAAAGCATGGGTAGCAAACAGGATTAGATACCCTGGTAGTCCATGCCGTAAACGATGATTACTAGGTGTGGggggTCTGACCcccTCCGTGCCGGAGTTAACACAATAAGTAATCCACCTGGGGAGTACGGCCGCAAGGTTGAAACTCAAATGAATTGACGG

>OTU81

TAATACGTAGGgggCAAGCGTTATCCGGATTTACTGGGTGTAAAGGGAGCGTAGACGGCGAAGCAAGTCTGATGTGAAATACCGGGGCTCAACCCCGGGACTGCATTGGAAACTGTTTTGCTTGAGTGCCGGAGAGGTAAGCGGAATTCCTAGTGTAGCGGTGAAATGCGTAGATATTAGGAGGAACACCAGTGGCGAAGGCGGCTTACTGGACGGTAACTGACGTTGAGGCTCGAAAGCGTGGGGAGCAAACAGGATTAGATACCCTGGTAGTCCACGCCGTAAACGATGAATGCTAGGTGTTGGTGAGCAAAGCTCATCGGTGCCGCCGCAAACGCATTAAGCATTCCACCTGGGGAGTACGTTCGCAAGAATGAAACTTAAATGAATTGACGG

>OTU82

TAATACGTAGGGCGCAAGCGTTGTCCGGAATTATTGGGCGTAAAGGGCTTGTAGGTGGCTTGTCGCGTCTGCCGTGAAAACTCGAGGCTTAACCTTGGGCGTGCGGTGGGTACGGGCAGGCTAGAGTGCGGTAGGGGAGACTGGAATTCCTGGTGTAGCGGTGAAATGCGCAGATATCAGGAGGAACACCGATGGCGAAGGCAGGTCTCTGGGCCGTTACTGACACTGAGAAGCGAAAGCATGGGGAGCGAACAGGATTAGATACCCTGGTAGTCCATGCCGTAAACGTTGGGCACTAGGTGTGGGGAGCATTCCACGTTTTCCGCGCCGTAGCTAACGCATTAAGTGCCCCGCCTGGGGAGTACGGCCGCAAGGCTAAAACTCAAATGAATTGACGG

>OTU83

TAATACGTAGGGTGCAAGCGTTGTCCGGAATTACTGGGCGTAAAGAGCTCGTAGGTGGTTTGTCACGTCGTCTGTGAAATTCCACAGCTTAACTGTGGGCGTGCAGGCGATACGGGCTGACTTGAGTACTGTAGGGGTAACTGGAATTCCTGGTGTAGCGGTGAAATGCGCAGATATCAGGAGGAACACCGATGGCGAAGGCAGGTTACTGGGCAGTTACTGACGCTGAGGAGCGAAAGCATGGGTAGCAAACAGGATTAGATACCCTGGTAGTCCATGCCGTAAACGGTGGGCGCTAGGTGTAGGGGCCTTTTGGGGTTTCTGTGCCGTAGCTAACGCATTAAGCGCCCCGCCTGGGGAGTACGGCCGCAAGGCTAAAACTTAAATGAATTGACGG

>OTU84

TAATACGGAGGGTGCAAGCGTTAATCGGAATCACTGGGCGTAAAGCGCACGTAGGCGGCTTGGTAAGTCAGGGGTGAAATCCCACAGCCCAACTGTGGAACTGCCTTTGATACTGCCAGGCTTGAGTACCGGAGAGGGTGGCGGAATTCCAGGTGTAGGAGTGAAATCCGTAGATATCTGGAGGAACACCGGTGGCGAAGGCGGCCACCTGGACGGTAACTGACGCTGAGGTGCGAAAGCGTGGGTAGCAAACAGGATTAGATACCCTGGTAGTCCACGCTGTAAACGATGGGTGCTGGGTGCTGGGATGTATGTCTCGGTGCCGTAGCTAACGCGATAAGCACCCCGCCTGGGGAGTACGGTCGCAAGGCTGAAACTTAAAGGAATTGACGG

>OTU85

TAATACGTAGGTGGCAAGCGTTATCCGGATTTATTGGGTGTAAAGGGCGTGTAGGCGGGACTGCAAGTCAGATGTGAAAACCACGGGCTCAACCTGTGGCCTGCATTTGAAACTGTAGTTCTTGAGTGTCGGAGAGGCAATCGGAATTCCGTgtgtAGCGGTGAAATGCGTAGATATACGGAGGAACACCAGTGGCGAAGGCGGATTGCTGGACGATAACTGACGCTGAGGCGCGAAAGCGTGGGGAGCAAACAGGATTAGATACCCTGGTAGTCCACGCCGTAAACGATGGATACTAGGTGTGGggggACTGACCcccTCCGTGCCGCAGTTAACACAGTAAGTATCCCACCTGGGGAGTACGATCGCAAGGTTGAAACTCAAATGAATTGACGG

>OTU86

TAATACAGAGGgggCAAGCGTTATTCGGAATTATTGGGCGTAAAGGGCGCGTAGGCGGCCTGTCAAGTCtctcGTGAAATCCCTCGGCTTAACTGAGGACGTGCGGGAGATACTGTCAGGCTGGAGTCCGGAAGAGGGTGGTGGAATTCCCAGTGTAGCGGTGAAATGCGTAGATATTGGGAGGAACACCGGTGGCGAAGGCGGCCACCTGGGCCGGAACTGACGCTGAGGCGCGAAAGCGTGGGGAGCAAACGGGATTAGATACCCCGGTAGTCCACGCCGTAAACGATGGGCACTTGGTGTCGCGGGTATCGACCCCTGCGGTGCCGAAGCTAACGCATTAAGTGCCCCGCCTGGGGAGTACGGTCGCAAGGCTGAAACTTAAATGAATTGACGG

>OTU87

TAATACGTAGGGTGCAAGCGTTGTCCGGAATTATTGGGCGTAAAGAGCTCGTAGGCGGCTTGTCGCGTCTGCTGTGAAATCCCGGGGCTCAACCCCGGGCCTGCAGTGGGTACGGGCAAGCTAGAGTGCGGTAGGGGAGATTGGAATTCCTGGTGTAGCGGTGGAATGCGCAGATATCAGGAGGAACACCGATGGCGAAGGCAGATCTCTGGGCCGCTACTGACGCTGAGGAGCGAAAGCATGGGGAGCGAACAGGATTAGATACCCTGGTAGTCCATGCCGTAAACGTTGGGAACTAGATGTAGGGCCTGTTCCACGGGTTCTGTGTCGTAGCTAACGCATTAAGTTCCCCGCCTGGGGAGTACGGCCGCAAGGCTAAAACTTAAATGAATTGACGG

>OTU88

TAATACGTAGGTGGCGAGCGTTATCCGGAATTATTGGGCGTAAAGAGCgcgcAGGTGGTTGATTAAGTCTGATGTGAAAGCCCACGGCTTAACCGTGGAGGGTCATTGGAAACTGGTCGACTTGAGTGCAGAAGAGGGAAGTGGAATTCCATGTGTAGCGGTGAAATGCGTAGAGATATGGAGGAACACCAGTGGCGAAGGCGGCTTCCTGGTCTGTAACTGACACTGAGGCGCGAAAGCGTGGGGAGCAAACAGGATTAGATACCCTGGTAGTCCACGCCGTAAACGATGAGTGCTAAGTGTTGGgggTCGAACCTCAGTGCTGAAGTTAACGCATTAAGCACTCCGCCTGGGGAGTACGGTCGCAAGACTGAAACTCAAATGAATTGACGG

>OTU89

TAATACGGAGGATCCGAGCGTTATCCGGATTTATTGGGTTTAAAGGGAGCGTAGGCGGGTTGTTAAGTCAGTTGTGAAAGTTTGCGGCTCAACCGTAAAATTGCAGTTGATACTGGCGACCTTGAGTGCAACAGAGGTAGGCGGAATTCGTGGTGTAGCGGTGAAATGCTTAGATATCACGAAGAACTCCGATTGCGAAGGCAGCTTACTGGATTGTAACTGACGCTGATGCTCGAAAGTGTGGGTATCAAACAGGATTAGATACCCTGGTAGTCCAcacaGTAAACGATGAATACTCGCTGTTGGCGATATACGGTCAGCGGCCAAGCGAAAGCATTAAGTATTCCACCTGGGGAGTACGCCGGCAACGGTGAAACTCAAATGAATTGACGGG

>OTU90

TAATACGGAGGATGCAAGCGTTATCCGGATTTATTGGGTTTAAAGGGTACGTAGGCGGAAAATTAAGTCAGTAGTGAAATCCCGCAGCTTAACTGTGGAACTGTTATTGATACTGGTTTTCTTGAATATAGTTGAGGTAGGCGGAATGTGTAATGTAGCGGTGAAATGCTTAGATATTACACAGAACACCGATTGCGAAGGCAGCTTACTAAGCTATGATTGACGCTGAGGTACGAAAGCGTGGGGAGCGAACAGGATTAGATACCCTGGTAGTCCACGCCGTAAACGATGATCACTCGTTGTTGGCAATACATCGTCAGCGACTGAGCGAAAGCATTAAGTGATCCACCTGGGGAGTACGCTCGCAAGAGTGAAACTTAAAGGAATTGACGG

>OTU91

TAATACGTAGGTGGCGAGCGTTGTCCGGAATTATTGGGCGTAAAGAGCATGTAGGCGGCTTAATAAGTCGAGCGTGAAAATGCGGGGCTCAACCCCGTATGGCGCTGGAAACTGTTAGGCTTGAGTGCAGGAGAGGAAAGGGGAATTCCCAGTGTAGCGGTGAAATGCGTAGATATTGGGAGGAACACCAGTGGCGAAGGCGCCTTTCTGGACTGTGTCTGACGCTGAGATGCGAAAGCCAGGGTAGCGAACGGGATTAGATACCCCGGTAGTCCTGGCCGTAAACGATGGGTACTAGGTGTAGGAGGTATCGACCCCTTCTGTGCCGGAGTTAACGCAATAAGTACCCCGCCTGGGGAGTACGGCCGCAAGGTTGAAACTTAAATGAATTGACGG

>OTU92

TAATACGTAGGTGGCAAGCGTTATCCGGATTTATTGGGCGTAAAGagagTGCAGGCGGTTTTCTAAGTCTGATGTGAAAGCCTTCGGCTTAACCGGAGAAGTGCATCGGAAACTGGATAACTTGAGTGCAGAAGAGGGTAGTGGAACTCCATGTGTAGCGGTGGAATGCGTAGATatatGGAAGAACACCAGTGGCGAAGGCGGCTACCTGGTCTGCAACTGACGCTGAGACTCGAAAGCATGGGTAGCGAACAGGATTAGATACCCTGGTAGTCCATGCCGTAAACGATGAGTGCTAGGTGTTGGAGGGTTTCCGCCCTTCAGTGCCGGAGCTAACGCATTAAGCACTCCGCCTGGGGAGTACGACCGCAAGGTTGAAACTTAAATGAATTGACGG

>OTU93

TAATACGAAGGgggCTAGCGTTGTTCGGAATTACTGGGCGTAAAGCgcgcgTAGGCGGTGATCTTTGTCAGAGGTGAAATCCCGAGGCTCAACTTCGGAATTGCCTTTGAAACGGGATCGCTTGAGTCCGAgagagGATGGCGGAATTCCTAGTGTAGAGGTGAAATTCGTAGATATTAGGAAGAACACCGGTGGCGAAGGCGGCCATCTGGCTCGGTACTGACGCTCAGGCGCGAAAGCGTGGGGAGCAAACAGGATTAGATACCCTGGTAGTCCACGCCGTAAACTATGAGTGCTAGTTGTTGGGAGGTTTACCTCTCAGTGACGCAGCTAACGCGTTAAGCACTCCGCCTGGGGAGTACGGTCGCAAGATTAAAACTCAAAGGAATTGACGG

>OTU94

TAATACGGAGGATCCGAGCGTTATCCGGATTTATTGGGTTTAAAGGGTGCGTAGGCGGCCTTTTAAGTCAGCGGTGAAAGTCTGTGGCTCAACCATAGAATTGCCGTTGAAACTGGggggCTTGAGTATGTTTGAGGCAGGCGGAATGCGTGGTGTAGCGGTGAAATGCTTAGATATCACGCAGAACCCCGATTGCGAAGGCAGCCTGCCAAGCCATGACTGACGCTGATGCACGAAAGCGTGGGGATCAAACAGGATTAGATACCCTGGTAGTCCACGCAGTAAACGATGATCACTAGCTGTTTGCGATACACTGTAAGCGGCACAGCGAAAGCGTTAAGTGATCCACCTGGGGAGTACGCCGGCAACGGTGAAACTTAAATGAATTGACGG

>OTU95

TAATACGTAGGTGGCGAGCGTTATCCGGAATTATTGGGCGTAAAGCgcgcgcAGGTGGTTTCTTAAGTCTGATGTGAAAGCCTACGGCTTAACCGTAGAATTGCATTGGAAACTGGgggACTTGAGTGCAGAAGAGGAAAGTGGAATTCCACGTGTAGCGGTGAAATGCGTAGAGATGTGGAGGAACACCGGTGGCGAAGGCGACTTTCTGGTCTGCAACTGACACTGAGGCGCGAAAGCGTGGGGAGCAAACAGGATTAGATACCCTGGTAGTCCACGCCGTAAACGATGAGTGCTAAGTGTTAGAGGGTTTCCGCCCTTTAGTGCTGAAGTTAACGCATTAAGCACTCCGCCTGGGGAGTACGGCCGCAAGGCTGAAACTCAAAGGAATTGACGG

>OTU96

TAATACGAAGGgggCTAGCGTTGCTCGGAATTACTGGGCGTAAAGGGAGCGTAGGCGGACATTTAAGTCAGGGGTGAAATCCCGGGGCTCAACCTCGGAACTGCCTTTGATACTGGGTGTCTTGAGTGTGATAGAGGTATGTGGAACTCCGAGTGTAGAGGTGAAATTCGTAGATATTCGGAAGAACACCAGTGGCGAAGGCGACATACTGGATCATTACTGACGCTGAGGCTCGAAAGCGTGGGGAGCAAACAGGATTAGATACCCTGGTAGTCCACGCCGTAAACGATGATTGCTAGTTGTCGGGGTGTTTACACCTCGGTGACGCAGCTAACGCATTAAGCAATCCGCCTGGGGAGTACGGTCGCAAGATTAAAACTCAAATGAATTGACGG

>OTU97

TAATACGTAGGGCGCAAGCGTTATCCGGAATTATTGGGCGTAAAGAGCTCGTAGGCGGTCTGTCGCGTCTGCTGTGAAAACCCGAGGCTCAACCTCGGGCCTGCAGTGGGTACGGGCAGACTAGAGTGCGGTAGGGGAGATTGGAATTCCTGGTGTAGCGGTGGAATGCGCAGATATCAGGAGGAACACCGATGGCGAAGGCAGATCTCTGGGCCGTAACTGACGCTGAGGAGCGAAAGGGTGGGGAGCAAACAGGCTTAGATACCCTGGTAGTCCACCCCGTAAACGTTGGGAACTAGTTGTGGgggCCTTTCCACGGTCTCCGTGACGCAGCTAACGCATTAAGTTCCCCGCCTGGGGAGTACGGCCGCAAGGCTAAAACTCAAATGAATTGACGG

>OTU98

TAATACGGAGGGTGCAAGCGTTAATCGGAATTACTGGGCGTAAAGCGCACGCAGGCGGTTGGATAAGTTAGATGTGAAAGCCCCGGGCTCAACCTGGGAATTGCATTTAAAACTGTCCAGCTAGAGTCTTGTAGAGGggggTAGAATTCCAGGTGTAGCGGTGAAATGCGTAGAGATCTGGAGGAATACCGGTGGCGAAGGCGGCCcccTGGACAAAGACTGACGCTCAGGTGCGAAAGCGTGGGGAGCAAACAGGATTAGATACCCTGGTAGTCCACGCCGTAAACGATGTCGATTTGGAGGCTGTGTCCTTGAGACGTGGCTTCCGGAGCTAACGCGTTAAATCGACCGCCTGGGGAGTACGGCCGCAAGGTTAAAACTCAAATGAATTGACGG

>OTU99

TAATACGTAGGGAGCGAGCGTTGTCCGGATTTACTGGGTGTAAAGGGTGCGTAGGCGGATCTGCAAGTCAGTAGTGAAATCCCAGGGCTTAACCCTGGAACTGCTATTGAAACTGTGGGTCTTGAGTGAGGTAGAGGCAGGCGGAATTCCCGGTGTAGCGGTGAAATGCGTAGAGATCGGGAGGAACACCAGTGGCGAAGGCGGCCTGCTGGGCCTTAACTGACGCTGAGGCACGAAAGCATGGGTAGCAAACAGGATTAGATACCCTGGTAGTCCATGCCGTAAACGATGATTACTAGGTGTGGGTGGTCTGACCCCATCCGTGCCGGAGTTAACACAATAAGTAATCCACCTGGGGAGTACGGCCGCAAGGTTGAAACTTAAAGGAATTGACGG

>OTU100

TAATACGTAGGTCCCGAGCGTTATCCGGATTTATTGGGCGTAAAGCGAGCGCAGGCGGTTAGATAAGTCTGAAGTTAAAGGCTGTGGCTTAACCATAGTACGCTTTGGAAACTGTTTAACTTGAGTGCAAGAGGGGAGAGTGGAATTCCATGTGTAGCGGTGAAATGCGTAGATatatGGAGGAACACCGGTGGCGAAAGCGGCTctctGGCTTGTAACTGACGCTGAGGCTCGAAAGCGTGGGGAGCAAACAGGATTAGATACCCTGGTAGTCCACGCCGTAAACGATGAGTGCTAGGTGTTAGACCCTTTCCGGGGTTTAGTGCCGCAGCTAACGCATTAAGCACTCCGCCTGGGGAGTACGACCGCAAGGTTGAAACTTAAATGAATTGACGGT

>OTU101

TAATACGTAGGgggCAAGCGTTATCCGGAATTACTGGGTGTAAAGGGTGCGTAGGCGGCGATTTAAGTCAGATGTGAAAACTCAGGGCTCAACCTTGAGACTGCATCTGAAACTGAGTTGCTAGAGTGCAGGAGAGGAAAGCGGAATTCCGAGTGTAGCGGTGAAATGCGTAGAGATTCGGAGGAACACCAGTAGCGAAGGCGGCTTTCTGGACTGTAACTGACGCTGAGGCACGAAAGCGTGGGGAGCGAACAGGATTAGATACCCTGGTAGTCCACGCCGTAAACGATGAATACTAGGTGTCGGGGCTTACGGGTCTCGGTGCCGCAGCTAACGCATTAAGTATTCCACCTGGGAAGTACGACCGCAAGGTTGAAACTTAAATGAATTGACGGG

>OTU102

TAATACGGAGGATGCAAGCGTTATCCGGAATCATTGGGCGTAAAGCGTCCGCAGGTGGCTGTTCAAGTCTGCTGTTAAAGACAGAAGCTCAACTTCTGAACAGCAGTGGAAACTGGACAGCTAGAGTACGGTAGGGGTTGAGGGAATTCCCAGTGTAGCGGTGAAATGCGTAGATATTGGGAAGAACACCAGTGGCGAAGGCGCTCAACTGGGCCGTAACTGACACTCAGGGACGAAAGCTAGGGTAGCGAAAGGGATTAGATACCCCTGTAGTCTTAGCTGTAAACGATGAACACTAGGCGTTGCTTGTATCGACCCGAGCAGTGCCGTAGCCAACGCGTTAAGTGTTCCGCCTGGGGAGTACGCACGCAAGTGTGAAACTTAAATGAATTGACGG

>OTU103

TAATACGTAGGGTGCGAGCGTTGTCCGGAATTACTGGGCGTAAAGAGCTCGTAGGTGGTTTGTTGCGTCGTCTGTGAAATTCCGGGGCTTAACTTCGGGGTGGCAGGCGATACGGGCATAACTAGAGTGCTGTAGGGGAGACTGGAATTCCTGGTGTAGCGGTGGAATGCGCAGATATCAGGAGGAACACCGATGGCGAAGGCAGGTCTCTGGGCAGTAACTGACGCTGAGGAGCGAAAGCATGGGGAGCGAACAGGATTAGATACCCTGGTAGTCCATGCTGTAAACGGTGGGCGTTAGGTGTGGGGACCGGTTTGGTTTCTGTGCCGTAGCTAACGCATTAAGCGCCCCGCCTGGGGAGTACGGCCGCAAGGCTAAAACTTAAATGAATTGACGG

>OTU104

TAATACGGAGGATCCGAGCGTTATCCGGATTTATTGGGTTTAAAGGGAGCGTAGATGGATGTTTAAGTCAGTTGTGAAAGTTTGCGGCTCAACCGTAAAATTGCAGTTGATACTGGATATCTTGAGTGCAGTTGAGGCAGGCGGAATTCGTGGTGTAGCGGTGAAATGCTTAGATATCACGAAGAACTCCGATTGCGAAGGCAGCCTGCTAAGCTGCAACTGACATTGAGGCTCGAAAGTGTGGGTATCAAACAGGATTAGATACCCTGGTAGTCCACACGGTAAACGATGAATACTCGCTGTTTGCGATATACTGCAAGCGGCCAAGCGAAAGCGTTAAGTATTCCACCTGGGGAGTACGCCGGCAACGGTGAAACTTAAATGAATTGACGGG

>OTU105

TAATACGTAGGTCCCGAGCGTTGTCCGGATTTATTGGGCGTAAAGCGAGCGCAGGTGGTTTAATAAGTCTGATGTAAAAGGCAGTGGCTCAACCATTGtgtgCATTGGAAACTGTTAGACTTGAGTGCAGTAGAGGAGAGTGGAATTCCATGTGTAGCGGTGAAATGCGTAGATatatGGAGGAACACCGGTGGCGAAAGCGGCTctctGGACTGTCACTGACACTGAGGCTCGAAAGCGTGGGTAGCAAACAGGATTAGATACCCTGGTAGTCCACGCCGTAAACGATGAGTGCTAGTTGTTTGGGGCTATCCAGCCCTAAGTGACGCAGCAAACGCATTAAGCACTCCGCCTGGGGAGTACGACCGCAAGGTTGAAACTTAAATGAATTGACGG

>OTU106

TAATACGTAGGTGGCAAGCGTTATCCGGATTTACTGGGTGTAAAGGGCGTGTAGGCGGGATTGCAAGTCAGATGTGAAAACTGGgggCTCAACCTCCAGCCTGCATTTGAAACTGTAGTTCTTGAGTGCTGGAGAGGCAATCGGAATTCCGTgtgtAGCGGTGAAATGCGTAGATATACGGAGGAACACCAGTGGCGAAGGCGGATTGCTGGACAGTAACTGACGCTGAGGCGCGAAAGCGTGGGGAGCAAACAGGATTAGATACCCTGGTAGTCCACGCCGTAAACGATGGATACTAGGTGTGGggggTCTGACCcccTCCGTGCCGCAGTTAACACAATAAGTATCCCACCTGGGGAGTACGATCGCAAGGTTGAAACTTAAATGAATTGACGG

>OTU107

TAATACGTAGGTGGCAAGCGTTGTCCGGATTTATTGGGCGTAAAGGGAACGCAGGCGGTCTTTTAAGTCTGATGTGAAAGCCTTCGGCTTAACCGGAGTAGTGCATTGGAAACTGGAAGACTTGAGTGCAGAAGAGGAGAGTGGAACTCCATGTGTAGCGGTGAAATGCGTAGATatatGGAAGAACACCAGTGGCGAAAGCGGCTctctGGTCTGTAACTGACGCTGAGGTTCGAAAGCGTGGGTAGCAAACAGGATTAGATACCCTGGTAGTCCACGCCGTAAACGATGAATGCTAGGTGTTGGAGGGTTTCCGCCCTTCAGTGCCGCAGCTAACGCAATAAGCATTCCGCCTGGGGAGTACGACCGCAAGGTTGAAACTTAAATGAATTGACGGG

>OTU108

TAATACGTAGGTGGCGAGCGTTATCCGGAATTATTGGGCGTAAAGGGTGCGTAGGCGGCCTGTTAAGTAAGTGGTTAAATTGTTGGGCTCAACCCAATCCAGCCACTTAAACTGGCAGGCTAGAGTATTGGAGAGGCAAGTGGAATTCCATGTGTAGCGGTAAAATGCGTAGATatatGGAGGAACACCAGTGGCGAAGGCGGCTTGCTAGCCAAAGACTGACGCTCATGCACGAAAGCGTGGGGAGCAAATAGGATTAGATACCCTAGTAGTCCACGCCGTAAACGATGAATACTAAGTATTGGGGAAACTCAGTGCTGCAGCTAACGCAATAAGTATTCCGCCTGTGGAGTATGCACGCAAGTGTGAAACTCAAAGGAATTGACGG

>OTU109

TAATACGTAGGGCGCGAGCGTTGTCCGGAATTATTGGGCGTAAAGAGCTCGTAGGCGGCTGGTCGCGTCTGTCGTGAAATCCCCTGGCTTAACTGGgggCTTGCGGTGGGTACGGGCCGGCTTGAGTGCGGTAGGGGAGACTGGAATTCCTGGTGTAGCGGTGGAATGCGCAGATATCAGGAGGAACACCGGTGGCGAAGGCGGGTCTCTGGGCCGTTACTGACGCTGAGGAGCGAAAGCGTGGGGAGCGAACAGGATTAGATACCCTGGTAGTCCACGCCGTAAACGTTGGGCACTAGGTGTGGgggCTCTTTCCGGGGTTTCCGCGCCGTAGCTAACGCATTAAGTGCCCCGCCTGGGGAGTACGGCCGCAAGGCTAAAACTTAAAGGAATTGACGG

>OTU110

TAATACGTAGGGTGCAAGCGTTGTCCGGAATTACTGGGCGTAAAGAGCTCGTAGGCGGTTTGTCGCGTCGTCTGTGAAATTCTGCAGCTTAACTGCAGGCGTGCAGGCGATACGGGCAGACTTGAGTACTACAGGGGAGACTGGAATTCCTGGTGTAGCGGTGAAATGCGCAGATATCAGGAGGAACACCGGTGGCGAAGGCGGGTCTCTGGGTAGTAACTGACGCTGAGGAGCGAAAGCGTGGGTAGCGAACAGGATTAGATACCCTGGTAGTCCACGCCGTAAACGGTGGGTACTAGGTGTGGGTTTCATTTCACGGAATCCGTGCCGTAGCTAACGCATTAAGTACCCCGCCTGGGGAGTACGGCCGCAAGGCTAAAACTCAAATGAATTGACGG

>OTU111

TAATACGTAGGGCGCGAGCGTTGTCCGGAATTATTGGGCGTAAAGGGCTTGTAGGCGGCTGGTCGCGTCTGCCGTGAAATCCTCTGGCTCAGCTGGgggCGTGCGGTGGGTACGGGCTGGCTTGAGTGCGGTAGGGGAGGCTGGAACTCCTGGTGTAGCGGTGGAATGCGCAGATATCAGGAGGAACACCGGTGGCGAAGGCGGGTCTCTGGGCCGTTACTGACGCTGAGGAGCGAAAGCGTGGGGAGCGAACAGGATTAGATACCCTGGTAGTCCATGCTGTAAACGTTGGGCACTAGGTGTGGggggTCTTTCCGGGCCGTCCGCGCCGTAGCTAACGCATTAAGTGCCCCGCCTGGGGAGTACGGCCGCAAGGCTAAAACTCAAATGAATTGACGG

>OTU112

TAATACGAAGGgggCTAGCGTTGTTCGGATTTACTGGGCGTAAAGCGCACGTAGGCGGATTtttAAGTCAGGGGTGAAATCCCGGGGCTCAACCCCGGAACTGCCTTTGATACTGGAAGTCTTGAGTATGGTAGAGGTGAGTGGAATTCCGAGTGTAGAGGTGAAATTCGTAGATATTCGGAGGAACACCAGTGGCGAAGGCGGCTCACTGGACCATTACTGACGCTGAGGTGCGAAAGCGTGGGGAGCAAACAGGATTAGATACCCTGGTAGTCCACGCCGTAAACGATGAATGTTAGCCGTCGGGGAGTTTACTCTTCGGTGGCGCAGCTAACGCATTAAACATTCCGCCTGGGGAGTACGGTCGCAAGATTAAAACTTAAAGGAATTGACGG

>OTU113

TAATACATAGGGGACGAGCGTTATCCGGAATAATTGGGCGTAAAGGGTGCGTAGGCGGTgtgtTAAGTTCAAGGTTTAATCGTAGGGCTCAACCCTATATCGCTTTGAAAACTGGCACACTAGAGTAAGATAGAGGCAAGTGGAATTCCATGTGTAGCGGTAAAATGCGTAAATatatGGCAGAACACCAGAGGCGAAGGCGGCTTGCTGGGTCTTCACTGACGCTGAGGCACGAAAGCGTGGGGAGCAAACAGGATTAGATACCCTGGTAGTCCACGCCGTAAACGATGAGCACTAAAAGTCGGGATTACTCGGTTTTCAAGTTAACGCGATAAGTGCTCCGCCTGAGTAGTACGTACGCAAGTATAAAACTCAAATGAATTGACGG

>OTU114

TAATACGGAGGATGCGAGCGTTATCCGGATTTATTGGGTTTAAAGGGTGCGTAGGTTGTTCGGTAAGTCAGCGGTGAAACCTGAGCGCTCAACGTTCAGCCTGCCGTTGAAACTGCCGGGCTTGAGTTCAGTGGCGGCAGGCGGAATTCGTGGTGTAGCGGTGAAATGCATAGATATCACGAGGAACTCCGATTGCGAAGGCAGCTTGCCATACTGCGACTGACACTGAAGCACGAAGGCGTGGGTATCAAACAGGATTAGATACCCTGGTAGTCCACGCAGTAAACGATGATTACTAGGAGTTTGCGATATACCGTCAAGCTTCCACAGCGAAAGCGTTAAGTAATCCACCTGGGGAGTACGCCGGCAACGGTGAAACTTAAATGAATTGACGG

>OTU115

TAATACGAAGGgggCTAGCGTTGCTCGGAATTACTGGGCGTAAAGCGCACGTAGGCGGCTCTTTAAGTCGGgggTGAAATCCTGGAGCTCAACTCCAGAACTGCCTTCGATACTGTAGAGCTTGAGTTCGGGAGAGGTGAGTGGAACTGCGAGTGTAGAGGTGAAATTCGTAGATATTCGCAAGAACACCAGTGGCGAAGGCGGCTCACTGGCCCGATACTGACGCTGAGGTGCGAAAGCGTGGGGAGCAAACAGGATTAGATACCCTGGTAGTCCACGCCGTAAACGATGAATGCTAGCCGTTGTCAGGCTTGCCTGTCAGTGGCGCAGCTAACGCATTAAGCATTCCGCCTGGGGAGTACGGTCGCAAGATTAAAACTCAAATGAATTGACGG

>OTU116

TAATACGTATGTCACAAGCGTTATCCGGATTTATTGGGCGTAAAGCGCGTCTAGGCGGTTAATTAAGTCTGATGTGAAAATGCGGAGCTCAACTCCGTATTGCGTTGGAAACTGGTTAACTAGAGTACTGGAGAGGTAAGCGGAACTACAAGTGTAGAGGTGAAATTCGTAGATATTTGTAGGAATGCCGATGGGGAAGCCAGCTTACTGGACAGATACTGACGCTAAAGCGCGAAAGCGTGGGTAGCAAACAGGATTAGATACCCTGGTAGTCCACGCTGTAAACGATGATTACTAGGTGTTGGgggTCGAACCTCAGCGCCCAAGCTAACGCGATAAGTAATCCGCCTGGGGAGTACGTACGCAAGTATGAAACTTAAAGGAATTGACGG

>OTU117

TAATACGTAGGGTGCGAGCGTTGTCCGGAATTATTGGGCGTAAAGAGCTTGTAGGCGGTTTGTCGCGTCTGCTGTGAAAATCCGGGGCTCAACCCCGGACTTGCAGTGGGTACGGGCAGACTAGAGTGTGGTAGGGGAGACTGGAATTCCTGGTGTAGCGGTGAAATGCGCAGATATCAGGAGGAACACCGATGGCGAAGGCAGGTCTCTGGGCCACTACTGACGCTGAGAAGCGAAAGCATGGGGAGCGAACAGGATTAGATACCCTGGTAGTCCATGCCGTAAACGTTGGGCGCTAGGTGTGGGACTCATTCCACGAGTTCCGTGCCGCAGCTAACGCATTAAGCGCCCCGCCTGGGGAGTACGGCCGCAAGGCTAAAACTTAAATGAATTGACGG

>OTU118

TAATACGGAGGGTGCAAGCGTTAATCGGAATTATTGGGCGTAAAGCgcgcgcAGGCGGATCAGTCAGTCTGTCTTAAAAGTTCGGGGCTTAACCCCGTGAGGGGATGGAAACTGCTGATCTAGAGTATCGGAGAGGAAAGTGGAATTCCTAGTGTAGCGGTGAAATGCGTAGATATTAGGAAGAACACCAGTGGCGAAGGCGACTTTCTGGACGAAAACTGACGCTGAGGCGCGAAAGCCAGGGGAGCGAACGGGATTAGATACCCCGGTAGTCCTGGCCGTAAACGATGGGTACTAGGTGTAGGAGGTATCGACCCCTTCTGTGCCGGAGTTAACGCAATAAGTACCCCGCCTGGGGAGTACGACCGCAAGGTTGAAACTCAAAGGAATTGACGG

>OTU119

TAATACAGAGGATGCAAGCGTTATCCGGAATGATTGGGCGTAAAGCGTCCGCAGGTGGTTTGATAAGTCTGCTGTTAAAGCGTGTGGCTCAACCACATACGGGCAGTGGAAACTATTCGACTAGAGTATGTTAGGGGTAGAGGGAATTCCCAGTGTAGCGGTGAAATGCGTAGAGATTGGGAAGAACACCGGTGGCGAAAGCGCTCTGCTAGGACAATACTGACACTGAGGGACGAAAGCTAGGGGAGCGAATGGGATTAGATACCCCAGTAGTCCTAGCCGTAAACGATGGATACTAGGCGTTGCCCGTATCGACCCGGGCATTGTCGTAGCTAACGCGTTAAGTATCCCGCCTGGGGAGTACGCACGCAAGTGTGAAACTCAAAGGAATTGACGG

>OTU120

TAATACGTAGGGTGCAAGCGTTAATCGGAATTACTGGGCGTAAAGCGTGCGCAGGCGGTTTCGTAAGTCTGTCGTGAAATCCCCGGGCTTAACCTGGGAATGGCGATGGAGACTGCGAGGCTAGAGTTTGGCAGAGGggggTAGAATTCCACGTGTAGCAGTGAAATGCGTAGAGATGTGGAGGAACACCGATGGCGAAGGCAGCCcccTGGGTCAAAACTGACGCTCATGCACGAAAGCGTGGGGAGCAAACAGGATTAGATACCCTGGTAGTCCACGCCCTAAACGATGTCTACTAGTTGTTGGGTCTTAATTGACTTAGTAACGCAGCTAACGCGTGAAGTAGACCGCCTGGGGAGTACGGTCGCAAGATTAAAACTTAAAGGAATTGACGG

>OTU121

TAATACGAAGGGTGCAAGCGTTACTCGGAATTACTGGGCGTAAAGCGTGCGTAGGTGGTTGTTTAAGTCTGTCGTGAAAGCCCTGGGCTCAACCTGGGAATTGCGATGGAAACTGGGCGACTAGAGTGTGGCAGAGGATAGTGGAATTCCTGGTGTAGCAGTGAAATGCGTAGAGATCAGGAGGAACATCCGTGGCGAAGGCGACTGTCTGGGCCAACACTGACACTGAGGCACGAAAGCGTGGGGAGCAAACAGGATTAGATACCCTGGTAGTCCACGCCCTAAACGATGCGAACTGGATGTTGGGTGCAATTTGGCACGCAGTATCGAAGCTAACGCGTTAAGTTCGCCGCCTGGGGAGTACGGTCGCAAGACTGAAACTTAAATGAATTGACGG

>OTU122

TAATACGTAGGgggCAAGCGTTATCCGGATTTACTGGGTGTAAAGGGAGCGTAGACGGGATAGCAAGTCTGAAGTGAAAGCCCGGGGCTCAACCCCGGGACTGCTTTGGAAACTGTTGACCTGGAGTGCTGGAGAGGCAAGTGGAATTCCTAGTGTAGCGGTGAAATGCGTAGATATTAGGAGGAACACCAGTGGCGAAGGCGGCTTGCTGGACAGTGACTGACGTTGAGGCTCGAAAGCGTGGGGAGCAAACAGGATTAGATACCCTGGTAGTCCACGCCGTAAACGATGACTACTAGGTGTCGGTGAGCAAAGCTCATCGGTGCCGCAGCCAACGCAATAAGTAGTCCACCTGGGGAGTACGTTCGCAAGAATGAAACTCAAAGGAATTGACGG

>OTU123

TAATACGTAGGTGGCAAGCGTTGTCCGGATTTATTGGGCGTAAAGGGAGCGCAGGTGGTTTCTTAAGTCTGATGTGAAAGCCCACGGCTTAACCGTGGAGGGTCATTGGAAACTGGGAAACTTGAGTACAGAAGAGGAATGTGGAACTCCATGTGTAGCGGTGGAATGCGTAGATatatGGAAGAACACCAGTGGCGAAGGCGACATTCTGGTCTGTTACTGACACTGAGGCTCGAAAGCGTGGGGAGCAAACAGGATTAGATACCCTGGTAGTCCACGCCGTAAACGATGAGTGCTAGGTGTTGGAGGGTTTCCGCCCTTCAGTGCCGCAGTTAACGCATTAAGCACTCCGCCTGGGGAGTACGACCGCAAGGTTGAAACTCAAATGAATTGACGG

>OTU124

TAATACGGAAGGTCCGGGCGTTATCCGGATTTATTGGGTTTAAAGGGAGCGCAGGCGGCCTTTTAAGCGTGACGTGAAATGTGGATGCTCAACATCTGAACTGCGTCGCGAACTGGAGGGCTTGAGTGGGTACGACGCCGGCGGAATTCGTGGTGTAGCGGTGAAATGCTTAGATATCACGAAGAACCCCGATTGCGAAGGCAGCCGGCGAGTCCTCAACTGACGCTAAAGCTCGAAAGTGCGGGTATCGAACAGGATTAGATACCCTGGTAGTCCGCACGGTAAACGATGGATGCCCGCTGTTGGCGATACAATGTCAGCGGCCAAGCGAAAGCGTTAAGCATCCCACCTGGGGAGTACGCCGGCAACGGTGAAACTTAAAGGAATTGACGG

>OTU125

TAATACGTAGGgggCTAGCGTTATCCGGATTTACTGGGCGTAAAGGGTGCGTAGGCGGTCTTTCAAGTCAGGAGTGAAAGGCTACGGCTCAACCGTAGTAAGCTCTTGAAACTGGGAGACTTGAGTGCAGGAGAGGAGAGTGGAATTCCTAGTGTAGCGGTGAAATGCGTAGATATTAGGAGGAACACCAGTTGCGAAGGCGGCTctctGGACTGTAACTGACGCTGAGGCACGAAAGCGTGGGGAGCAAACAGGATTAGATACCCTGGTAGTCCACGCTGTAAACGATGAGTACTAGGTGTCGGgggTTACCcccTTCGGTGCCGCAGCTAACGCATTAAGTACTCCGCCTGGGAAGTACGCTCGCAAGAGTGAAACTTAAATGAATTGACGGG

>OTU126

TAATACGGAGGGTGCAAGCGTTATCCGGATTTATTGGGTTTAAAGGGTCCGTAGGCGGATCTGTAAGTCAGTGGTGAAATCTCACAGCTTAACTGTGAAACTGCCATTGATACTGCAGGTCTTGAGTAAGGTAGAAGTGGCTGGAATAAGTAGTGTAGCGGTGAAATGCATAGATATTACTTAGAACACCAATTGCGAAGGCAGGTCACTATGTCTTAACTGACGCTGATGGACGAAAGCGTGGGGAGCGAACAGGATTAGATACCCTGGTAGTCCACGCCGTAAACGATGCTAACTCGTTtttGGGCTTTCGGGTTCAGAGACTAAGCGAAAGTGATAAGTTAGCCACCTGGGGAGTACGTTCGCAAGAATGAAACTTAAAGGAATTGACGG

>OTU127

TAATACGGAGGATGCGAGCGTTATCCGGATTTATTGGGTTTAAAGGGTGCGCAGGCGGGATTTTAAGTCAGCGGTGAAATTTTCAGGCTCAACCTGAACACTGCCGTTGAAACTGGGATTCTTGAGTATGGATGAAGTAGGCGGAATTCGTTGTGTAGCGGTGACATGCTTAGATATAACGAGGAACTCCGATTGCGTAGGCAGCTTACTAAGCCATAACTGACGCTCAAGCACGAAAGCGTGGGGATCAAACAGGATTAGATACCCTGGTAGTCCACGCCGTAAACGATGATTACTAGTTGTTTGCGATATACCGTAAGTGACTAAGCGAAAGCGATAAGTAATCCACCTGGGGAGTACGCCGGCAACGGTGAAACTTAAATGAATTGACGG

>OTU128

TAATACGGAGGGTGCAAGCGTTAATCGGAATTACTGGGCGTAAAGCGCACGCAGGCGGTCTGTCAAGTCGGATGTGAAATCCCCGGGCTCAACCTGGGAACTGCATTCGAAACTGGCAGGCTGGAGTCTTGTAGAGGggggTAGAATTCCAGGTGTAGCGGTGAAATGCGTAGAGATCTGGAGGAATACCGGTGGCGAAGGCGGCCcccTGGACAAAGACTGACGCTCAGGTGCGAAAGCGTGGGGAGCAAACAGGATTAGATACCCTGGTAGTCCACGCTGTAAACGATGTCGACTTGGAGGTTGTTCCCTTGAGGAGTGGCTTCCGGAGCTAACGCGTTAAGTCGACCGCCTGGGGAGTACGCCGGCAACGGTGAAACTCAAATGAATTGACGG

>OTU129

TAATACGAAGGgggCTAGCGTTGTTCGGAATTACTGGGCGTAAAGCGTGCGCAGGCGGCTTCTCCAGTCAGAGGTGAAAGCCCAGAGCTCAACTCTGGAATTGCCTTTGAAACTAGGAAGCTTGAGTACGGGAGAGGTGAGTGGAATTCCGAGTGTAGAGGTGAAATTCGTAGATATTCGGAAGAACACCAGTGGCGAAGGCGGCTCACTGGACCGTAACTGACGCTCATGCACGACAGCGTGGGGATCAAACAGGATTAGATACCCTGGTAGTCCACGCCGTAAACGATGATTGCTAGCCGTCGGGCAGCTTGCTGTTCGGTGGCGCAGCTAACGCATTAAGCAATCCGCCTGGGGAGTACGGCCGCAAGGTTGAAACTTAAATGAATTGACGG

>OTU130

TAATACGGAGGgggCTAGCGTTGTTCGGAATTACTGGGCGTAAAGCGCACGTAGGCGGATTGGCAAGTTGGgggTGAAATCCCAGGGCTCAACCCTGGAACTGCCTCCAAAACTTCCAGTCTTGAGGTCGAgagagGTGAGTGGAATTCCGAGTGTAGAGGTGAAATTCGTAGATATTCGGAGGAACACCAGTGGCGAAGGCGGCTCACTGGCTCGATACTGACGCTGAGGTGCGAAAGCGTGGGGAGCAAACAGGATTAGATACCCTGGTAGTCCACGCCGTAAACGATGAGAGCTAGACGTCGGGGAGCATGCTCTTCGGTGTCGCAGTTAACGCATTAAGCTCTCCGCCTGGGGAGTACGGCCGCAAGGTTAAAACTCAAATGAATTGACGG

>OTU131

TAATACGGAGGATGCAAGCGTTATCCGGAATCATTGGGTTTAAAGGGTCTGTAGGCGGGCAGATAAGTCAGAGGTGAAAGCGCTTAGCTCAACTAAGCAACTGCCTTTGAAACTGTTTGTCTTGAATGGTTGTGAAGTAGTTGGAATGTGTAGTGTAGCGGTGAAATGCTTAGATATTACACAGAACACCGATAGCGAAGGCATATTACTAACAATTAATTGACGCTGATGGACGAAAGCGTGGGGAGCGAACAGGATTAGATACCCTGGTAGTCCACGCTGTAAACGATGGATACTAGCTGTTTGGTTGTAAGACTGAGTGGCTAAGCGAAAGTGATAAGTATCCCACCTGGGGAGTACGTTCGCAAGAATGAAACTTAAATGAATTGACGG

>OTU132

TAATACGTAGGgggCAAGCGTTATCCGGAATTATTGGGCGTAAAGAGTGCGTAGGTGGTTACCTAAGCGCAAGGTTTAATTTAGAGGCTCAACCTCTACTTGCCTTGCGAACTGGGCTACTTGAGTGCAGGAGGGGAAAGCGGAATTCCTAGTGTAGCGGTGAAATGCGTAGATATTAGGAGGAACACCAGCGGCGAAGGCGGCTTTCTGGACTGTAACTGACACTGAGGCACGAAAGCGTGGGTAGCAAACAGGATTAGATACCCTGGTAGTCCACGCCGTAAACGATGAGCACTAGGTGTTGGGCCCGTTAGGGCTCAGTGCCGCAGTTAACGCAATAAGTGCTCCGCCTGGGGAGTACGCTCGCAAGAGTAAAACTTAAATGAATTGACGG

>OTU133

TAATACGTAGGTGGCGAGCGTTGTCCGGATTTACTGGGCGTAAAGAGCgcgcAGGCGGTCGATTTAGTCGTgtgtgAAAGCCcccGGCTCAACTGGGGAGGGTCACGCGATACTGATCGACTCGAAGGCAGGAGAGGGTAGTGGAATTCCCGGTGTAGTGGTGAAATGCGTAGATATCGGGAGGAACACCAGTGGCGAAGGCGACTACCTGGCCTGTTCTTGACGCTGAGGCGCGAAAGCTAGGGGAGCAAACGGGATTGGATACCCCGGTAGTCCTAGCCGTAAACGATGGACACTAGGTGTTGGTGGTATCAACCCCGCCAGTGCCGAAGCTAACGCATTAAGTGTCCCGCCTGGGGAGTACGGCCGCAAGGCTAAAACTCAAAGGAATTGACGG

>OTU134

TAATACGTAGGgggCTAGCGTTATCCGGATTTACTGGGCGTAAAGGGTGCGTAGGTGGTTTCTTAAGTCAGGAGTGAAAGGCTACGGCTTAACCGTAGTAAGCTCTTGAAACTGGGAAACTTGAGTGCAGGAGAGGAAAGTGGAATTCCTAGTGTAGCGGTGAAATGCGTAGATATTAGGAGGAACACCAGTAGCGAAGGCGGCTTTCTGGACTGTAACTGACACTGAGGCACGAAAGCGTGGGGAGCAAACAGGATTAGATACCCTGGTAGTCCACGCCGTAAACGATGAGTACTAGGTGTCGGgggTTACCccccTCGGTGCCGCAGCTAACGCATTAAGTACTCCGCCTGGGGAGTACGCTCGCAAGAGTGAAACTTAAAGGAATTGACGG

>OTU135

TAATACGGAGGgggCTAGCGTTGTTCGGAATTACTGGGCGTAAAGCGCAAGTAGGCGGAACAGAAAGTCAGAGGTGAAATCCCAGGGCTCAACCTTGGAACTGCCTTTGAAACTCCTGTTCTTGAGGTCGAgagagGTGAGTGGAATTCCGAGTGTAGAGGTGAAATTCGTAGATATTCGGAGGAACACCAGTGGCGAAGGCGGCTCACTGGCTCGATACTGACGCTGAGGTGCGAAAGCGTGGGGAGCAAACAGGATTAGATACCCTGGTAGTCAACGCCGTAAACGATGAATGCCAGTCGTCGGGTAGCATGCTATTCGGTGACacacCTAACGGATTAAGCATTCCGCCTGGGGAGTACGGCCGCAAGGTTAAAACTCAAATGAATTGACGG

>OTU136

TAATACGGAGGGTGCAAGCGTTATCCGGATTTATTGGGTTTAAAGGGTCCGTAGGCGGACTCGTAAGTCAGTGGTGAAATCTCATAGCTTAACTATGAAACTGCCATTGATACTGCGGGTCTTGAGTAAGGTAGAGGTAGCTGGAATAAGTAGTGTAGCGGTGAAATGCATAGATATTACTTAGAACACCAATTGCGAAGGCAGGTTACCATGTCTTAACTGACGCTGATGGACGAAAGCGTGGGGAGCGAACAGGATTAGATACCCTGGTAGTCCACGCCGTAAACGATGCTAACTCGTTtttGGGTTTTCGGATTCAGAGACTAAGCGAAAGTGATAAGTTAGCCACCTGGGGAGTACGAACGCAAGTTTGAAACTCAAAGGAATTGACGG

>OTU137

TCATACGTAGGATCCGAGCGTTATCCGGAGTGACTGGGCGTAAAGAGTTGCGTAGGTGGTTCGATAAGCGAATAGTGAAATCTGGTGGCTCAACCATTCAGACTATTATTCGAACTGTTGAACTCGAGAGCAGAAGAGGTAGCTGGAATTTCTAGTGTAGGAGTGAAATCCGTAGATATTAGAAGGAACACCGATGGCGTAGGCAGGCTACTGGGCTGTTTCTGACACTGAGGCACGAAAGCGTGGGGAGCGAACCGGATTAGATACCCGGGTAGTCCACGCCGTAAACGATGGATACTAGCTGTTTGGGGTATCGACCcccTGAGTAGCGAAGCTAACGCGTTAAGTATCCCGCCTGTGGAGTACGATCGCAAGATTAAAACTTAAATGAATTGACGG

>OTU138

TAATACGAAGGgggCTAGCGTTGCTCGGAATCACTGGGCGTAAAGGGCGCGTAGGCGGCTGATTTAGTCGAGGGTGAAAGCCCGTGGCTCAACCACGGAATGGCCTTCGATACTGATTGGCTTGAGACCGGAAGAGGACAGCGGAACTGCGAGTGTAGAGGTGAAATTCGTAGATATTCGCAAGAACACCAGTGGCGAAGGCGGCTGTCTGGTCCGGTTCTGACGCTGAGGCGCGAAAGCGTGGGGAGCAAACAGGATTAGATACCCTGGTAGTCCACGCTGTAAACGATGAATGCTAGCCGTTGGGGTGCATGCACCTCAGTGGCGCCGCTAACGCTTTAAGCATTCCGCCTGGGGAGTACGGTCGCAAGATTAAAACTTAAATGAATTGACGG

>OTU139

TAATACGTAGGGCGCGAGCGTTGTCCGGAATTATTGGGCGTAAAGAGCTTGTAGGCGGTTTGTCGCGTCTGCTGTGAAAGGCCGGGGCTTAACTCCGTGTATTGCAGTGGGTACGGGCAGACTAGAGTGCAGTAGGGGAGACTGGAACTCCTGGTGTAGCGGTGGAATGCGCAGATATCAGGAAGAACACCGATGGCGAAGGCAGGTCTCTGGGCTGTAACTGACGCTGAGAAGCGAAAGCATGGGGAGCGAACAGGATTAGATACCCTGGTAGTCCATGCCGTAAACGTTGGGCACTAGGTGTGGgggACATTCCACGTTTTCCGCGCCGTAGCTAACGCATTAAGTGCCCCGCCTGGGGAGTACGGCCGCAAGGCTAAAACTCAAATGAATTGACGG

>OTU140

TAATACGTAGGGTGCGAGCGTTGTCCGGAATTACTGGGCGTAAAGAGCTCGTAGGTGGTTTGTCGCGTCGTCTGTGAAAGTCCAGCGCTTAACGCTGGGATTGCAGGCGATACGGGCTTACTTGAGTGCTGTAGGGGAGACTGGAATTCCTGGTGTAGCGGTGAAATGCGCAGATATCAGGAGGAACACCAATGGCGAAGGCAGGTCTCTGGGCAGTCACTGACACTGAGGAGCGAAAGCATGGGTAGCCAACAGGATTAGATACCCTGGTAGTCCATGCCGTAAACGGTGGGCGCTAGGTGTGGGGACCTTCCACGGTTCCTGCGCCGTAGCTAACGCATTAAGCGCCCCGCCTGGGGAGTACGGCCGCAAGGCTAAAACTCAAATGAATTGACGG

>OTU141

TAATACGGAAGGGGCGAGCGTTGTTCGGAATTATTGGGCGTAAAGGGCGCGTAGGTGGTTCTGTATGTCTACCGTGAAAGGGTACAGCTCAACTGTAGCGCGTCGGTAGAAACTGCAGGACTTGAGGCTGGGAGGGGCTGGTGGAATTCCCTGTGTAGCGGTGAAATGCGTAGAGATGGgggAGAACACTCGTGGCGAAGGCGGCCAGCTAGACCAGTTCTGACACTGAGGCGCGACAGCGTGGGGAGCAAACAGGATTAGATACCCTGGTAGTCCACGCTGTAAACGATGGGCACTAGGTGCCGGCGGTCTTGATCCCGTCGGTGCCGGCGCTAACGCAGTAAGTGCCCCGCCTGGGGAGTACGGTCGCAAGGCTGAAACTTAAATGAATTGACGG

>OTU142

TAATACGGAGGgggCTAGCGTTGTTCGGAATTACTGGGCGTAAAGCGCACGTAGGCGGACTGGAAAGTCAGAGGTGAAATCCCAGGGCTCAACCTTGGAACTGCCTTTGAAACTATCAGTCTGGAGTTCGAgagagGTGAGTGGAATTCCGAGTGTAGAGGTGAAATTCGTAGATATTCGGAGGAACACCAGTGGCGAAGGCGGCTCACTGGCTCGATACTGACGCTGAGGTGCGAAAGCGTGGGGAGCAAACAGGATTAGATACCCTGGTAGTCCACGCCGTAAACGATGAATGCCAGACGTCGGCAAGCATGCTTGTCGGTGTCACACCTAACGGATTAAGCATTCCGCCTGGGGAGTACGGTCGCAAGATTAAAACTTAAATGAATTGACGG

>OTU143

TAATACAGAGGGTGCGAGCGTTGTTCGGAATTACTGGGCGTAAAGCgcgcgcAGGCGGGTCCTGTAAGTCGGAAGTGAAATTTCGGAGCTCAACTCCGAAGCTGCTTCTGATACTGCGGATCTAGGGACTGGTAGAGGCTGGTAGAATTACAGGTGTAGCGGTGGAATGCGTAGATATCTGTAAGAATACCCGTGGCGAAGGCGGCCAGCTGGGCCAGGTCCGACGCTGAGGCGCGAAAGCGTGGGGAGCAAACAGGATTAGATACCCTGGTAGTCCACGCCGTAAACGATGGGCACTAGGTGCCGGggggAGCGACCCCTTCGGTGCCGCAGCTAACGCGATAAGTGCCCCGCCTGGGGAGTACGGCCGCAAGGCTGAAACTTAAAGGAATTGACGG

>OTU144

TAATACGTAGGGTGCAAGCGTTAATCGGAATTATTGGGCGTAAAGCGAGTGCAGACGGTTACTTAAGCCAGATGTGAAATCCCCAAGCTTAACTTGGGACGTGCATTTGGAACTGGGTGACTAGAGTgtgtCAGAGGGAGGTAGAATTCCACATGTAGCGGTGGAATGCGTAGAGATGTGGAGGAATACCGATGGCGAAGGCAGCCTCCTGGGATAACACTGACGTTGAGGCTCGAAAGCGTGGGGAGCAAACAGGATTAGATACCCTGGTAGTCCACGCCCTAAACGATGACAATTAGCTGTTGGGCTTTGAAAGGCTTAGTAGCGAAGCTAACGCGAGAAATTGTCCGCCTGGGGAGTACGGTCGCAAGATTAAAACTTAAAGGAATTGACGG

>OTU145

TAATACGTATGGGGCAAGCGTTATCCGGATTTACTGGGTGTAAAGGGAGCGTAGGCGGCAGTACAAGTCGGGAGTGAAAACTCGGGGCTCAACCCCGAGACTGCTCTCGAAACTGTACAGCTAGAGTGCAGGATGGGCAGGCGGAATTCCTGGTGTAGCGGTGAAATGCGTAGATATCAGGAGGAACACCGGTGGCGAAGGCGGCCTGCTGGACTGTAACTGACGCTCAGGCTCGAAAGCGTGGGGAGCGAACAGGATTAGATACCCTGGTAGTCCACGCCGTAAACGATGAATACTAGGTGTCGGGGTCCTATAAGGACTTCGGTGCCGAAGCAAACGCATTAAGTATTCCACCTGGGGAGTACGTTCGCAAGAATGAAACTCAAAGGAATTGACGG

>OTU146

TAATACAGAGGGTGCGAGCGTTAATCGGATTTACTGGGCGTAAAGCGTGCGTAGGCGGCTTCTTAAGTCGGATGTGAAATCCCTGAGCTTAACTTAGGAATTGCATTCGATACTGGGAAGCTAGAGTATGGGAGAGGATGGTAGAATTCCAGGTGTAGCGGTGAAATGCGTAGAGATCTGGAGGAATACCGATGGCGAAGGCAGCCATCTGGCCTAATACTGACGCTGAGGTACGAAAGCATGGGGAGCAAACAGGATTAGATACCCTGGTAGTCCATGCCGTAAACGATGTCTACTAGCCGTTGGGGCCTTTGAGGCTTTAGTGGCGCAGCTAACGCGATAAGTAGACCGCCTGGGGAGTACGGTCGCAAGACTAAAACTTAAATGAATTGACGG

>OTU147

TAATACGTAGGgggCAAGCGTTATCCGGATTTACTGGGTGTAAAGGGAGCGTAGACGGCACTGCAAGTCTGGAGTGAAAGCCCGGGGCTCAACCCCGGGACTGCTTTGGAAACTGTGGTGCTGGAGTGCAGGAGAGGTAAGTGGAATTCCTAGTGTAGCGGTGAAATGCGTAGATATTAGGAGGAACACCAGTGGCGAAGGCGGCTTACTGGACTGTAACTGACGTTGAGGCTCGAAAGCGTGGGGAGCAAACAGGATTAGATACCCTGGTAGTCCACGCCGTAAACGATGAATACTAGGTGTTGGGGAGCAAAGCTCTTCGGTGCCGCCGCTAACGCAATAAGTATTCCACCTGGGGAGTACGTTCGCAAGAATGAAACTTAAAGGAATTGACGGG

>OTU148

TAATACGTAGGgggCAAGCGTTATCCGGATTTACTGGGTGTAAAGGGAGCGTAGACGGCATGGCAAGCCAGATGTGAAAGCCCGGGGCTCAACCCCGGGACTGCATTTGGAACTGTCAGGCTAGAGTGTCGGAGAGGAAAGCGGAATTCCTAGTGTAGCGGTGAAATGCGTAGATATTAGGAGGAACACCAGTGGCGAAGGCGGCTTTCTGGACGATGACTGACGTTGAGGCTCGAAAGCGTGGGGAGCAAACAGGATTAGATACCCTGGTAGTCCACGCCGTAAACGATGAATACTAGGTGTCGGGTGGCAAAGCCATTCGGTGCCGCAGCAAACGCAATAAGTATTCCACCTGGGGAGTACGTTCGCAAGAATGAAACTTAAATGAATTGACGGG

>OTU149

TAATACGTAGGgggCAAGCGTTATCCGGATTCATTGGGCGTAAAGCGCTCGTAGGCGGTCTGTTAGGTCGGGAGTTAAATCCGGgggCTCAACCcccGCTCGCTCCCGATACCGGCAGACTTGAGTTTGGTAGGGGAAGGTGGAATTCCTAGTGTAGCGGTGGAATGCGCAGATATTAGGAAGAACACCAGTGGCGAAGGCGGCCTTCTGGGCCATAACTGACGCTGAGGAGCGAAAGCTAGGGGAGCAAACAGGATTAGATACCCTGGTAGTCCTAGCCGTAAACGATGGACACTAGGTGTGGgggAATATTTCTTCCGTGCCGCAGCTAACGCATTAAGTGTCCCGCCTGGGGAGTACGGCCGCAAGGCTAAAACTCAAATGAATTGACGG

>OTU150

TAATACGTAGGTGGCAAGCGTTGTCCGGAATTATTGGGCGTAAAGCgcgcgcAGGCGGCCTATCCAGTCTGTCTTAAAAGTTCGGGGCTCAACCCCGTGATGGGATGGAAACTAGTAGGCTAGAGTATCGGAGAGGAAAGCGGAATTCCTAGTGTAGCGGTGAAATGCGTAGATATTAGGAAGAACACCAGTGGCGAAGGCGGCTTTCTGGACGAAAACTGACGCTGAGGCGCGAAAGCCAGGGGAGCGAACGGGATTAGATACCCCGGTAGTCCTGGCCGTAAACGATGGGTACTAGGTGTAGGAGGTATCGACCCCTTCTGTGCCGGAGTTAACGCAATAAGTACCCCGCCTGGGGAGTACGGTCGCAAGGCTGAAACTTAAATGAATTGACGG

>OTU151

TAATACGTAGGTGGCAAGCGTTGTCCGGATTTATTGGGCGTAAAGCGAGCGCAGGCGGAAGAATAAGTCTGATGTGAAAGCCCTCGGCTTAACCGAGGAACTGCATCGGAAACTGTTtttCTTGAGTGCAGAAGAGGAGAGTGGAACTCCATGTGTAGCGGTGGAATGCGTAGATatatGGAAGAACACCAGTGGCGAAGGCGGCTctctGGTCTGCAACTGACGCTGAGGCTCGAAAGCATGGGTAGCGAACAGGATTAGATACCCTGGTAGTCCATGCCGTAAACGATGAGTGCTAAGTGTTGGGAGGTTTCCGCCTCTCAGTGCTGCAGCTAACGCATTAAGCACTCCGCCTGGGGAGTACGACCGCAAGGTTGAAACTTAAAGGAATTGACGG

>OTU152

TAATACGGAAGGTCCGGGCGTTATCCGGATTTATTGGGTTTAAAGGGAGCGTAGGCTGGAGATTAAGTgtgtTGTGAAATGTAGACGCTCAACGTCTGACTTGCAGCGCATACTGGTTTCCTTGAGTACGCACAACGTTGGCGGAATTCGTCGTGTAGCGGTGAAATGCTTAGATATGACGAAGAACTCCGATTGCGAAGGCAGCTGACGGGAGCGCAACTGACGCTGAAGCTCGAAGGTGCGGGTATCGAACAGGATTAGATACCCTGGTAGTCCGCACAGTAAACGATGGATGCCCGCTGTTGGTACCTGGTATCAGCGGCTAAGCGAAAGCATTAAGCATCCCACCTGGGGAGTACGCCGGCAACGGTGAAACTTAAATGAATTGACGG

>OTU153

TAATACAGAGGATGCAAGCGTTATCCGGAATGATTGGGCGTAAAGCGTCTGTAGGTGGCTTtttAAGTCCGCCGTCAAATCCCAGGGCTCAACCCTGGACAGGCGGTGGAAACTACCAAGCTGGAGTACGGTAGGGGCAGAGGGAATTTCCGGTGGAGCGGTGAAATGCGTAGAGATCGGAAAGAACACCAACGGCGAAAGCACTCTGCTGGGCCGACACTGACACTGAgagaCGAAAGCTAGGGGAGCGAATGGGATTAGATACCCCAGTAGTCCTAGCCGTAAACGATGGATACTAGGCGCTGTGCGTATCGACCCGTGCAGTGCTGTAGCTAACGCGTTAAGTATCCCGCCTGGGGAGTACGTTCGCAAGAATGAAACTTAAATGAATTGACGG

>OTU154

TAATACGTAGGGTGCGAGCGTTAATCGGAATTACTGGGCGTAAAGCGGGCGCAGACGGTTACTTAAGCAGGATGTGAAATCCCCGGGCTCAACCTGGGAACTGCGTTCTGAACTGGGTGACTAGAGTgtgtCAGAGGGAGGTAGAATTCCACGTGTAGCAGTGAAATGCGTAGAGATGTGGAGGAATACCGATGGCGAAGGCAGCCTCCTGGGATAACACTGACGTTCATGCCCGAAAGCGTGGGTAGCAAACAGGATTAGATACCCTGGTAGTCCACGCCCTAAACGATGTCGATTAGCTGTTGGGCAGCATGACTGCTTAGTAGCGAAGCTAACGCGTGAAATCGACCGCCTGGGGAGTACGGTCGCAAGATTAAAACTTAAATGAATTGACGG

>OTU155

TAATACGTAGGTGGCAAGCGTTATCCGGATTTATTGGGCGTAAAGCGAGCGCAGGCGGTTttttAAGTCTGATGTGAAAGCCCTCGGCTTAACCGAGGAAGCGCATCGGAAACTGGGAAACTTGAGTGCAGAAGAGGACAGTGGAACTCCATGTGTAGCGGTGAAATGCGTAGATatatGGAAGAACACCAGTGGCGAAGGCGGCTGTCTGGTCTGTAACTGACGCTGAGGCTCGAAAGCATGGGTAGCGAACAGGATTAGATACCCTGGTAGTCCATGCCGTAAACGATGAATGCTAGGTGTTGGAGGGTTTCCGCCCTTCAGTGCCGCAGCTAACGCATTAAGCATTCCGCCTGGGGAGTACGACCGCAAGGTTGAAACTTAAATGAATTGACGGG

>OTU156

TAATACAGAGGGTGCAAGCGTTAATCGGAATTACTGGGCGTAAAGCgcgcgTAGGTGGTTCGTTAAGTTGGATGTGAAATCCCCGGGCTCAACCTGGGAACTGCATCCAAAACTGGCGAGCTAGAGTAGGGCAGAGGGTGGTGGAATTTCCTGTGTAGCGGTGAAATGCGTAGATATAGGAAGGAACACCAGTGGCGAAGGCGACCACCTGGGCTCATACTGACACTGAGGTGCGAAAGCGTGGGGAGCAAACAGGATTAGATACCCTGGTAGTCCACGCCGTAAACGATGTCAACTAGCCGTTGGAATCCTTGAGATTTTAGTGGCGCAGCTAACGCATTAAGTTGACCGCCTGGGGAGTACGGCCGCAAGGTTAAAACTTAAATGAATTGACGGG

>OTU157

TCATACGTAGGGTGCAAGCATTATCCGGAGTGACTGGGCGTAAAGAGTTGCGTAGGCGGTTTAATAAGTGAATAGTGAAACCTGGTGGCTCAACCATACAGACTATTATTCAAACTGTTAAACTCGAGAATGGTAGAGGTAACTGGAATTTCTAGTGTAGGAGTGAAATCCGTAGATATTAGAAGGAACACCGATGGCGTAGGCAGGTTACTGGGCCATTTCTGACGCTAAGGCACGAAAGCGTGGGGAGCGAACCGGATTAGATACCCGGGTAGTCCACGCCGTAAACGATGGATACTAGCTGTTGGAGGTATCGACCCCTTCAGTAGCGAAGCTAACGCGTTAAGTATCCCGCCTGTGGAGTACGGCCGCAAGGCTAAAACTTAAATGAATTGACGG

>OTU158

TAATACGTAGGTGGCGAGCGTTGTCCGGATTTATTGGGCGTAAAGGGAGTGTAGGCGGTCTTTTAAGTCTGATGTGAAAGCCCACGGCTCAACCGTGGAGGGTCATTGGAAACTGGGAGACTTGAGTGCAGAAGAGGAGAGCGGAATTCCATGTGTAGCGGTGAAATGCGTAGATatatGGAGGAACACCAGTGGCGAAGGCGGCTctctGGTCTGTAACTGACGCTGAGGCTCGAAAGCGTGGGGAGCAAACAGGATTAGATACCCTGGTAGTCCACGCCGTAAACGATGAGTGCTAAGTGTTGGAGGGGTTCCACCCTTCAGTGCTGGAGTTAACGCAATAAGCACTCCGCCTGGGGAGTACGGCCGCAAGGCTGAAACTTAAAGGAATTGACGG

>OTU159

TAATACGTATGTCACAAGCGTTATCCGGATTTATTGGGCGTAAAGCGCGTCTAGGTGGTTATGTAAGTCTGATGTGAAAATGCAGGGCTCAACTCTGTATTGCGTTGGAAACTGCATGACTAGAGTACTGGAGAGGTAAGCGGAACTACAAGTGTAGAGGTGAAATTCGTAGATATTTGTAGGAATGCCGATGGGGAAGCCAGCTTACTGGACAGATACTGACGCTAAAGCGCGAAAGCGTGGGTAGCAAACAGGATTAGATACCCTGGTAGTCCACGCCGTAAACGATGATTACTAGGTGTTGGgggTCGAACCTCAGCGCCCAAGCTAACGCGATAAGTAATCCGCCTGGGGAGTACGTACGCAAGTATGAAACTCAAATGAATTGACGG

>OTU160

TAATACGTAGGGTGCAAGCGTTGTCCGGATTTACTGGGCGTAAAGAGCTCGTAGGTGGTgtgtCGCGTCGTCTGTGAAATTCCGGGGCTTAACTCCGGGCGTGCAGGCGATACGGGCACGACTAGAGTGCTGTAGGGGTAACTGGAATTCCTGGTGTAGCGGTGAAATGCGCAGATATCAGGAGGAACACCGATGGCGAAGGCAGGTTACTGGGCAGTTACTGACGCTGAGGAGCGAAAGCATGGGGAGCGAACAGGATTAGATACCCTGGTAGTCCATGCTGTAAACGGTGGGCGCTAGGTGTGGGCTGtgtgtTTGCAGTCTGTGCCGTAGCTAACGCATTAAGCGCCCCGCCTGGGGAGTACGGCCGCAAGGCTAAAACTTAAAGGAATTGACGG

>OTU161

TAATACGTAGGgggCAAGCGTTATCCGGATTTACTGGGTGTAAAGGGAGCGTAGACGGACGGGCAAGTCTGATGTGAAAGCCCGGGGCTTAACCCCGGGACTGCATTGGAAACTGTCCATCTTGAGTGCCGGAGAGGTAAGCGGAATTCCTAGTGTAGCGGTGAAATGCGTAGATATTAGGAGGAACACCAGTGGCGAAGGCGGCTTACTGGACGGTAACTGACGTTGAGGCTCGAAAGCGTGGGGAGCAAACAGGATTAGATACCCTGGTAGTCCACGCCGTAAACGATGAATACTAGGTGTCGGGTTGCAAAGCAATTCGGTGCCGCAGCAAACGCAGTAAGTATTCCACCTGGGGAGTACGTTCGCAAGAATGAAACTTAAATGAATTGACGG

>OTU162

TAATACGAAGGgggCGAGCGTTGCTCGGAATGACTGGGCGTAAAGGGCGCGTAGGCGGCGGACACAGTCAGGCGTGAAATTCCTGGGCTCAACCTGGgggCTGCGCTTGATACGTGTTTGCTAGAGTTAGGAAGAGGGTCGTGGAATTCCCAGTGTAGAGGTGAAATTCGTAGATATTGGGAAGAACACCGGTGGCGAAGGCGGCGACCTGGTCCTGGACTGACGCTGAGGCGCGAAAGCGTGGGGAGCAAACAGGATTAGATACCCTGGTAGTCCACGCTGTAAACGATGtgtgCTGGATGTTGGGTAACCTAGTTACTCAGTGTCGTAGCTAACGCGATAAGCACACCGCCTGGGGAGTACGGCCGCAAGGTTGAAACTTAAATGAATTGACGG

>OTU163

TAATACGTAGGgggCAAGCGTTATCCGGATTTACTGGGTGTAAAGGGAGCGTAGACGGTAAAGCAAGTCTGAAGTGAAAGCCCGCGGCTCAACTGCGGGACTGCTTTGGAAACTGTTTAACTGGAGTGTCGGAGAGGTAAGTGGAATTCCTAGTGTAGCGGTGAAATGCGTAGATATTAGGAGGAACACCAGTGGCGAAGGCGACTTACTGGACGATAACTGACGTTGAGGCTCGAAAGCGTGGGGAGCAAACAGGATTAGATACCCTGGTAGTCCACGCCGTAAACGATGAATACTAGGTGTTGGGGAGCAAAGCTCTTCGGTGCCGTCGCAAACGCAGTAAGTATTCCACCTGGGGAGTACGTTCGCAAGAATGAAACTTAAAGGAATTGACGGC

>OTU164

TAATACGTAGGgggCAAGCGTTATCCGGATTTACTGGGTGTAAAGGGAGCGTAGACGGAGCAGCAAGTCTGATGTGAAAGGCGGgggCTCAACCcccGGACTGCATTGGAAACTGTTGATCTTGAGTACCGGAGAGGTAAGCGGAATTCCTAGTGTAGCGGTGAAATGCGTAGATATTAGGAGGAACACCAGTGGCGAAGGCGGCTTACTGGACGGTAACTGACGTTGAGGCTCGAAAGCGTGGGGAGCAAACAGGATTAGATACCCTGGTAGTCCACGCCGTAAACGATGAATACTAGGTGTCGGGTGGCAGAGCCATTCGGTGCCGCAGCAAACGCAGTAAGTATTCCACCTGGGGAGTACGTTCGCAAGAATGAAACTTAAAGGAATTGACGGG

>OTU165

TAATACGGAGGATGCGAGCGTTATCCGGAATCATTGGGTTTAAAGGGTCTGTAGGCGGGTTATTAAGTCAAGGGTGAAAGTTTGTTGCTTAACGATAAAATTGCCTTTGATACTGGTAGTCTTGAATTtttGTGAAGTTGCTGGAATGTGTAGTGTAGCGGTGAAATGCATAGATATTACACAGAACACCGATTGCGAAGGCATGTGACTAACAAAGGATTGACGCTGAgagaCGAAAGCGTGGGGAGCGAACAGGATTAGATACCCTGGTAGTCCACGCTGTAAACGATGGATACTAGCTGTTTGGAGCGATCTGAGTGGCTAAGCGAAAGTGATAAGTATCCCACCTGGGGAGTACGCACGCAAGTGTGAAACTCAAAGGAATTGACGG

>OTU166

TAATACGAAGGgggCTAGCGTTGCTCGGAATCACTGGGCGTAAAGGGCGCGTAGGCGGCGTTTTAAGTCGGgggTGAAAGCCTGTGGCTCAACCACAGAATGGCCTTCGATACTGGGACGCTTGAGTCTGGTAGAGGTTGGTGGAACTGCGAGTGTAGAGGTGAAATTCGTAGATATTCGCAAGAACACCGGTGGCGAAGGCGGCCAACTGGACCAGTACTGACGCTGAGGCGCGAAAGCGTGGGGAGCAAACAGGATTAGATACCCTGGTAGTCCACGCCGTAAACGATGAATGCTAGCTGTTGGGGTGCATGCACCGCAGTAGCGCAGCTAACGCATTAAGCATTCCGCCTGGGGAGTACGGTCGCAAGATTAAAACTTAAAGGAATTGACGG

>OTU167

TAATACGGAGGGTGCAAGCGTTAATCGGAATTACTGGGCGTAAAGCGCACGCAGGCGGTTTGTTAAGTTGGATGTGAAATCCCCGGGCTTAACCTGGGAACTGCATCCAAGACTGGCAAGCTAGAGTCTCGTAGAGGGAGGTAGAATTCCAGGTGTAGCGGTGAAATGCGTAGAGATCTGGAGGAATACCGGTGGCGAAGGCGGCCTCCTGGACGAAGACTGACGCTCAGGTGCGAAAGCGTGGGGAGCAAACAGGATTAGATACCCTGGTAGTCCACGCTGTAAACGATGTCGATTTGGAGGTTGTGCCCTTGAGGCGTGGCTTCCGAAGCTAACGCGTTAAATCGACCGCCTGGGGAGTACGGCCGCAAGGTTAAAACTTAAAGGAATTGACGG

>OTU168

TAATACAGAGGGTGCGAGCGTTAATCGGAATTACTGGGCGTAAAGCGAGTGTAGGTGGCTCATTAAGTCACATGTGAAATCCCCGGGCTTAACCTGGGAACTGCATGTGATACTGGTGGTGCTAGAATATGTGAGAGGGAAGTAGAATTCCAGGTGTAGCGGTGAAATGCGTAGAGATCTGGAGGAATACCGATGGCGAAGGCAGCTTCCTGGCATAATATTGACACTGAGATTCGAAAGCGTGGGTAGCAAACAGGATTAGATACCCTGGTAGTCCACGCCGTAAACGATGTCTACTAGCCGTTGGGGTCCTTGAGACTTTAGTGGCGCAGTTAACGCGATAAGTAGACCGCCTGGGGAGTACGGCCGCAAGGTTAAAACTCAAATGAATTGACGG

>OTU169

TAATACGTAGGTGGCAAGCGTTATCCGGATTTATTGGGCGTAAAGCGAGCGCAGGCGGTTGCTTAGGTCTGATGTGAAAGCCTTCGGCTTAACCGAAGAAGTGCATCGGAAACCGGGCGACTTGAGTGCAGAAGAGGACAGTGGAACTCCATGTGTAGCGGTGGAATGCGTAGATatatGGAAGAACACCAGTGGCGAAGGCGGCTGTCTGGTCTGCAACTGACGCTGAGGCTCGAAAGCATGGGTAGCGAACAGGATTAGATACCCTGGTAGTCCATGCCGTAAACGATGAGTGCTAGGTGTTGGAGGGTTTCCGCCCTTCAGTGCCGGAGCTAACGCATTAAGCACTCCGCCTGGGGAGTACGACCGCAAGGTTGAAACTCAAAGGAATTGACGG

>OTU170

TAATACGGAGGGTGCAAGCGTTGTCCGGATTTATTGGGTTTAAAGGGTGCGTAGGTGGGACTTCAAGTCTGGTTTGAAAGCTGGTCGCTCAACGATCAGATGTGGCTGGAAACTGAGGTTCTTGAATGGCGTAGCGGTAGCCGGAATGGGTCATGTAGCGGTGAAATGCATAGATATGACCCGGAACTCCGATTGCGAAGGCAGGCTACTGGGCGCCGATTGACACTGAGGCACGAGAGCATGGGTAGCAAACAGGATTAGATACCCTGGTAGTCCATGCCGTAAACGATGATTACTGGCTGTCTGCGCATGGCGTGGGTGGCTGAGCGAAAGCGTTAAGTAATCCACCTGGGGAGTACGCCGGCAACGGTGAAACTCAAATGAATTGACGG

>OTU171

TAATACGTAGGGTGCGAGCGTTGTCCGGAATTATTGGGCGTAAAGGGCTTGTAGGCGGTTTGTCGCGTCTGCCGTGAAATCCTCTGGCTTAACTGGgggCGTGCGGTGGGTACGGGCAGGCTTGAGTGCGGTAGGGGAGACTGGAACTCCTGGTGTAGCGGTGGAATGCGCAGATATCAGGAAGAACACCGGTGGCGAAGGCGGGTCTCTGGGCCGTTACTGACGCTGAGGAGCGAAAGCGTGGGGAGCGAACAGGATTAGATACCCTGGTAGTCCACGCTGTAAACGTTGGGCACTAGGTGTGGgggCCACCCGTGGTTTCTGCGCCGTAGCTAACGCTTTAAGTGCCCCGCCTGGGGAGTACGGCCGCAAGGCTAAAACTCAAATGAATTGACGG

>OTU172

TAATACGGAGGGTGCAAGCGTTAATCGGAATTACTGGGCGTAAAGCgcgcgTAGGTGGCTAAGTCAGCCAGGTGTGAAAGCCCCGGGCTCAACCTGGGAACGGCATCTGGAACTGCTTGGCTAGAGTGCAGGAGAGGAAGGTAGAATTCCCGGTGTAGCGGTGAAATGCGTAGAGATCGGGAGGAATACCAGTGGCGAAGGCGGCCTTCTGGACTGACACTGACACTGAGGTGCGAAAGCGTGGGTAGCAAACAGGATTAGATACCCTGGTAGTCCACGCCGTAAACGATGTCAACTAGCCGTTGGGTCCCTTGAGGACTTAGTGGCGCAGCTAACGCAATAAGTTGACCGCCTGGGGAGTACGGCCGCAAGGTTAAAACTTAAATGAATTGACGG

>OTU173

TAAAACGTAGGTCACAAGCGTTGTCCGGAATTACTGGGTGTAAAGGGAGCGCAGGCGGGAGAACAAGTTGGAAGTGAAATCCATGGGCTCAACCCATGAACTGCTTTCAAAACTGTTtttCTTGAGTAGTGCAGAGGTAGGCGGAATTCCCGGTGTAGCGGTGGAATGCGTAGATATCGGGAGGAACACCAGTGGCGAAGGCGGCCTACTGGGCACCAACTGACGCTGAGGCTCGAAAGTGTGGGTAGCAAACAGGATTAGATACCCTGGTAGTCCACACCGTAAACGATGATTACTAGGTGTTGGAGGATTGACCCCTTCAGTGCCGCAGTTAACACAATAAGTAATCCACCTGGGGAGTACGACCGCAAGGTTGAAACTTAAAGGAATTGACGG

>OTU174

TAATACGTAGGTGGCAAGCGTTGTCCGGATTTACTGGGCGTAAAGGGAGCGTAGGCGGATTtttAAGTGGGATGTGAAATACCCGGGCTCAACCTGGGTGCTGCATTCCAAACTGGAAATCTAGAGTGCAGGAGGGGAAAGTGGAATTCCTAGTGTAGCGGTGAAATGCGTAGAGATTAGGAAGAACACCAGTGGCGAAGGCGACTTTCTGGACTGTAACTGACGCTGAGGCTCGAAAGCGTGGGGAGCAAACAGGATTAGATACCCTGGTAGTCCACGCCGTAAACGATGAATACTAGGTGTAGGGGTTGTCATGACCTCTGTGCCGCCGCTAACGCATTAAGTATTCCGCCTGGGGAGTACGGTCGCAAGATTAAAACTCAAAGGAATTGACGGG

>OTU175

TAATACGTAGGTGGCAAGCGTTGTCCGGAATTATTGGGCGTAAAGGGCgcgcAGGCGGCGTCGTAAGTCGGTCTTAAAAGTGCGGGGCTTAACCCCGTGAGGGGACCGAAACTGCGATGCTAGAGTATCGGAGAGGAAAGCGGAATTCCTAGTGTAGCGGTGAAATGCGTAGATATTAGGAGGAACACCAGTGGCGAAAGCGGCTTTCTGGACGACAACTGACGCTGAGGCGCGAAAGCCAGGGGAGCAAACGGGATTAGATACCCCGGTAGTCCTGGCCGTAAACGATGGATACTAGGTGTAGGAGGTATCGACCCCTTCTGTGCCGGAGTTAACGCAATAAGTATCCCGCCTGGGGAGTACGGCCGCAAGGCTGAAACTTAAAGGAATTGACGG

>OTU176

TAATACGAAGGGTGCAAGCGTTAATCGGAATTACTGGGCGTAAAGCgcgcgTAGGTGGCTTGATAAGTTGGATGTGAAATCCCCGGGCTCAACCTGGGAACTGCATCCAAAACTGTCTGGCTAGAGTGCGGTAGAGGGTAGTGGAATTTCCAGTGTAGCGGTGAAATGCGTAGATATTGGAAGGAACACCAGTGGCGAAGGCGACTACCTGGACTGACACTGACACTGAGGTGCGAAAGCGTGGGGAGCAAACAGGATTAGATACCCTGGTAGTCCACGCCGTAAACGATGTCAACTAGCCGTTGGGATCCTTGAGATCTTAGTGGCGCAGCTAACGCATTAAGTTGACCGCCTGGGGAGTACGGCCGCAAGGTTAAAACTTAAAGGAATTGACGG

>OTU177

TAATACGTAGGgggCAAGCGTTATCCGGATTTACTGGGTGTAAAGGGAGCGTAGACGGCATGACAAGCCAGATGTGAAAACCCAGGGCTCAACCCTGGGACTGCATTTGGAACTGCCAGGCTGGAGTGCAGGAGAGGTAAGCGGAATTCCTAGTGTAGCGGTGAAATGCGTAGATATTAGGAGGAACACCAGTGGCGAAGGCGGCTTACTGGACTGTAACTGACGTTGAGGCTCGAAAGCGTGGGGAGCAAACAGGATTAGATACCCTGGTAGTCCACGCGGTAAACGATGATTGCTAGGTGTAGGTGGGTATGGACCCATCGGTGCCGCAGCTAACGCAATAAGCAATCCACCTGGGGAGTACGTTCGCAAGAATGAAACTTAAATGAATTGACGG

>OTU178

TAATACGTAGGGTGCAAGCGTTAATCGGAATTACTGGGCGTAAAGCGTGCGCAGGCGGTTCTGTAAGACAGATGTGAAATCCCCGGGCTCAACCTGGGAATGGCATTTGTGACTGCAGGACTAGAGTTCATCAGAGGggggTGGAATTCCAAGTGTAGCAGTGAAATGCGTAGATATTTGGAAGAACACCAATGGCGAAGGCAGCCcccTGGGATGCGACTGACGCTCATGCACGAAAGCGTGGGGAGCAAACAGGATTAGATACCCTGGTAGTCCACGCCCTAAACGATGTCTACTGGTTGTTGGGAATTTAATATTCTTGGTAACGAAGCTAACGCGTGAAGTAGACCGCCTGGGGAGTACGGTCGCAAGATTAAAACTTAAATGAATTGACGG

>OTU179

TAATACGTAGGTGGCAAGCGTTGTCCGGATTTATTGGGCGTAAAGCGAGTGCAGGCGGTTttttAAGTCTGATGTGAAAGCCTTCGGCTTAACCGAAGAAATGCATTGGAAACTGGGAAACTTGAGTGCAGAAGAGGAGAGTGGAACTCCATGTGTAGCGGTGAAATGCGTAGATatatGGAAGAACACCAGTGGCGAAGGCGGCTctctGGTCTGTAACTGACGCTGAGGCTCGAAAGCGTGGGTAGCAAACAGGATTAGATACCCTGGTAGTCCACGCCGTAAACGATGAGTGCTAAGTGTTGGAGGGTTTCCGCCCTACAGTGCTGCAGCTAACGCATTAAGCACTCCGCCTGGGGAGTACGACCGCAAGGTTGAAACTCAAAGGAATTGACGG

>OTU180

AATTGAAATAAaaaaCCCGATGCGCAGATCATCGGGTTCATTTCAATTGAGGAAATCGGGAGAATTACGCCACTTCTGACGCCGCGCTATCTAACAGCGCCATCTCTTCGCTGTTCAGCAGTTtttCGATGTTCACCAGAATCAACATACGGTCGCCCAGTGCGCCCAGTCCAGTGAGATATTCTGTTGAAAGCGTCACGGCAAACTCTGGTGCCGGACGAATTTGCTCCGCCGTCAATGAAAGCACGTCTGAAACGCCGTCAACCACGATGCCGACCACCCGCTGTCCGAGATTCAGGACGATAACTACCGTGTTGTCGTTATAGTCCACATCCACCTGGCTGAACTTAAATGAATTGACGG

>OTU181

TAAGACGGAGGATGCAAGTGTTATCCGGAATCACTGGGCGTAAAGCGTCTGTAGGTTGTTCAATAAGTCAACTGTTAAATCTTGAGGCTCAACTTCAAAATCGCAGTCGAAACTATTAAACTAGAGTATAGTAGGGGTAAAGGGAATTTCCAGTGGAGCGGTGAAATGCGTAGAGATTGGAAAGAACACCAATGGCGAAAGCACTTTACTGGGCTATTACTGACACTGAgagaCGAAAGCTAGGGTAGCAAATGGGATTAGATACCCCAGTAGTCCTAGCCGTAAACGATGGATACTAGATGTTGAACAGATCGACCTGTGCAGTATCAAAGCTAACGCGTTAAGTATCCCGCCTGGGAAGTATGCTCGCAAGAGTGAAACTTAAATGAATTGACGG

>OTU182

TAATACGTAGGgggCGAGCGTTATCCGGATTCATTGGGCGTAAAGCgcgcgTAGGCGGCCCGGCAGGCCGGgggTCGAAGCGGggggCTCAACCccccGAAGCCcccGGAACCTCCGCGGCTTGGGTCCGGTAGGGGAGGGTGGAACACCCGGTGTAGCGGTGGAATGCGCAGATATCGGGTGGAACACCGGTGGCGAAGGCGGCCCTCTGGGCCGAGACCGACGCTGAGGCGCGAAAGCTGGgggAGCGAACAGGATTAGATACCCTGGTAGTCCCAGCCGTAAACGATGGACGCTAGGTGTGGggggACGATCCccccGTGCCGCAGCCAACGCATTAAGCGTCCCGCCTGGGGAGTACGGCCGCAAGGCTAAAACTTAAATGAATTGACGGT

>OTU183

TAATACGGAATGTGCAAACGTTATTCGGAATCACTGGGCTTAAAGAGTCCGTAGGCGGCTTGTTAGGTGAGGTGTGAAATCCcccGGCTCAACCGTGGAATTGCGCTTCAAACCGGCAAGCTTGAGGAGGATAGAGGTGGGCGGAACTGATGGTGGAGCGGTGAAATGCGTTGATATCATCAGGAACACCGGAGGCGAAAGCGGTCCACTGGGTCCTTTCTGACGCTGAGGGACGAAAGCTAGGGGAGCGAACGGGATTAGATACCCCGGTAGTCCTAGCCTTAAACGATGAGCACTAGACCGAAGAATCTCCACGATTttttGGTCGTAGCGAAAGTGTTAAGTGCTCCGCCTGGGGAGTATGGTCGCAAGGCTGAAACTCAAATGAATTGACGG

>OTU184

TAAGACAGAGGAGGCAAGCGTTGTTCGGAATCATTGGGCTTAAAGGGCGTGCAGGCGGCGGCGCAAGTGTCGAGTGAAATCCCATCGCTCAACGATGGAACGGCTCGGCAAACTGCGTCGCTTGAGGTCGGTAGAGGTGCTCGGAACTCTAGGTGGAGCGGTGAAATGCGTAGATATCTAGAGGAACGCCTGAGGCGAAAGCGGGGCACTGGGCCGATTCTGACGCTGAGACGCGAAAGCGTGGGGAGCAAACAGGATTAGATACCCTGGTAGTCCACGCCGTAAACGATGCGCACTAGGTGACGGGGCCTCTCACGGCGCCGCCGCCGAAGCAAAAGTGATTAGTGCGCCGCCTGGGGAGTACGGCCGCAAGGCTAAAACTTAAATGAATTGACGG

>OTU185

TAATACGTATGGTGCAAGCGTTATCCGGATTTACTGGGTGTAAAGGGAGCGTAGACGGTTGTGTAAGTCTGATGTGAAAGCCCGGGGCTCAACCCCGGGACTGCATTGGAAACTATGTAACTAGAGTGTCGGAGAGGTAAGCGGAATTCCTAGTGTAGCGGTGAAATGCGTAGATATTAGGAGGAACACCAGTGGCGAAGGCGGCTTACTGGACGATCACTGACGTTGAGGCTCGAAAGCGTGGGGAGCAAACAGGATTAGATACCCTGGTAGTCCACGCCGTAAACGATGACTACTAGGTGTCGGGGAGCAAAGCTCTTCGGTGCCGCAGCAAACGCAATAAGTAGTCCACCTGGGGAGTACGTTCGCAAGAATGAAACTTAAATGAATTGACGG

>OTU186

TAATACGTAGGgggCAAGCGTTATCCGGAATTACTGGGTGTAAAGGGAGCGTAGGCGGCAGAGCAAGTCAGAAGTGAAAACCCAGGGCTCAACTTTGGGATTGCTTTTGAAACTGTTTTGCTAGAGTGTCGGAGAGGTAAGTGGAATTCCTAGTGTAGCGGTGAAATGCGTAGATATTAGGAGGAACACCAGTGGCGAAGGCGACTTACTGGACGATCACTGACGCTGAGGCTCGAAAGCGTGGGGAGCAAACAGGATTAGATACCCTGGTAGTCCACGCCGTAAACGATGAATACTAGGTGTTGGGGTCCATAAGGACTTCGGTGCCGCAGCTAACGCAATAAGTATTCCACCTGGGGAGTACGTTCGCAAGAATGAAACTCAAATGAATTGACGG

>OTU187

TAATACGTAGGgggCAAGCGTTATCCGGATTTACTGGGTGTAAAGGGAGCGTAGACGGTTAAGCAAGTCTGAAGTGAAAGCCCGGGGCTCAACCCCGGTACTGCTTTGGAAACTGTTTGACTTGAGTGCAGGAGAGGTAAGTGGAATTCCTAGTGTAGCGGTGAAATGCGTAGATATTAGGAGGAACACCAGTGGCGAAGGCGGCTTACTGGACTGTAACTGACGTTGAGGCTCGAAAGCGTGGGGAGCAAACAGGATTAGATACCCTGGTAGTCCACGCCGTAAACGATGAATACTAGGTGTCGGgggACAAAGTCCTTCGGTGCCGCCGCTAACGCAATAAGTATTCCACCTGGGGAGTACGTTCGCAAGAATGAAACTCAAAGGAATTGACGG

>OTU188

TAATACGTAGGTCCCGAGCGTTGTCCGGATTTATTGGGCGTAAAGCGAGCGCAGGCGGTTTAATAAGTCTGAAGTTAAAGGCAGTGGCTTAACCATTGTTTGCTTTGGAAACTGTTAAACTTGAGTGCAGAAGGGGAGAGTGGAATTCCATGTGTAGCGGTGAAATGCGTAGATatatGGAGGAACACCGGTGGCGAAAGCGGCTctctGGTCTGTAACTGACGCTGAGGCTCGAAAGCGTGGGGAGCAAACAGGATTAGATACCCTGGTAGTCCACGCCGTAAACGATGAGTGCTAGGTGTTAGGCCCTTTCCGGGGCTTAGTGCCGCAGCTAACGCATTAAGCACTCCGCCTGGGGAGTACGACCGCAAGGTTGAAACTCAAATGAATTGACGGG

>OTU189

TAATACGTAGGTGGCAAGCGTTGTCCGGATTTACTGGGCGTAAAGGGAGCGTAGGCGGATGTTTAAGTGGGATGTGAAATACTCGGGCTCAACTTGAGTGCTGCATTCCAAACTGGATATCTAGAGTGCAGGAGAGGAAAGGAGAATTCCTAGTGTAGCGGTGAAATGCGTAGAGATTAGGAAGAATACCAGTGGCGAAGGCGCCTTTCTGGACTGTAACTGACGCTGAGGCTCGAAAGCGTGGGGAGCAAACAGGATTAGATACCCTGGTAGTCCACGCCGTAAACGATGAATACTAGGTGTAGGGGTTGTCATGACCTCTGTGCCGCCGCTAACGCATTAAGTATTCCGCCTGGGGAGTACGGTCGCAAGATTAAAACTTAAATGAATTGACGGG

>OTU190

TAAAACGTAGGGTGCAAGCGTTGTCCGGAATTACTGGGTGTAAAGGGAGCGCAGGCGGATTGGCAAGTTGGGAGTGAAATCTATGGGCTCAACCCATAAATTGCTTTCAAAACTGTCAGTCTTGAGTGGTGTAGAGGTAGGCGGAATTCCCGGTGTAGCGGTGGAATGCGTAGATATCGGGAGGAACACCAGTGGCGAAGGCGGCCTACTGGGCACTAACTGACGCTGAGGCTCGAAAGCATGGGTAGCAAACAGGATTAGATACCCTGGTAGTCCATGCCGTAAACGATGATTACTAGGTGTGGGAGGATTGACCCCTTCCGTGCCGCAGTTAACACAATAAGTAATCCACCTGGGGAGTACGACCGCAAGGTTGAAACTCAAATGAATTGACGGC

>OTU191

TAATACGTATGTCGCAAGCGTTATCCGGAATTATTGGGCATAAAGGGCATCTAGGCGGCCAGACAAGTCTGGGGTGAAAACTTGCGGCTCAACCGCAAGCCTGCCCTGGAAACTGTTTGGCTAGAGTGCTGGAGAGGTGGACGGAACTGCACGAGTAGAGGTGAAATTCGTAGATATGTGCAGGAATGCCGATGATGAAGATAGTTCACTGGACGGTAACTGACGCTGAAGTGCGAAAGCTGGgggAGCGAACAGGATTAGATACCCTGGTAGTCCCAGCCGTAAACGATGATTACTGGGTGTGGGCATGAAGAGTGTCCGTGCCGAAGCTAATGCGATAAGTAATCCGCCTGGGGAGTACGGCCGCAAGGCTGAAACTCAAATGAATTGACGG

>OTU192

TAATACGTAGGTGGCGAGCGTTATCCGGATTTACTGGGCGTAAAGGATGCGTAGGTGGAATTTTAAGTGGGATGTGAAATACCCGGGCTCAACCTGGGAACTGCATTCCAAACTGGAATTCTAGAGTGCAGGAGAGGAAAGCGGAATTCCTAGTGTAGCGGTGAAATGCGTAGAGATTAGGAAGAACACCAGTGGCGAAGGCGGCTTTCTGGACTGTAACTGACACTGAGGCATGAAAGCGTGGGGAGCAAACAGGATTAGATACCCTGGTAGTCCACGCCGTAAACGATGGGTACTAGGTGTAGGGGTTTCGATACCTCTGTGCCGCCGTAAACACAATAAGTACCCCGCCTGGGGAGTACGGTCGCAAGATTAAAACTCAAATGAATTGACGG

>OTU193

TAATACGTATGGACCGAGCGTTGTCCGGAATCATTGGGCGTAAAGGGTACGTAGGCGGCCTAGTAAGTTAGAAGTGAAATAATATAGCTCAACTAtataAAGCTTTTAAAACTGTTAGGCTTGAgagaTGAAAGGGAAAGTGGAATTCCTAGTGTAGCGGTGAAATGCGCAGATATTAGGAAGAATACCGGTGGCGAAGGCGACTTTCTGGTCATCATCTGACGCTGAGGTACGAAAGCGTGGGTAGCAAACAGGATTAGATACCCTGGTAGTCCACGCTGTAAACGATGAGTGTTAGGTTCTTGGAATAATCTGGGAGCCGCAGCTAACGCATTAAACACTCCGCCTGGGGAGTACGCACGCAAGTGTGAAACTTAAATGAATTGACGG

>OTU194

TAATACGTAGGGAGCAAGCGTTGTCCGGAATTACTGGGCGTAAAGGGTGCGTAGGTGGCCATGTAAGTCAGGTGTGAAAGACCGGGGCTCAACCCCGGGGTTGCACTTGAAACTGtgtgGCTTGAGTACAGGAGAGGGAAGTGGAATTCCTAGTGTAGCGGTGAAATGCGTAGATATTAGGAGGAACACCAGTGGCGAAGGCGACTTTCTGGACTGTAACTGACACTGAGGCACGAAAGCGTGGGGAGCAAACAGGATTAGATACCCTGGTAGTCCACGCCGTAAACGATGGATACTAGGTGTGGggggCGATAGTCCTCCGTGCCGAAGCTAACGCATTAAGTATCCCGCCTGGGGAGTACGATCGCAAGGTTGAAACTTAAAGGAATTGACGG

>OTU195

TAATACGGAGGATCCGAGCGTTATCCGGATTTATTGGGTTTAAAGGGTGCGTAGGCGGCCCGGTAAGTTAGAGGTGAAATTCAGTGGCTCAACCACTGACCTGCCTCTGATACTGTCGGGCTTGAGATTAGATGCAGCAGGCGGAATGAGTAGTGTAGCGGTGAAATGCTTAGATATTACTCAGAACACCGATTGCGAAGGCAGCTTGCTAATCTATATCTGACGCTGAGGCACGAAAGCGTGGGTAGCGAACAGGATTAGATACCCTGGTAGTCCACGCAGTAAACGATGATAACTCGTTGTCGGCGATACACAGTCGGTGACCAAGCGAAAGCGATAAGTTATCCACCTGGGGAGTACGACCGCAAGGTTGAAACTCAAAGGAATTGACGG

>OTU196

TAATACGTAGGGCGCGAGCGTTGTCCGGAATTATTGGGCGTAAAGGGCTCGTAGGCGGCTTGTCGCGTCTGCTGTGAAAATGCGGGGCTTAACTCCGTACGTGCAGTGGGTACGGGCAGGCTAGAGTGCGGTAGGGGTGACTGGAATTCCTGGTGTAGCGGTGGAATGCGCAGATATCAGGAGGAACACCGATGGCGAAGGCAGGTCACTGGGCTGTTACTGACGCTGAGGAGCGAAAGCGTGGGGAGCGAACAGGATTAGATACCCTGGTAGTCCATGCTGTAAACGTTGGGAACTAGGTGTGGGGTTCTTTCCACGGATTCTGCGCCGGAGCTAACGCATTAAGTTCCCCGCCTGGGGAGTACGGCCGCAAGGCTAAAACTCAAAGGAATTGACGG

>OTU197

TAATACAGAGGGTGCAAGCGTTAATCGGAATTACTGGGCGTAAAGCgcgcgTAGGTGGTTTGTTAAGTTGGATGTGAAATCCCCGGGCTCAACCTGGGAACTGCATCCAAAACTGGCAAGCTAGAGTATGGTAGAGGGTGGTGGAATTTCCTGTGTAGCGGTGAAATGCGTAGATATAGGAAGGAACACCAGTGGCGAAGGCGACCACCTGGACTGATACTGACACTGAGGTGCGAAAGCGTGGGGAGCAAACAGGATTAGATACCCTGGTAGTCCACGCCGTAAACGATGTCAACTAGCCGTTGGGAGCCTTGAGCTCTTAGTGGCGCAGCTAACGCATTAAGTTGACCGCCTGGGGAGTACGGCCGCAAGGTTAAAACTCAAAGGAATTGACGG

>OTU198

TAATACGTAGGGAGCAAGCGTTGTCCGGATTTACTGGGTGTAAAGGGTGCGTAGGCGGCTTtttAAGTCGGGCGTGAAAGCTGTGGGCTTAACCCACAAATTGCGTTCGAAACTGGAAGGCTTGAGTGAAGTAGAGGTAGGCGGAATTCCCGGTGTAGCGGTGAAATGCGTAGAGATCGGGAGGAACACCAGTGGCGAAGGCGGCCTACTGGGCTTTAACTGACGCTGAGGCACGAAAGCATGGGTAGCAAACAGGATTAGATACCCTGGTAGTCCATGCCGTAAACGATGATTACTAGGTGTGGggggTCTGACCCCTTCCGTGCCGGAGTTAACACAATAAGTAATCCACCTGGGGAGTACGGCCGCAAGGTTGAAACTTAAATGAATTGACGG

>OTU199

TAATACGGAAGGTTCGGGCGTTATCCGGATTTATTGGGTTTAAAGGGAGCGTAGGCCGTTTGGTAAGCGTGTTGTGAAATGTAGGAGCTCAACTTCTAGATTGCAGCGCGAACTGTCAGACTTGAGTGCGCACAACGTAGGCGGAATTCATGGTGTAGCGGTGAAATGCTTAGATATCATGAAGAACTCCGATTGCGAAGGCAGCTTACGGGAGCGCAACTGACGCTGAAGCTCGAAGGTGCGGGTATCGAACAGGATTAGATACCCTGGTAGTCCGCACAGTAAACGATGGATGCCCGCTGTTAGCACCTAGTGTTAGCGGCTAAGCGAAAGCATTAAGCATCCCACCTGGGGAGTACGCCGGCAACGGTGAAACTCAAAGGAATTGACGG

>OTU200

TAATACGTAGGGAGCAAGCGTTGTCCGGATTTACTGGGTGTAAAGGGTGCGTAGGCGGCAGAGCAAGTCAGATGTGAAATCCGTGGGCTTAACCCACGAACTGCATTTGAAACTGTTTTGCTTGAGTGAAGTAGAGGCAGGCGGAATTCCCTGTGTAGCGGTGAAATGCGTAGAGATAGGGAGGAACACCAGTGGCGAAGGCGGCCTGCTGGGCTTTAACTGACGCTGAGGCACGAAAGCGTGGGTAGCAAACAGGATTAGATACCCTGGTAGTCCACGCTGTAAACGATGATTACTAGGTGTGGggggTCTGACCcccTCCGTGCCGCAGTTAACACAATAAGTAATCCACCTGGGGAGTACGGCCGCAAGGTTGAAACTTAAAGGAATTGACGG

>OTU201

TAATACGAAGGgggCTAGCGTTGTTCGGAATTACTGGGCGTAAAGCGCACGTAGGCGGATCGATCAGTCAGGGGTGAAATCCCAGGGCTTAACCCTGGAACTGCCTTTGATACTGTCGATCTAGAGTATGGAAGAGGTGAGTGGAATTCCGAGTGTAGAGGTGAAATTCGTAGATATTCGGAGGAACACCAGTGGCGAAGGCGGCTCACTGGTCCATTACTGACGCTGAGGTGCGAAAGCGTGGGGAGCAAACAGGATTAGATACCCTGGTAGTCCACGCCGTAAACGATGAATGTTAGCCGTCGGCAAGTTTACTTGTCGGTGGCGCAGCTAACGCATTAAACATTCCGCCTGGGGAGTACGGTCGCAAGATTAAAACTCAAATGAATTGACGG

>OTU202

TAATACGTAGGTGGCAAGCGTTGTCCGGAATTATTGGGCGTAAAGCgcgcgcAGGTGGTTTAATAAGTCTGATGTGAAAGCCCACGGCTCAACCGTGGAGGGTCATTGGAAACTGTTAAACTTGAGTGCAGGAgagaAAAGTGGAATTCCTAGTGTAGCGGTGAAATGCGTAGAGATTAGGAGGAACACCAGTGGCGAAGGCGGCTTtttGGCCTGTAACTGACACTGAGGCGCGAAAGCGTGGGGAGCAAACAGGATTAGATACCCTGGTAGTCCACGCCGTAAACGATGAGTGCTAAGTGTTGGTCTCATAAGAGATCAGTGCTGCAGCTAACGCATTAAGCACTCCGCCTGGGGAGTACGACCGCAAGGTTGAAACTTAAATGAATTGACGG

>OTU203

TAATACGTAGGgggCAAGCGTTATCCGGATTTACTGGGTGTAAAGGGAGCGTAGACGGTGTTGCAAGTCTGATGTGAAAGGCGGgggCTCAACCCCTGGATTGCATTGGAAACTGTGATACTCGAGTGCCGGAGAGGTAAGCGGAATTCCTAGTGTAGCGGTGAAATGCGTAGATATTAGGAGGAACACCAGTGGCGAAGGCGGCTTACTGGACGGTAACTGACGTTGAGGCTCGAAAGCGTGGGGAGCAAACAGGATTAGATACCCTGGTAGTCCACGCCGTAAACGATGAATACTAGGTGTCGGGGAGCAGAGCTCTTCGGTGCCGCAGCAAACGCAATAAGTATTCCACCTGGGGAGTACGTTCGCAAGAATGAAACTTAAAGGAATTGACGG

>OTU204

TAATACGGAGGGTGCAAGCGTTACCCGGAATCACTGGGCGTAAAGGGCGTGTAGGCGGGATGATCAGTCTGGTTTTAAAGACTGCGGCTCAACCGCAGGGATGGACTGGATACTGTCATTCTTGACCTCTGGAgagagaACTGGAATTCCTGGTGTAGCGGTGGAATGCGTAGATACCAGGAGGAACACCAATGGCGAAGGCAGGTTCTTGGACAGAAGGTGACGCTGAGGCGCGAAAGTGTGGGGAGCGAACCGGATTAGATACCCGGGTAGTCCACACCCTAAACGATGTACGTTGGCTAATCGCAGGATGCTGTGATTGGCGAAGCTAACGCGATAAACGTACCGCCTGGGAAGTACGGCCGCAAGGTTGAAACTTAAAGGAATTGACGG

>OTU205

TAATACGTAGGgggCAAGCGTTATCCGGATTTACTGGGTGTAAAGGGAGCGTAGACGGAGCGGCAAGTCTGGAGTGAAAACCCGGGGCTTAACCCTGGGACTGCTTTGGAAACTGTCGTTCTTGAGTGCCGGAGGGGTAAGCGGAATTCCTAGTGTAGCGGTGAAATGCGTAGATATTAGGAGGAACACCAGTGGCGAAGGCGGCTTACTGGACGGCAACTGACGTTGAGGCTCGAAAGCGTGGGGAGCAAACAGGATTAGATACCCTGGTAGTCCACGCCGTAAACGATGAATACTAGGTGTCGGTAAGCAAAGCTTATCGGTGCCGCAGCAAACGCAATAAGTATTCCACCTGGGGAGTACGTTCGCAAGAATGAAACTTAAAGGAATTGACGG

>OTU206

TAATACGTAGGTGGCAAGCGTTGTCCGGATTTACTGGGCGTAAAGGGAGCGTAGGTGGATATTTAAGTGGGATGTGAAATACCCGGGCTTAACCTGGGTGCTGCATTCCAAACTGGATATCTAGAGTGCAGGAGAGGAAAGGAGAATTCCTAGTGTAGCGGTGAAATGCGTAGAGATCTGGAGGAATACCGGTGGCGAAGGCGGCCcccTGGACAAAGACTGACGCTCAGGTGCGAAAGCGTGGGGAGCAAACAGGATTAGATACCCTGGTAGTCCACGCCGTAAACGATGTCGATTTGGAGGTTGTGCCCTTGAGGCGTGGCTTCCGGAGCTAACGCGTTAAATCGACCGCCTGGGGAGTACGGCCGCAAGGTTAAAACTTAAAGGAATTGACGG

>OTU207

TAATACGTAGGTGGCAAGCGTTGTCCGGAATTATTGGGCGTAAAGCgcgcgcAGGCGGATAGGTCAGTCTGTCTTAAAAGTTCGGGGCTTAACCCCGTGATGGGATGGAAACTGCCAATCTAGAGTATCGGAGAGGAAAGTGGAATTCCTAGTGTAGCGGTGAAATGCGTAGATATTAGGAAGAACACCAGTGGCGAAGGCGACTTTCTGGACGAAAACTGACGCTGAGGCGCGAAAGCCAGGGGAGCGAACGGGATTAGATACCCCGGTAGTCCTGGCCGTAAACGATGGGTACTAGGTGTAGGAGGTATCGACCCCTTCTGTGCCGGAGTTAACGCAATAAGTACCCCGCCTGGGGAGTACGACCGCAAGGTTGAAACTTAAATGAATTGACGGG

>OTU208

TAATACGTAGGGTGCAAGCGTTATCCGGAATTATTGGGCGTAAAGGGCTCGTAGGCGGTTCGTCGCGTCCGGTGTGAAAGTCCATCGCTTAACGGTGGATCCGCGCCGGGTACGGGCGGGCTTGAGTGCGGTAGGGGAGACTGGAATTCCCGGTGTAACGGTGGAATGTGTAGATATCGGGAAGAACACCAATGGCGAAGGCAGGTCTCTGGGCCGTTACTGACGCTGAGGAGCGAAAGCGTGGGGAGCGAACAGGATTAGATACCCTGGTAGTCCACGCCGTAAACGGTGGATGCTGGATGTGGGGCCCGTTCCACGGGTTCCGTGTCGGAGCTAACGCGTTAAGCATCCCGCCTGGGGAGTACGGCCGCAAGGCTAAAACTTAAATGAATTGACGGG

>OTU209

TAATACGTAGGGTGCGAGCGTTGTCCGGAATTACTGGGCGTAAAGGGCTCGTAGGTGGTTTGTCGCGTCGTCTGTGAAATTCCGGGGCTTAACTCCGGGCGTGCAGGCGATACGGGCATAACTTGAGTACTGTAGGGGTAACTGGAATTCCTGGTGTAGCGGTGAAATGCGCAGATATCAGGAGGAACACCGATGGCGAAGGCAGGTTACTGGGCAGTTACTGACGCTGAGGAGCGAAAGCATGGGTAGCGAACAGGATTAGATACCCTGGTAGTCCATGCCGTAAACGGTGGGCGCTAGGTGTGAGGGTCTTTTCACGACTTTCGTGCCGTAGCTAACGCATTAAGCGCCCCGCCTGGGGAGTACGGCCGCAAGGCTAAAACTTAAATGAATTGACGG

>OTU210

TAATACGGAAGGTCCGGGCGTTATCCGGATTTATTGGGTTTAAAGGGAGCGTAGGCCGCCCCTTAAGCGTGTTGTGAAATGCGGGTGCTCAACATCCGACTTGCAGCGCGAACTGGggggCTTGAGTGCGCCGAAAGTAGGCGGAATTCGTGGTGTAGCGGTGAAATGCTTAGATATCACGAAGAACTCCGATTGCGAAGGCAGCTTACTGTAGCGCAACTGACGCTGATGCTCGAAAGCGTGGGTATCGAACAGGATTAGATACCCTGGTAGTCCACGCGGTAAACGATGGATGCTCGTTGTCGGCCTTTGTGGTCGGTGACCAAGCGAAAGCGTTAAGCATCCCACCTGGGGAGTACGCCGGCAACGGTGAAACTTAAATGAATTGACGG

>OTU211

TAATACGAAGGgggCTAGCGTTGCTCGGAATTACTGGGCGTAAAGGGAGCGTAGGCGGACATTTAAGTCAGGGGTGAAATCCCGGGGCTCAACCTCGGAATTGCCTTTGATACTGGGTGTCTTGAGTATGAgagagGTgtgtgGAACTCCGAGTGTAGAGGTGAAATTCGTAGATATTCGGAAGAACACCAGTGGCGAAGGCGACacacTGGCTCATTACTGACGCTGAGGCTCGAAAGCGTGGGGAGCAAACAGGATTAGATACCCTGGTAGTCCACGCCGTAAACGATGATTGCTAGTTGTCGGGATGCATGCATTTCGGTGACGCAGCTAACGCATTAAGCAATCCGCCTGGGGAGTACGGTCGCAAGATTAAAACTTAAATGAATTGACGG

>OTU212

TAATACGTAGGgggCAAGCGTTATCCGGATTCATTGGGCGTAAAGCgcgcgTAGGCGGTCTGTTAGGTCAGGAGTTAAATCTGGgggCTCAACCCCTATCCGCTCCTGATACCGGCAGGCTTGAGTCTGGTAGGGGAAGATGGAATTCCAAGTGTAGCGGTGAAATGCGCAGATATTTGGAAGAACACCGGTGGCGAAGGCGGTCTTCTGGGCCATGACTGACGCTGAGGCGCGAAAGCTAGGGGAGCGAACAGGATTAGATACCCTGGTAGTCCTAGCTGTAAACGATGGACACTAGGTGTGGGGAGATTATACTTTCCGTGCCGCAGCTAACGCATTAAGTGTCCCGCCTGGGGAGTACGGTCGCAAGACTAAAACTTAAATGAATTGACGG

>OTU213

TAATACGTAGGTGGCAAGCGTTGTCCGGATTTATTGGGCGTAAAGCGAGTGCAGGCGGTTCAATAAGTCTGATGTGAAAGCCTTCGGCTCAACCGGAGAATTGCATCAGAAACTGTTGAACTTGAGTGCAGAAGAGGAGAGTGGAACTCCATGTGTAGCGGTGGAATGCGTAGATatatGGAAGAACACCAGTGGCGAAGGCGGCTctctGGTCTGCAACTGACGCTGAGGCTCGAAAGCATGGGTAGCGAACAGGATTAGATACCCTGGTAGTCCATGCCGTAAACGATGAGTGCTAAGTGTTGGGAGGTTTCCGCCTCTCAGTGCTGCAGCTAACGCATTAAGCACTCCGCCTGGGGAGTACGACCGCAAGGTTGAAACTTAAAGGAATTGACGG

>OTU214

TAATACGTAGGTGGCAAGCGTTGTCCGGAATTATTGGGCGTAAAGAGCGCGTAGGCGGTATGATAAGTCTGTCTTAGAAGTGCGGGGCTTAACCCCGTGAGGGGACAGAGACTGTCATACTAGAGTCTCGGAGAGGAAAGCGGAATTCCTAGTGTAGCGGTGAAATGCGTAGAGATTAGGAAGAACACCGGTGGCGAAGGCGGCTTTCTGGACGAGCACTGACGCTGAGGCGCGAAAGCGTGGGGAGCGAACAGGATTAGATACCCTGGTAGTCCACGCCGTAAACGATGGGTACTAGGTGTAGGAGGTATCGACCCCTTCTGTGCCGGAGTTAACGCAATAAGTACCCCGCCTGGGGAGTACGGTCGCAAGATTAAAACTTAAATGAATTGACGG

>OTU215

TAATACGTAGGgggCGAGCGTTGTTCGGAATTATTGGGCGTAAAGGGTACGTAGGCGGTTTGTTAAGTTTGGCGTTAAATCACGGGGCTCAACCCCGTTCAGCGTTGAAAACTGGCAAACTTGAGTAGTAGAGGGGACAGTGGAATTCCTAGTGTAGCGGTGAAATGCGTAGAGATTAGGAAGAATACCGGTGGCGAAGGCGACTGTCTGGATACATACTGACGCTCAGGTACGAAAGCGTGGGGAGCAAACAGGATTAGATACCCTGGTAGTCCACGCCCTAAACGATGAGTGCTAGGTGTCGGGTGTCAAAGCCCGGTGCCGCCGTTAACACATTAAGCACTCCGCCTGGGGAGTACGCACGCAAGTGTGAAACTCAAATGAATTGACGG

>OTU216

TAATACGGAAGGTCCGGGCGTTATCCGGATTTATTGGGTTTAAAGGGAGCGCAGGCCGTGGGTTAAGCGTGTCGTGAAATTCCGTCGCTCAACGGCGGACGTGCGGCGCGAACTGGTCCACTTGAGTACGCGGGACGTTGGCGGAATTCGTGGTGTAGCGGTGAAATGCTTAGATATCACGAAGAACTCCGATTGCGAAGGCAGCTGACGGTAGCGCAACTGACGCTGAGGCTCGAAAGTGCGGGTATCGAACAGGATTAGATACCCTGGTAGTCCGCACGGTAAACGATGGATGCCCGCTATTCGCCCACCTGGGCGAGTGGCCAAGCGAAAGCGTTAAGCATCCCACCTGGGGAGTACGCCGGCAACGGTGAAACTCAAAGGAATTGACGG

>OTU217

TAATACGAAGGgggCTAGCGTTGCTCGGAATTACTGGGCGTAAAGGGAGCGTAGGCGGACTGTTAAGTTAGAGGTGAAAGCCCAGGGCTCAACCTTGGAATTGCCTTTGATACTGGCAGTCTTGAGTACGGAAGAGGTATGTGGAACTCCGAGTGTAGAGGTGAAATTCGTAGATATTCGGAAGAACACCAGTGGCGAAGGCGACATACTGGTCCGTTACTGACGCTGAGGCTCGAAAGCGTGGGGAGCAAACAGGATTAGATACCCTGGTAGTCCACGCCGTAAACGATGAGTGCTAGTTGTCGGCATGCATGCATGTCGGTGACGCAGCTAACGCATTAAGCACTCCGCCTGGGGAGTACGGTCGCAAGATTAAAACTCAAATGAATTGACGG

>OTU218

TAATACGTAGGgggCGAGCGTTATCCGGATTCATTGGGCGTAAAGCgcgcgTAGGCGGCCGCGCAGGCGGggggTCAAATCCCGGGGCTCAACCCCGGGCCGCCccccGAACCGcgcgGCTTGGGTCCGGCAGGGGAGGGTGGAACGCCCGGTGTAGCGGTGGAATGCGCAGATATCGGGCGGAACACCGGTGGCGAAGGCGGCCCTCTGGGCCGGCACCGACGCTGAGGCGCGAGAGCTAGGGGAGCGAACAGGATTAGATACCCTGGTAGTCCTAGCCGTAAACGATGGACGCTAGGTGTGGGGACGATGCAGTCTCCGTGCCGCAGCCAACGCATTAAGCGTCCCGCCTGGGGAGTACGGCCGCAAGGCTAAAACTTAAATGAATTGACGG

>OTU219

TAATACGGAGGGTGCAAGCGTTAATCGGAATTACTGGGCGTAAAGCGCACGCAGGCGGTTGATTGAGTCAGATGTGAAATCCCCGGGCTTAACCCGGGAATTGCATCTGATACTGGTCAGCTAGAGTCTTGTAGAGGggggTAGAATTCCATGTGTAGCGGTGAAATGCGTAGAGATGTGGAGGAATACCGGTGGCGAAGGCGGCCcccTGGACAAAGACTGACGCTCAGGTGCGAAAGCGTGGGGAGCAAACAGGATTAGATACCCTGGTAGTCCACGCTGTAAACGATGTCGACTTGGAGGTTGTGCCCTTGAGGCGTGGCTTCCGGAGCTAACGCGTTAAGTCGACCGCCTGGGGAGTACGGCCGCAAGGTTAAAACTTAAATGAATTGACGG

>OTU220

TAATACGGAGGATGCAAGCGTTATCCGGAATGATTGGGCGTAAAGGGTCTGTAGGTGGTTGTTCAAGTCTGCTGTTAAAGCGTCTGGCTTAACCAGATAAAGGCGGTGGAAACTGAACGACTAGAGTGCGGTAGGGGTAGAGGGAATTCCCAGTGTAGCGGTGAAATGCGTAGATCTTGGGAAGAACACCTGTGGCGAAAGCGCTCTACTGGACCGCGACTGACACTCATGGACGAAAGCTAGGGGAGCGAATGGGATTAGATACCCCAGTAGTCCTAGCCGTAAACGATGGATACTAGGCGTTGCCCGTATCGACCCGGGCAGTGTCGTAGCTAACGCGTTAAGTATCCCGCCTGGGGAGTACGCAGGCAACTGTGAAACTTAAAGGAATTGACGG

>OTU221

TAATACGGAAGGTCCGGGCGTTATCCGGATTTATTGGGTTTAAAGGGAGCGTAGGCTGTCTATTAAGCGTGTTGTGAAATTTACCGGCTCAACCGGTGGCTTGCAGCGCGAACTGGTCGACTTGAGTATGCAGGAAGTAGGCGGAATTCATGGTGTAGCGGTGAAATGCTTAGATATCATGACGAACTCCGATTGCGCAGGCAGCTTACTGTAGCATAACTGACGCTGATGCTCGAAAGTGCGGGTATCAAACAGGATTAGATACCCTGGTAGTCCGCACGGTAAACGATGGATGCTCGCTATTCGTCCTATTTGGATGAGTGGCCAAGTGAAAACATTAAGCATCCCACCTGGGGAGTACGCCGGCAACGGTGAAACTTAAATGAATTGACGG

>OTU222

TAATACGTAGGgggCAAGCGTTATCCGGATTTACTGGGTGTAAAGGGAGCGTAGACGGCATAGCAAGTCTGAAGTGAAAGCCCGGGGCTCAACCCCGGAACTGCTTTGGAAACTGCCAAGCTAGAGTGTCGGAGAGGTAAGTGGAATTCCCAGTGTAGCGGTGAAATGCGTAGATATTGGGAGGAACACCGGTGGCGAAGGCGGCTTACTGGACGATAACTGACGTTGAGGCTCGAAGGCGTGGGGAGCAAACAGGATTAGATACCCTGGTAGTCCACGCGGTAAACGATGAATACTAGGTGTCGGGAGGATTAACCTTTCGGTGCCGTCGCAAACGCAGTAAGTATTCCACCTGGGGAGTACGTTCGCAAGAATGAAACTCAAATGAATTGACGG

>OTU223

TAATACAGAGGGTGCAAGCGTTAATCGGAATTACTGGGCGTAAAGCgcgcgTAGGTGGTTATTTAAGTCAGATGTGAAAGCCCCGGGCTTAACCTGGGAACTGCATCTGATACTGGATAACTAGAGTAGGTGAGAGGGGAGTAGAATTCCAGGTGTAGCGGTGAAATGCGTAGAGATCTGGAGGAATACCGATGGCGAAGGCAGCTCCCTGGCATCATACTGACACTGAGGTGCGAAAGCGTGGGTAGCAAACAGGATTAGATACCCTGGTAGTCCACGCCGTAAACGATGTCTACCAGTCGTTGGGTCTTTTAAAGACTTAGTGACGCAGTTAACGCAATAAGTAGACCGCCTGGGGAGTACGGCCGCAAGGTTAAAACTCAAATGAATTGACGG

>OTU224

TAATACGGAGGGTGCAAGCGTTATCCGGATTTATTGGGTTTAAAGGGTCCGTAGGTGGGCTGATAAGTCAGCGGTGAAATCCTGCAGCTTAACTGTAGAACTGCCGTTGATACTGTTAGTCTTGAGTGTATTTGAAGTGGCTGGAATAAGTAGTGTAGCGGTGAAATGCATAGATATTACTTAGAACACCAATTGCGAAGGCAGGTCACTAAGATACAACTGACGCTGAGGGACGAAAGCGTGGGGAGCGAACAGGATTAGATACCCTGGTAGTCCACGCTGTAAACGATGCTAACTCGTTtttGGTTtttATGAATCAGAGACTAAGCGAAAGTGATAAGTTAGCCACCTGGGGAGTACGCTCGCAAGAGTGAAACTTAAATGAATTGACGG

>OTU225

TAATACGGAGGGTGCGAGCGTTGTCCGGATTTATTGGGTTTAAAGGGTGCGTAGGCGGTTTATTAAGTCAGTGGTGAAAGACGGTCGCTCAACGATTGCAGTGCCATTGATACTGGTAGACTTGAGTTCGTATGAGGTAGCTGGAATGGATAGTGTAGCGGTGAAATGCATAGATATTATCCAGAACTCCAATTGCGTAGGCAGGTTACTAATACGATACTGACGCTGATGCACGAAAGTGTGGGGATCAAACAGGATTAGATACCCTGGTAGTCCACACTGTAAACGATGATGACTAACTGTTTGCCCTTTGGGGTGAGTGGTACAGAGAAATCGTTAAGTCATCCACCTGGGGAGTACGCCGGCAACGGTGAAACTTAAATGAATTGACGG

>OTU226

TAATACGTAGGGCGCAAGCGTTGTCCGGAATTATTGGGCGTAAAGAGCTCGTAGGCGGCTGGTCGCGTCTGTCGTGAAAACTTCCGGCTCAACCGGgggCTTGCGGTGGGTACGGGCCGGCTAGAGTGCGGTAGGGGTAACTGGAACTCCTGGTGTAGCGGTGGAATGCGCAGATATCAGGAAGAACACCGATGGCGAAGGCAGGTTACTGGGCCGTTACTGACGCTGAGGAGCGAAAGCGTGGGGAGCGAACAGGATTAGATACCCTGGTAGTCCACGCTGTAAACGTTGGGCACTAGGTGTGAGGCTCCTTTCCGGgggTTTTGCGCCGTAGCTAACGCATTAAGTGCCCCGCCTGGGGAGTACGGCCGCAAGGCTAAAACTCAAAGGAATTGACGG

>OTU227

TAATACGTAGGGTGCAAGCGTTAATCGGAATTACTGGGCGTAAAGGGTGCGCAGGCGGCTGTGCAAGACAGATGTGAAATCCCCGGGCTTAACCTGGGAACTGCATTTGTGACTGCACGGCTAGAGTTTGTCAGAGGAGGGTGGAATTCCGCGTGTAGCAGTGAAATGCGTAGATATGCGGAAGAACACCAATGGCGAAGGCAGCCCTCTGGGACATGACTGACGCTCATGCACGAAAGCGTGGGGAGCAAACAGGATTAGATACCCTGGTAGTCCACGCCCTAAACGATGTCAGCTGATTGTTCGGGAAGCAATTCCTGGGTAACCAAGCCAACGCGTGAAGCTGACCGCCTGGGAAGTACGGTCGCAAGATTAAAACTCAAAGGAATTGACGG

>OTU228

TAATACGGAGGGTGCAAGCGTTGTCCGGATTTACTGGGCGTAAAGGGAGCGTAGGTGGATATTTAAGTGGGATGTGAAATACCCGGGCTTAACCTGGGTGCTGCATTCCAAACTGGATATCTAGAGTGCAGGAGAGGAAAGGAGAATTCCTAGTGTAGCGGTGAAATGCGTAGAGATTAGGAAGAATACCAGTGGCGAAGGCGCCTTTCTGGACTGTAACTGACACTGAGGCTCGAAAGCGTGGGGAGCAAACAGGATTAGATACCCTGGTAGTCCACGCCGTAAACGATGAATACTAGGTGTAGGGGTTGTCATGACCTCTGTGCCGCCGCTAACGCATTAAGTATTCCGCCTGGGGAGTACGGTCGCAAGATTAAAACTCAAAGGAATTGACGG

>OTU229

TAATACGTAGGgggCAAGCGTTATCCGGATTTACTGGGTGTAAAGGGAGCGTAGACGGGCCAGCAAGTCTGATGTGAAAACCCGGGGCCCAACCCCGGGAGTGCATTGGAAACTGCGGGCCTGGAGTGCCGGAGAGGTAAGCGGAATTCCTAGTGTAGCGGTGAAATGCGTAGATATTAGGAGGAACACCAGTGGCGAAGGCGGCTTACTGGACGGTAACTGACGTTGAGGCTCGAAAGCGTGGGGAGCAAACAGGATTAGATACCCTGGTAGTCCACGCCGTAAACGATGACTACTAGGTGTCGGGGAGCAAAGCTGCCCGGTGCCGCAGCCAACGCAATAAGTAGTCCACCTGGGGAGTACGTTCGCAAGAATGAAACTTAAATGAATTGACGG

>OTU230

TAATACGTAGGTGGCGAGCGTTATCCGGATTTACTGGGCGTAAAGGGAGCGTAGGCGGATGATTAAGTGGGATGTGAAATACCCGGGCTCAACTTGGGTGCTGCATTCCAAACTGGTTATCTAGAGTGCAGGAGAGGAGAGTGGAATTCCTAGTGTAGCGGTGAAATGCGTAGAGATTAGGAAGAACACCAGTGGCGAAGGCGACTctctGGACTGTAACTGACGCTGAGGCTCGAAAGCGTGGGGAGCAAACAGGATTAGATACCCTGGTAGTCCACGCCGTAAACGATGAATACTAGGTGTGGgggTTTCAACACCTCCGTGCCGCCGCTAACGCATTAAGTATTCCGCCTGGGGAGTACGGTCGCAAGATTAAAACTTAAATGAATTGACGGT

>OTU231

TAATACGTAGGgggCAAGCGTTATCCGGATTTACTGGGTGTAAAGGGAGCGTAGGCGGCCATGCAAGTCAGAAGTGAAAGCCCGGGGCTCAACCCCGGGACTGCTTTTGAAACTGTAAGGCTAGATTGCCGGAGAGGTAAGCGGAATTCCTAGTGTAGCGGTGAAATGCGTAGATATTAGGAGGAACACCAGTGGCGAAGGCGGCTTACTGGACGGTGAATGACGCTGAGGCTCGAAAGCGTGGGGAGCAAACAGGATTAGATACCCTGGTAGTCCACGCCGTAAACGATGAATACTAGGTGTCGGGGTCCAGAAGGACTTCGGTGCCGCAGCAAACGCAATAAGTATTCCACCTGGGGAGTACGTTCGCAAGAATGAAACTTAAATGAATTGACGG

>OTU232

TAATACGTAGGGTGCGAGCGTTATCCGGAATTATTGGGCGTAAAGAGCTCGTAGGCGGTTTGTCGCGTCTGTCGTGAAAGTCCGGGGCTTAACCCCGGATCTGCGGTGGGTACGGGCAGACTAGAGTGCAGTAGGGGAGACTGGAATTCCTGGTGTAGCGGTGGAATGCGCAGATATCAGGAGGAACACCGATGGCGAAGGCAGGTCTCTGGGCTGTAACTGACGCTGAGGAGCGAAAGCATGGGGAGCGAACAGGATTAGATACCCTGGTAGTCCATGCCGTAAACGTTGGGCACTAGGTGTGGGGACCATTCCACGGTTTCCGCGCCGCAGCTAACGCATTAAGTGCCCCGCCTGGGGAGTACGGCCGCAAGGCTAAAACTTAAATGAATTGACGG

>OTU233

TAATACGTAGGgggCTAGCGTTATCCGGATTTACTGGGCGTAAAGGGTGCGTAGGTGGTCTTTCAAGTCGGTGGTTAAAGGCTACGGCTCAACCGTAGTTAGCCTCCGAAACTGGAAGACTTGAGTGCAGGAGAGGAAAGTGGAATTCCCAGTGTAGCGGTGAAATGCGTAGATATTGGGAGGAACACCAGTAGCGAAGGCGGCTTTCTGGACTGCAACTGACACTGAGGCACGAAAGCGTGGGTAGCAAACAGGATTAGATACCCTGGTAGTCCACGCTGTAAACGATGAGTACTAGGTGTCGGgggTTACCccccTCGGTGCCGCAGCTAACGCATTAAGTACTCCGCCTGGGGAGTACGCACGCAAGTGTGAAACTCAAATGAATTGACGG

>OTU234

TAATACGTAGGgggCAAGCGTTATCCGGATTTACTGGGTGTAAAGGGAGCGTAGACGGCGATGCAAGTCTGAAGTGAAAGCCCGGGGCTCAACCCCGGGACTGCTTTGGAAACTGtgtgGCTGGAGTGCAGGAGAGGTAAGTGGAATTCCTAGTGTAGCGGTGAAATGCGTAGATATTAGGAGGAACACCAGTGGCGAAGGCGGCTTACTGGACTGTAACTGACGTTGAGGCTCGAAAGCGTGGGGAGCAAACAGGATTAGATACCCTGGTAGTCCACGCCGTAAACGATGAATGCTAGGTGTCGGggggCAAAGCCCCTCGGTGCCGCCGCTAACGCAATAAGCATTCCACCTGGGGAGTACGTTCGCAAGAATGAAACTTAAATGAATTGACGG

>OTU235

TGATACAGGGGTGGCAAGCGTTGTCCGGATTTACTGGGTGTAAAGGGTGCGCAGGCGGATCAATAAGTCGGgggTTAAATCCATGTGCTTAACACATGCACGGCTTCCGATACTGTTGATCTAGAGTCTCGAAGAGGAAGGTGGAATTTCCGGTGTAACGGTGGAATGTGTAGATATCGGAAAGAACACCAGTGGCGAAGGCAGCCTTCTGGTCGAGTACTGACGCTCATGCACGAAAGCGTGGGGAGCAAACAGGATTAGATACCCTGGTAGTCCACGCCCTAAACGATGTCAACTGGTTGTTGGGTCTTCACTGACTCAGTAACGAAGCTAACGCGTGAAGTTGACCGCCTGGGGAGTACGGCCGCAAGGTTGAAACTTAAAGGAATTGACGG

>OTU236

TAATACGTAGGgggCAAGCGTTATCCGGATTTACTGGGTGTAAAGGGAGCGTAGACGGAAGAGCAAGTCTGATGTGAAAGGCTGGGGCTTAACCCCAGGACTGCATTGGAAACTGTTtttCTAGAGTGCCGGAGAGGTAAGCGGAATTCCTAGTGTAGCGGTGAAATGCGTAGATATTAGGAGGAACACCAGTGGCGAAGGCGGCTTACTGGACGGTAACTGACGTTGAGGCTCGAAAGCGTGGGGAGCAAACAGGATTAGATACCCTGGTAGTCCACGCCGTAAACGATGAATACTAGGTGTCGGGGTGCAAAGCAGTTCGGTGCCGCAGCAAACGCAATAAGTATTCCACCTGGGGAGTACGTTCGCAAGAATGAAACTCAAATGAATTGACGG

>OTU237

TAATACGTAGGGTGCGAGCGTTAATCGGAATTACTGGGCGTAAAGCGTGCGCAGGCGGTTGTGCAAGACAGATGTGAAATCCCCGGGCTTAACCTGGGAACTGCATTTGTGACTGCACGGCTAGAGTGCGGCAGAGGGGAGTGGAATTCCGCGTGTAGCAGTGAAATGCGTAGATATGCGGAGGAACACCGATGGCGAAGGCAGCTCCCTGGGCCTGCACTGACGCTCATGCACGAAAGCGTGGGGAGCAAACAGGATTAGATACCCTGGTAGTCCACGCCCTAAACGATGTCAACTGGTTGTTGGTCCTTCACTGGATCAGTAACGTAGCTAACGCGTGAAGTTGACCGCCTGGGGAGTACGGCCGCAAGGTTGAAACTCAAAGGAATTGACGG

>OTU238

TAATACCAGCACCCCGAGTGGTCGGGACGATTATTGGGCCTAAAGCATCCGTAGCCGGTTCTACAAGTCTTCCGTTAAATCCACCTGCTTAACAGATGGGCTGCGGAGGATACTATGGAGCTAGGAGGCGGGAGAGGCAAGCGGTACTTAGTGGGTAGGGGTAAAATCCGTTGATCCATTGAAGACCACCAGTGGCGAAGGCGGCTTGCCAGAACGCACTCGACGGTGAGGGATGAAAGCTGGgggAGCAAACCGGATTAGATACCCGGGTAGTCCCAGCTGTAAACGATGCAGACTCGGTGATGAATAGGCTTCGTGCCTATTCAGTGCCGCAGGGAAGCCGTTAAGTCTGCCGCCTGGGGAGTACGGTCGCAAGACTGAAACTCAAAGGAATTGACGG

>OTU239

TAATACGTAGGTGGCAAGCGTTGTCCGGATTTACTGGGCGTAAAGGATGCGTAGGCGGATGTTTAAGTGAGATGTGAAATACCCGGGCTCAACTTGGGTGCTGCATTTCAAACTGGACATCTAGAGTGCGGGAGAGGAAAGCGGAATTCCTAGTGTAGCGGTGAAATGCGTAGAGATCTGGAGGAATACCGGTGGCGAAGGCGGCCcccTGGACGAAGACTGACGCTCAGGTGCGAAAGCGTGGGGAGCAAACAGGATTAGATACCCTGGTAGTCCACGCCGTAAACGATGAATACTAGGTGTAGGAGGTATCGACTCCTTCTGTGCCGCAGTTAACACAATAAGTATTCCGCCTGGGAAGTACGATCGCAAGATTAAAACTTAAATGAATTGACGG

>OTU240

TAATACGGAGGGTGCAAGCGTTAATCGGAATTACTGGGCGTAAAGCGCACGCAGGCGGTCAATTAAGTCAGATGTGAAAGCCCCGAGCTTAACTTGGGAATTGCATCTGAAACTGGTTGGCTAGAGTCTTGTAGAGGggggTAGAATTCCATGTGTAGCGGTGAAATGCGTAGAGATGTGGAGGAATACCGGTGGCGAAGGCGGCCcccTGGACAAAGACTGACGCTCAGGTGCGAAAGCGTGGGGAGCAAACAGGATTAGATACCCTGGTAGTCCACGCTGTAAACGATGTCGATTTAGAGGTTGTGGTCTTGAACCGTGGCTTCTGGAGCTAACGCGTTAAATCGACCGCCTGGGGAGTACGGCCGCAAGGTTAAAACTTAAATGAATTGACGG

>OTU241

TGATACGTAGGGTGCGAGCGTTGTCCGGATTTATTGGGCGTAAAGGGCTCGTAGGCGGTTGATCGCGTCGGAAGTGGAAACTTGATGCTTAACGTTGAGCGTGCTTTCGATACGGGTTGACTTGAGGAAGGTAGGGGAGAATGGAATTCCTGGTGGAGCGGTGGAATGCGCAGATATCAGGAGGAACACCAGTGGCGAAGGCGGTTCTCTGGACCTTTCCTGACGCTGAGGAGCGAAAGCGTGGGGAGCGAACAGGCTTAGATACCCTGGTAGTCCACGCTGTAAACGGTGGGTACTAGGTGTGGGGTCCATTCCACGGATTCTGTGCCGTAGCTAACGCATTAAGTACCCCGCCTGGGGAGTACGGCCGCAAGGCTAAAACTCAAATGAATTGACGG

>OTU242

TAATACGTAGGTGGCAAGCGTTGTCCGGAATTATTGGGCGTAAAGCgcgcgcAGGCGGCTTCCTAAGTCCATCTTAAAAGTGCGGGGCTTAACCCCGTGATGGGATGGAAACTGGGAAGCTGGAGTATCGGAGAGGAAAGTGGAATTCCTAGTGTAGCGGTGAAATGCGTAGAGATTAGGAAGAACACCGGTGGCGAAGGCGACTTTCTGGACGAAAACTGACGCTGAGGCGCGAAAGCGTGGGGAGCAAACAGGATTAGATACCCTGGTAGTCCACGCCGTAAACGATGGATACTAGGTGTAGGAGGTATCGACCCCTTCTGTGCCGGAGTTAACGCAATAAGTATCCCGCCTGGGAAGTACGATCGCAAGATTAAAACTTAAATGAATTGACGG

>OTU243

TAATACGGAGGATCCGAGCGTTATCCGGATTTATTGGGTTTAAAGGGAGCGTAGGTGGACTGGTAAGTCAGTTGTGAAAGTTTGCGGCTCAACCGTAAAATTGCAGTTGATACTGTCAGTCTTGAGTACAGTAGAGGTGGGCGGAATTCGTGGTGTAGCGGTGAAATGCTTAGATATCACGAAGAACTCCGATTGCGAAGGCAGCTCACTGGACTGCAACTGACACTGATGCTCGAAAGTGTGGGTATCAAACAGGATTAGATACCCTGGTAGTCCAcacaGTAAACGATGAATACTCGCTGTTTGCGATATACAGTAAGCGGCCAAGCGAAAGCATTAAGTATTCCACCTGGGGAGTACGCCGGCAACGGTGAAACTTAAATGAATTGACGGG

>OTU244

TAATACGTATGGTGCAAGCGTTATCCGGATTTACTGGGTGTAAAGGGAGCGTAGACGGATAGGCAAGTCTGGAGTGAAAGCCCGGGGCTCAACCCCGGGACTGCTTTGGAAACTGTTTATCTAGAGTGCTGGAGAGGTAAGTGGAATTCCTAGTGTAGCGGTGAAATGCGTAGATATTAGGAGGAACACCAGTGGCGAAGGCGGCTTACTGGACAGTAACTGACGTTGAGGCTCGAAAGCGTGGGGAGCAAACAGGATTAGATACCCTGGTAGTCCACGCCGTAAACGATGACTGCTAGGTGTCGGGGAGCAAAGCTCTTCGGTGCCGCAGCAAACGCAATAAGCAGTCCACCTGGGGAGTACGTTCGCAAGAATGAAACTTAAATGAATTGACGG

>OTU245

TAATACGTAGGGGACAAGCGTTGTCCGGAATGACTGGGCGTAAAGGGCGCGTAGGCGGTCTATTAAGTCTGATGTGAAAGGTACCGGCTCAACCGGTGAAGTGCATTGGAAACTGGTAGACTTGAGTATTGGAGAGGCAAGTGGAATTCCTAGTGTAGCGGTGAAATGCGTAGATATTAGGAGGAACACCAGTGGCGAAGGCGGCTTGCTGGACAAATACTGACGCTGAGGTGCGAAAGCGTGGGGAGCGAACAGGATTAGATACCCTGGTAGTCCACGCCGTAAACGATGAATGCTAGGTGTTGGGGAAACTCAGTGCCGCAGTTAACACAATAAGCATTCCGCCTGGGGAGTACGACCGCAAGGTTGAAACTCAAATGAATTGACGG

>OTU246

TAATACGTAGGgggCGAGCGTTGTCCGGAGTTACTGGGCGTAAAGGGCCTGCAGGCGGTGCGGCGCGTTTCGCGTGACAGCGCAGGGCTTCACTCTGCGAGGGTGCGAAAGACGGCAGCACTTGAGGGCCAGagagGGACACGGAATTCCGGGTGGAGTGGTGAAATGCGTAGAGATCCGGAGGAACACCGAAGGCGAAGGCAGTGTCCTGGCTGGTGCCTGACGCTGAGAGGCGAAAGCCAGGGGAGCGAACGGGATTAGATACCCCGGTAGTCCTGGCCGTAAACGATGACCACCAGGTGTGCGGAGTATCGACCCTCTGCGTGCCGGAGTTAACacacTAAGTGGTCCGCCTGGGGAGTACGGTCGCAAGATTAAAACTCAAATGAATTGACGG

>OTU247

TAATACGTAGGGCGCAAGCGTTATCCGGATTTATTGGGCGTAAAGGGCTCGTAGGCGGCTCGTCGCGTCCGGTGTGAAAGTCCATCGCTTAACGGTGGATCTGCGCCGGGTACGGGCGGGCTGGAGTGCGGTAGGGGAGACTGGAATTCCCGGTGTAACGGTGGAATGTGTAGATATCGGGAAGAACACCGATGGCGAAGGCAGGTCTCTGGGCCGTCACTGACGCTGAGGAGCGAAAGCGTGGGGAGCGAACAGGATTAGATACCCTGGTAGTCCACGCCGTAAACGGTGGACGCTGGATGTGGGGCACGTTCCACGTGTTCCGTGTCGGAGCTAACGCGTTAAGCGTCCCGCCTGGGGAGTACGGCCGCAAGGCTAAAACTCAAATGAATTGACGG

>OTU248

TAATACGTAGGGTGCAAGCGTTAATCGGAATTACTGGGCGTAAAGCGTGCGCAGGCGGTTATGCAAGACAGATGTGAAATCCCCGGGCTCAACCTGGGAACTGCATTTGTGACTGCATGGCTAGAGTGCGGCAGAGGgggATGGAATTCCGCGTGTAGCAGTGAAATGCGTAGATATGCGGAGGAACACCGATGGCGAAGGCAATCCCCTGGGCCTGCACTGACGCTCATGCACGAAAGCGTGGGGAGCAAACAGGATTAGATACCCTGGTAGTCCACGCCCTAAACGATGTCAACTGGTTGTTGGGAGGGTTTCTTCTCAGTAACGAAGCTAACGCGTGAAGTTGACCGCCTGGGGAGTACGGCCGCAAGGTTGAAACTTAAAGGAATTGACGG

>OTU249

TAATACGTAGGGAGCGAGCGTTATCCGGATTCATTGGGCGTAAAGAGCGCGTAGGCGGCCTCTCAAGCGGGATCTCTAATCCGAGGGCTCAACCcccGGCCGGATCCCGAACTGGGAGGCTCGAGTTCGGTAGAGGCAGGCGGAATTCCCGGTGTAGCGGTGGAATGCGCAGATATCGGGAAGAACACCGATGGCGAAGGCAGCCTGCTGGGCCGCAACTGACGCTGAGGCGCGAAAGCTAGGGGAGCGAACAGGATTAGATACCCTGGTAGTCCTAGCCGTAAACGATGGATACTAGGTGTGGggggCTCCGCCCTCCGTGCCGCAGCCAACGCATTAAGTATCCCGCCTGGGGAGTACGGCCGCAAGGCTAAAACTTAAATGAATTGACGG

>OTU250

TAATACGTAGGGTCCGAGCGTTGTCCGGAGTTACTGGGCGTAAAGCgcgcgcAGGCGGCGGTGCTGGCCCGGCGTGAAAGCCcccGGCTCAACCGGGGAGGGTCGTCGGGGACCGCACCGCTTGAGGGCGGTAGGGGCTGGTGGAATGCCTGGTGTAGTGGTGAAATGCGTAGAGATCAGGCGGAACACCCGTGGCGAAGGCGGCCAGCTGGGCCGTCCCTGACGCTGAGGCGCGAAGGCGTGGGGAGCGAACGGGATTAGATACCCCGGTAGTCCACGCAGTAAACGATGCCGACTAGGCGTGGggggAGTTGACCcccTCCGTGCCGGAGCCAACGCGGGAAGTCGGCCGCCTGGGGAGTACGGCCGCAAGGCTAAAACTTAAAGGAATTGACGG

>OTU251

TAATACGTAGGTGGCAAGCGTTGTCCGGATTTATTGGGCGTAAAGCGAGCGCAGGCGGATTGATAAGTCTGATGTGAAAGCCTTCGGCTCAACCGAAGAACTGCATCAGAAACTGTCAATCTTGAGTGCAGAAGAGGAGAGTGGAACTCCATGTGTAGCGGTGGAATGCGTAGATatatGGAAGAACACCAGTGGCGAAGGCGGCTctctGGTCTGTAACTGACGCTGAGGCTCGAAAGCATGGGTAGCGAACAGGATTAGATACCCTGGTAGTCCATGCCGTAAACGATGAGTGCTAAGTGTTGGGAGGTTTCCGCCTCTCAGTGCTGCAGCTAACGCATTAAGCACTCCGCCTGGGGAGTACGACCGCAAGGTTGAAACTCAAATGAATTGACGG

>OTU252

TAATACGTAGGGTCCAAGCGTTAATCGGAATTACTGGGCGTAAAGCGTGCGCAGGCGGTTGTGCAAGACCGATGTGAAATCCCCGAGCTTAACTTGGGAATTGCATTGGTGACTGCACGGCTAGAGTgtgtCAGAGGggggTAGAATTCCACGTGTAGCAGTGAAATGCGTAGAGATGTGGAGGAATACCGATGGCGAAGGCAGCCcccTGGGATAACACTGACGCTCATGCACGAAAGCGTGGGGAGCAAACAGGATTAGATACCCTGGTAGTCCACGCCCTAAACGATGTCAACTAGTTGTTGGGGATTCATTTCCTTAGTAACGTAGCTAACGCGTGAAGTTGACCGCCTGGGGAGTACGGTCGCAAGATTAAAACTCAAATGAATTGACGG

>OTU253

TAATACGGAGGGTGCAAGCGTTAATCGGAATTACTGGGCGTAAAGCGCACGTAGGTGGTTTGTTAAGCCAGCTGTGAAATCCCCGGGCTCAACCTGGGCACTGCAGTTGGAACTGGCAAGCTAGAGTAGGGTAGAGGGGTGTGGAATTCCAGGTGTAGCGGTGAAATGCGTAGATATCTGGAGGAACATCAGTGGCGAAGGCGACACCCTGGACTCATACTGACACTGAGGTGCGAAAGCGTGGGGAGCAAACAGGATTAGATACCCTGGTAGTCCACGCCGTAAACGATGTCTACTAGCCGTTGGGAGCCTTGAGCTCTTAGTGGCGCAGCTAACGCACTAAGTAGACCGCCTGGGGAGTACGGTCGCAAGATTAAAACTTAAATGAATTGACGG

>OTU254

TAATACGTAGGgggCAAGCGTTATCCGGATTTACTGGGTGTAAAGGGAGCGTAGACGGTGTGGCAAGTCTGATGTGAAAGGCATGGGCTCAACCTGTGGACTGCATTGGAAACTGTCATACTTGAGTGCCGGAGGGGTAAGCGGAATTCCTAGTGTAGCGGTGAAATGCGTAGATATTAGGAGGAACACCAGTGGCGAAGGCGGCTTACTGGACGGTAACTGACGTTGAGGCTCGAAAGCGTGGGGAGCAAACAGGATTAGATACCCTGGTAGTCCACGCCGTAAACGATGAATACTAGGTGTCGGggggCATGGCTCTTCGGTGCCGTCGCAAACGCAGTAAGTATTCCACCTGGGGAGTACGTTCGCAAGAATGAAACTCAAATGAATTGACGG

>OTU255

TAATACGTAGGgggCTAGCGTTATCCGGAATTACTGGGCGTAAAGGGTGCGTAGGCGGTCTTTCAAGCCAGAAGTGAAAGGCTACGGCTCAACCGTAGTAAGCTTTTGGAACTGTAGGACTTGAGTGCAGGAGAGGAGAGTGGAATTCCTAGTGTAGCGGTGAAATGCGTAGATATTAGGAGGAACACCAGTAGCGAAGGCGGCTctctGGACTGTAACTGACGCTGAGGCACGAAAGCGTGGGGAGCAAACAGGATTAGATACCCTGGTAGTCCACGCCGTAAACGATGAGTACTAGGTGTCGGgggTTACCccccTCGGTGCCGCAGCTAACGCATTAAGTACTCCGCCTGGGAAGTACGCTCGCAAGAGTGAAACTCAAAGGAATTGACGG

>OTU256

TAATACGAAGGGTGCAAGCGTTAATCGGAATTACTGGGCGTAAAGCgcgcgTAGGTGGTTCAGTAAGTTGGAAGTGAAATCCCCGGGCTCAACCTGGGAACTGCTTTCAAAACTGCTGAGCTAGAGTACGGTAGAGGGTGGTGGAATTTCCTGTGTAGCGGTGAAATGCGTAGATATAGGAAGGAACACCAGTGGCGAAGGCGACCACCTGGACTGATACTGACACTGAGGTGCGAAAGCGTGGGGAGCAAACAGGATTAGATACCCTGGTAGTCCACGCCGTAAACGATGTCAACTAGCCGTTGGGAGTCTTGAACTCTTAGTGGCGCAGCTAACGCATTAAGTTGACCGCCTGGGGAGTACGGCCGCAAGGTTAAAACTTAAAGGAATTGACGG

>OTU257

TAATACGAAGGGTGCAAGCGTTAATCGGAATTACTGGGCGTAAAGGGTGCGTAGGCGGTTGTTTAAGTCTGCCGTGAAATCCCCGGGCTCAACCTGGGAATGGCGGTGGATACTGGGCAGCTAGAGTgtgtCAGAGGATGGTGGAATTCCCGGTGTAGCGGTGAAATGCGTAGAGATCGGGAGGAACATCAGTGGCGAAGGCGGCCATCTGGGACAACACTGACGCTGAAGCACGAAAGCGTGGGGAGCAAACAGGATTAGATACCCTGGTAGTCCACGCCCTAAACGATGCGAACTGGATGTTGGTCTCAACTCGGAGATCAGTGTCGAAGCAAACGCGTTAAGTTCGCCGCCTGGGGAGTACGGTCGCAAGACTGAAACTTAAATGAATTGACGG

>OTU258

TAATACGTAGGgggCAAGCGTTATCCGGATTTACTGGGTGTAAAGGGAGCGCAGACGGCAATGCAAGTCTGAAGTGAAAGGCGTGGGCTCAACCCATGAACTGCTTTGGAAACTGTATAGCTTGAGTGTCGGAGGGGTAAGCGGAATTCCTAGTGTAGCGGTGAAATGCGTAGATATTAGGAGGAACACCGGAGGCGAAGGCGGCTTACTGGACGACAACTGACGTTGAGGCTCGAAGGCGTGGGGAGCAAACAGGATTAGATACCCTGGTAGTCCACGCAGTAAACGATGAATACTTGGTGTCGGGGAGGTAAACTCTTCGGTGCCGCAAGCTAACGCATTAAGTATTCCACCTGGGGAGTACGTTCGCAAGAATGAAACTTAAAGGAATTGACGG

>OTU259

TAATACGTAGGgggCAAGCGTTATCCGGATTTACTGGGTGTAAAGGGAGCGTAGACGGCGCGGCAAGTCTGAAGTGAAAGCCCGGGGCTTAACCCCGGGACTGCTTTGGAAACTGCCATGCTGGAGTGCTGGAGAGGTAAGTGGAATTCCTAGTGTAGCGGTGAAATGCGTAGATATTAGGAGGAACACCAGTGGCGAAGGCGGCTTACTGGACAGTAACTGACGTTGAGGCTCGAAGGCGTGGGGAGCAAACAGGATTAGATACCCTGGTAGTCCACGCGGTAAACGATGAATACTAGGTGTCGGGGAGCAAAGCTTTTCGGTGCCGGCGCAAACGCAATAAGTATTCCACCTGGGGAGTACGTTCGCAAGAATGAAACTCAAATGAATTGACGG

>OTU260

TAATACGTAAGGACCGAGCGTTGTCCGGAATCATTGGGCGTAAAGGGTACGTAGGCGGCTAGAAAAGTTAGAAGTCAAAGGCTATAGCTCAACTATAGTAAGCTTCTAAAACTATTTAGCTTGAgagaTGGAAGGGAAAGTGGAATTCCTAGTGTAGCGGTGGAATGCGCAGATATTAGGAGGAATACCGGTGGCGAAGGCGACTTTCTGGCCATTTTCTGACGCTGAGGTACGAAAGCGTGGGTAGCAAACAGGATTAGATACCCTGGTAGTCCACGCCGTAAACGATGAGTGTTAGGTGTCTGGAGTCAAATCTGGATGCCGCAGCAAACGCATTAAACACTCCGCCTGGGGAGTACGCACGCAAGTGTGAAACTTAAATGAATTGACGG

>OTU261

TAATACGTAGGGCGCAAGCGTTGTCCGGAATTATTGGGCGTAAAGAGCTCGTAGGCGGTTTGTCGCGTCTGCTGTGAAAGCCCGGGGCTTAACCCCGGGTGTGCAGTGGGTACGGGCAGACTAGAGTGCAGTAGGGGAGACTGGAATTCCTGGTGTAGCGGTGGAATGCGCAGATATCAGGAGGAACACCGATGGCGAAGGCAGGTCTCTGGGCTGTTACTGACGCTGAGGAGCGAAAGCATGGGGAGCGAACAGGATTAGATACCCTGGTAGTCCATGCCGTAAACGTTGGGCACTAGGTGTGGgggACATTCCACGTTTTCCGCGCCGTAGCTAACGCATTAAGTGCCCCGCCTGGGGAGTACGGCCGCAAGGCTAAAACTTAAATGAATTGACGG

>OTU262

TAATACGGAGGATGCGAGCGTTATCCGGATTTATTGGGTTTAAAGGGAGCGCAGACGGGTCGTTAAGTCAGCTGTGAAAGTTTGGGGCTCAACCTTAAAATTGCAGTTGATACTGGCGTCCTTGAGTGCGGTTGAGGTGTGCGGAATTCGTGGTGTAGCGGTGAAATGCTTAGATATCACGAAGAACTCCGATTGCGAAGGCAGCACACTAATCCGTAACTGACGTTCATGCTCGAAAGTGTGGGTATCAAACAGGATTAGATACCCTGGTAGTCCACACGGTAAACGATGGATACTCGCTGTTGGCGATATACTGTCAGCGGCTTAGCGAAAGCGTTAAGTATCCCACCTGGGGAGTACGCCGGCAACGGTGAAACTTAAATGAATTGACGG

>OTU263

TAATACGTAGGTGGCGAGCGTTGTCCGGATTTACTGGGCGTAAAGGGAGCGTAGGCGGACTTTTAAGTGAGATGTGAAATACCCGGGCTCAACTTGGGTGCTGCATTTCAAACTGGAAGTCTAGAGTGCAGGAGAGGAGAATGGAATTCCTAGTGTAGCGGTGAAATGCGTAGAGATTAGGAAGAACACCAGTGGCGAAGGCGATTCTCTGGACTGTAACTGACGCTGAGGCTCGAAAGCGTGGGGAGCAAACAGGATTAGATACCCTGGTAGTCCACGCCGTAAACGATGAATACTAGGTGTAGGGGTTGTCATGACCTCTGTGCCGCCGCTAACGCATTAAGTATTCCGCCTGGGGAGTACGGTCGCAAGATTAAAACTCAAATGAATTGACGG

>OTU264

TAATACGGGAGTGGCAAGCGTTATCCGGAATTATTGGGCGTAAAGCGTCCGCAGGCGGCCTTGTAAGTCTGTCGTTAAAGCGTGGAGCTTAACTCCATTTAAGCGATGGAAACTACAAGGCTTGAGTGTGGTAGGGGCAGAGGGAATTCCCGGTGTAGCGGTGAAATGCGTAGATATCGGGAAGAACACCAGTGGCGAAGGCGCTCTGCTGGGCCATAACTGACGCTCATGGACGAAAGCCAGGGGAGCGAAAGGGATTAGATACCCCTGTAGTCCTGGCCGTAAACGATGAACACTAGGTGTCGGgggAATCGACCcccTCGGTGTCGTAGCCAACGCGTTAAGTGTTCCGCCTGGGGAGTACGCACGCAAGTGTGAAACTTAAAGGAATTGACGG

>OTU265

TAATACGTAGGGCGCAAGCGTTATCCGGAATTATTGGGCGTAAAGAGCTCGTAGGCGGTTTGTCGCGTCTGCCGTGAAAGTCCGGGGCTTAACTCCGGATCTGCGGTGGGTACGGGCAGACTTGAGTGATGTAGGGGAGACTGGAATTCCTGGTGTAGCGGTGAAATGCGCAGATATCAGGAGGAACACCGATGGCGAAGGCAGGTCTCTGGGCATTAACTGACGCTGAGGAGCGAAAGCATGGGGAGCGAACAGGATTAGATACCCTGGTAGTCCATGCCGTAAACGTTGGGCACTAGGTGTGGgggACATTCCACGTTTTCCGCGCCGTAGCTAACGCATTAAGTGCCCCGCCTGGGGAGTACGGCCGCAAGGCTAAAACTTAAATGAATTGACGG

>OTU266

TAATACGGAGGGTGCGAGCGTTAATCGGAATAACTGGGCGTAAAGGGCACGCAGGCGGCTATTTAAGTGAGGTGTGAAATCCCCGGGCTTAACCTGGGAATTGCATTTCAGACTGGGTAGCTAGAGTACTTTAGGGAGGGGTAGAATTCCACGTGTAGCGGTGAAATGCGTAGAGATGTGGAGGAATACCGAAGGCGAAGGCAGCCCCTTGGGAATGTACTGACGCTCATGTGCGAAAGCGTGGGGAGCAAACAGGATTAGATACCCTGGTAGTCCACGCTGTAAACGCTGTCGATTTGGGGATTGGGCTTTAAGCTTGGTGCCCGTAGCTAACGTGATAAATCGACCGCCTGGGGAGTACGGCCGCAAGGTTAAAACTTAAATGAATTGACGG

>OTU267

TAATACGTAGGTGGCAAGCGTTGTCCGGATTTACTGGGCGTAAAGGATGCGTAGGCGGATGTTTAAGTGAGATGTGAAATACCCGGGCTCAACTTGGGTGCTGCATTTCAAACTGGACATCTAGAGTGCGGGAGAGGAAAGCGGAATTCCTAGTGTAGCGGTGAAATGCGTAGAGATTAGGAAGAACACCAGTGGCGAAGGCGGCTTTCTGGACCGTAACTGACGCTGAGGCATGAAAGCGTGGGGAGCAAACAGGATTAGATACCCTGGTAGTCCACGCCGTAAACGATGAATACTAGGTGTAGGAGGTATCGACTCCTTCTGTGCCGCAGTTAACACAATAAGTATTCCGCCTGGGAAGTACGATCGCAAGATTAAAACTTAAATGAATTGACGGG

>OTU268

TAATACGTAGGGTGCGAGCGTTAATCGGAATTACTGGGCGTAAAGCGTGCGCAGGCGGTTTTGTAAGACAGGCGTGAAATCCCCGGGCTTAACCTGGGAATTGCGCTTGTGACTGCAAGGCTAGAGTGCGTCAGAGGggggTAGAATTCCACGTGTAGCAGTGAAATGCGTAGAGATGTGGAGGAATACCGATGGCGAAGGCAGCCcccTGGGACGTGACTGACGCTCATGCACGAAAGCGTGGGGAGCAAACAGGATTAGATACCCTGGTAGTCCACGCCCTAAACGATGTCAACTAGTTGTTGGGGATTCATTTTCTCAGTAACGTAGCTAACGCGTGAAGTTGACCGCCTGGGGAGTACGGTCGCAAGATTAAAACTCAAATGAATTGACGG

>OTU269

TAATACGTAGGGTGCGAGCGTTGTCCGGAATTACTGGGCGTAAAGAGCTCGTAGGTGGTTTGTCGCGTCGTTTGTGGAATACCGCAGCTTAACTGCGGGGTTGCAGGCGATACGGGCATAACTTGAGTGCTGTAGGGGAGACTGGAATTCCTGGTGTAGCGGTGGAATGCGCAGATATCAGGAGGAACACCGATGGCGAAGGCAGGTCTCTGGGCAGTAACTGACGCTGAGGAGCGAAAGCATGGGGAGCGAACAGGATTAGATACCCTGGTAGTCCATGCCGTAAACGGTGGGCGCTAGGTGTGAGTCCCTTCCACGGGGTTCGTGCCGTAGCTAACGCATTAAGCGCCCCGCCTGGGGAGTACGGCCGCAAGGCTAAAACTTAAATGAATTGACGG
