# Supplementary material for: Dysfunctional gut microbiota and relative co-abundance network in infantile eczema
Source: Gut Pathog. 2016 Jul 22;8:36. doi: 10.1186/s13099-016-0118-0 (PMC4957860; doi:10.1186/s13099-016-0118-0)
Supplement: Supplementary file 3 — 10.1186/s13099-016-0118-0 Basic Local Alignment Search Tool (BLAST) results of selected OTUs. The sequence of OTU1 and OTU2 were put into BLAST on the NCBI website. In (A), the sequence of OTU2 had 1 mismatch with Klebsiella quasipneumoniae and Enterobacter cloacae. In (B), OTU1 had 1 mismatch with Shigella sonnei and 3 mismatches with Cronobacter turicensis. Due to the high similarity between their V4-V5 regions, genus Klebsiella and Enterobacter could not be distinguished, as was similar for Shigella and Cronobacter. [file 13099_2016_118_MOESM3_ESM.pdf]

A

Klebsiella quasipneumoniae subsp. similipneumoniae strain 07A044 16S ribosomal RNA, partial sequence

Sequence ID: ref|NR_134063.1| **Length:** 1462 **Number of Matches:** 1

Related

Range 1: 502 to 897 GenBank Graphics ▼ Next Match ▲ Previous Match

Score	Expect	Identities	Gaps	Strand
726 bits(393)	0.0	395/396(99%)	0/396(0%)	Plus/Plus

```
Query  1    TAATACGGAGGGTGCAAGCGTTAATCGGAATTACTGGGCGTAAAGCGCACGCAGGCGGTC  60
Sbjct  502  TAATACGGAGGGTGCAAGCGTTAATCGGAATTACTGGGCGTAAAGCGCACGCAGGCGGTC  561
Query  61   TGTCAAGTCGGATGTGAAATCCCCGGGCTCAACCTGGGAACTGCATTCGAAACTGGCAGG  120
Sbjct  562  TGTCAAGTCGGATGTGAAATCCCCGGGCTCAACCTGGGAACTGCATTCGAAACTGGCAGG  621
Query  121  CTAGAGTCTTGTAGAGGGGGGTAGAATTCCAGGTGTAGCGGTGAAATGCGTAGAGATCTG  180
Sbjct  622  CTAGAGTCTTGTAGAGGGGGGTAGAATTCCAGGTGTAGCGGTGAAATGCGTAGAGATCTG  681
Query  181  GAGGAATACCGGTGGCGAAGGCGGCCCCCTGGACAAAGACTGACGCTCAGGTGCGAAAGC  240
Sbjct  682  GAGGAATACCGGTGGCGAAGGCGGCCCCCTGGACAAAGACTGACGCTCAGGTGCGAAAGC  741
Query  241  GTGGGGAGCAAACAGGATTAGATACCCTGGTAGTCCACGCCGTAAACGATGTCGATTTGG  300
Sbjct  742  GTGGGGAGCAAACAGGATTAGATACCCTGGTAGTCCACGCCGTAAACGATGTCGATTTGG  801
Query  301  AGGTTGTGCCCTTGAGGCGTGGCTTCCGGAGCTAACGCGTTAAATCGACCGCCTGGGGAG  360
Sbjct  802  AGGTTGTGCCCTTGAGGCGTGGCTTCCGGAGCTAACGCGTTAAATCGACCGCCTGGGGAG  861
Query  361  TACGGCCGCAAGGTTAAAACTTAAATGAATTGACGG  396
Sbjct  862  TACGGCCGCAAGGTTAAAACTCAAATGAATTGACGG  897
```

Enterobacter cloacae subsp. dissolvens strain ATCC 23373 16S ribosomal RNA gene, partial sequence

Sequence ID: ref|NR_118011.1| **Length:** 1507 **Number of Matches:** 1

Related

Range 1: 520 to 915 GenBank Graphics ▼ Next Match ▲ Previous Match

Score	Expect	Identities	Gaps	Strand
726 bits(393)	0.0	395/396(99%)	0/396(0%)	Plus/Plus

```
Query  1    TAATACGGAGGGTGCAAGCGTTAATCGGAATTACTGGGCGTAAAGCGCACGCAGGCGGTC  60
Sbjct  520  TAATACGGAGGGTGCAAGCGTTAATCGGAATTACTGGGCGTAAAGCGCACGCAGGCGGTC  579
Query  61   TGTCAAGTCGGATGTGAAATCCCCGGGCTCAACCTGGGAACTGCATTCGAAACTGGCAGG  120
Sbjct  580  TGTCAAGTCGGATGTGAAATCCCCGGGCTCAACCTGGGAACTGCATTCGAAACTGGCAGG  639
Query  121  CTAGAGTCTTGTAGAGGGGGGTAGAATTCCAGGTGTAGCGGTGAAATGCGTAGAGATCTG  180
Sbjct  640  CTAGAGTCTTGTAGAGGGGGGTAGAATTCCAGGTGTAGCGGTGAAATGCGTAGAGATCTG  699
Query  181  GAGGAATACCGGTGGCGAAGGCGGCCCCCTGGACAAAGACTGACGCTCAGGTGCGAAAGC  240
Sbjct  700  GAGGAATACCGGTGGCGAAGGCGGCCCCCTGGACAAAGACTGACGCTCAGGTGCGAAAGC  759
Query  241  GTGGGGAGCAAACAGGATTAGATACCCTGGTAGTCCACGCCGTAAACGATGTCGATTTGG  300
Sbjct  760  GTGGGGAGCAAACAGGATTAGATACCCTGGTAGTCCACGCCGTAAACGATGTCGATTTGG  819
Query  301  AGGTTGTGCCCTTGAGGCGTGGCTTCCGGAGCTAACGCGTTAAATCGACCGCCTGGGGAG  360
Sbjct  820  AGGTTGTGCCCTTGAGGCGTGGCTTCCGGAGCTAACGCGTTAAATCGACCGCCTGGGGAG  879
Query  361  TACGGCCGCAAGGTTAAAACTTAAATGAATTGACGG  396
Sbjct  880  TACGGCCGCAAGGTTAAAACTCAAATGAATTGACGG  915
```

B

Shigella sonnei Ss046 strain Ss046 16S ribosomal RNA, complete sequence

Sequence ID: ref|NR_074894.1| **Length:** 1542 **Number of Matches:** 1

Range 1: 531 to 926 GenBank Graphics ▼ Next Match ▲ Previous Match

Score	Expect	Identities	Gaps	Strand
726 bits(393)	0.0	395/396(99%)	0/396(0%)	Plus/Plus

```
Query  1    TAATACGGAGGGTGCAAGCGTTAATCGGAATTACTGGGCGTAAAGCGCACGCAGGCGGTT  60
Sbjct  531  TAATACGGAGGGTGCAAGCGTTAATCGGAATTACTGGGCGTAAAGCGCACGCAGGCGGTT  590
Query  61   TGTTAAGTCAGATGTGAAATCCCCGGGCTCAACCTGGGAACTGCATCTGATACTGGCAAG  120
Sbjct  591  TGTTAAGTCAGATGTGAAATCCCCGGGCTCAACCTGGGAACTGCATCTGATACTGGCAAG  650
Query  121  CTTGAGTCTCGTAGAGGGGGGTAGAATTCCAGGTGTAGCGGTGAAATGCGTAGAGATCTG  180
Sbjct  651  CTTGAGTCTCGTAGAGGGGGGTAGAATTCCAGGTGTAGCGGTGAAATGCGTAGAGATCTG  710
Query  181  GAGGAATACCGGTGGCGAAGGCGGCCCCCTGGACGAAGACTGACGCTCAGGTGCGAAAGC  240
Sbjct  711  GAGGAATACCGGTGGCGAAGGCGGCCCCCTGGACGAAGACTGACGCTCAGGTGCGAAAGC  770
Query  241  GTGGGGAGCAAACAGGATTAGATACCCTGGTAGTCCACGCCGTAAACGATGTCGACTTGG  300
Sbjct  771  GTGGGGAGCAAACAGGATTAGATACCCTGGTAGTCCACGCCGTAAACGATGTCGACTTGG  830
Query  301  AGGTTGTGCCCTTGAGGCGTGGCTTCCGGAGCTAACGCGTTAAGTCGACCGCCTGGGGAG  360
Sbjct  831  AGGTTGTGCCCTTGAGGCGTGGCTTCCGGAGCTAACGCGTTAAGTCGACCGCCTGGGGAG  890
Query  361  TACGGCCGCAAGGTTAAAACTTAAATGAATTGACGG  396
Sbjct  891  TACGGCCGCAAGGTTAAAACTCAAATGAATTGACGG  926
```

Cronobacter turicensis strain z3032 16S ribosomal RNA gene, complete sequence

Sequence ID: ref|NR_102802.1| **Length:** 1543 **Number of Matches:** 1

Range 1: 531 to 926 GenBank Graphics ▼ Next Match ▲ Previous Match

Score	Expect	Identities	Gaps	Strand
715 bits(387)	0.0	393/396(99%)	0/396(0%)	Plus/Plus

```
Query  1    TAATACGGAGGGTGCAAGCGTTAATCGGAATTACTGGGCGTAAAGCGCACGCAGGCGGTT  60
Sbjct  531  TAATACGGAGGGTGCAAGCGTTAATCGGAATTACTGGGCGTAAAGCGCACGCAGGCGGTT  590
Query  61   TGTTAAGTCAGATGTGAAATCCCCGGGCTCAACCTGGGAACTGCATCTGATACTGGCAAG  120
Sbjct  591  TGTTAAGTCAGATGTGAAATCCCCGGGCTCAACCTGGGAACTGCATTTGAAACTGGCAAG  650
Query  121  CTTGAGTCTCGTAGAGGGGGGTAGAATTCCAGGTGTAGCGGTGAAATGCGTAGAGATCTG  180
Sbjct  651  CTTGAGTCTCGTAGAGGGGGGTAGAATTCCAGGTGTAGCGGTGAAATGCGTAGAGATCTG  710
Query  181  GAGGAATACCGGTGGCGAAGGCGGCCCCCTGGACGAAGACTGACGCTCAGGTGCGAAAGC  240
Sbjct  711  GAGGAATACCGGTGGCGAAGGCGGCCCCCTGGACGAAGACTGACGCTCAGGTGCGAAAGC  770
Query  241  GTGGGGAGCAAACAGGATTAGATACCCTGGTAGTCCACGCCGTAAACGATGTCGACTTGG  300
Sbjct  771  GTGGGGAGCAAACAGGATTAGATACCCTGGTAGTCCACGCCGTAAACGATGTCGACTTGG  830
Query  301  AGGTTGTGCCCTTGAGGCGTGGCTTCCGGAGCTAACGCGTTAAGTCGACCGCCTGGGGAG  360
Sbjct  831  AGGTTGTGCCCTTGAGGCGTGGCTTCCGGAGCTAACGCGTTAAGTCGACCGCCTGGGGAG  890
Query  361  TACGGCCGCAAGGTTAAAACTTAAATGAATTGACGG  396
Sbjct  891  TACGGCCGCAAGGTTAAAACTCAAATGAATTGACGG  926
```
